# Supramolecular 3D Polymers Created via Chalcogen Bonding Using Benzotellurazoles as Planar Recognition Units

**DOI:** 10.1002/chem.202502731

**Published:** 2025-10-21

**Authors:** Jan Schulte, Saber Mehrparvar, Paul Kamminga, Christoph Wölper, Gebhard Haberhauer

**Affiliations:** ^1^ Fakultät für Chemie Universität Duisburg‐Essen, Universitätsstr. 7 Essen D‐45117 Germany

**Keywords:** chalcogen bond, DFT calculations, self‐assembly, supramolecular polymers

## Abstract

In addition to hydrogen and halogen bonds, chalcogen bonds (ChBs) are gaining significance in the domain of crystal engineering. Nitrogen‐containing tellurium compounds are well‐suited for this purpose, as they form a variety of solid‐state structures through ChBs. Despite prior studies addressing substituent effects in benzotellurazoles, the influence of fluorine atoms on the benzene backbone of 1,3‐benzotellurazoles remains to be investigated in the context of solid‐state arrangement. Here, we demonstrate that the incorporation of fluorine atoms can impact the nature and quantity of ChBs in the solid state. The electron‐withdrawing properties of the introduced substituents result in alterations to the ChB donor (Te) and acceptor (N) properties, leading to a preferential interaction with competing Lewis bases such as carbonyl groups. A noteworthy exception was noticed for the derivatives with a nitrile group at position 2. Regardless of the number of fluorine atoms, a high degree of 3D cross‐linking can be observed via a 5‐ChBs‐5‐neighbors motif, including a characteristic two‐lone‐pair/one‐σ‐hole interaction. These findings offer valuable design principles for supramolecular materials, particularly in the context of crystal engineering.

## Graphical abstract

Starting with small planar benzotellurazoles, it is possible to create 3D supramolecular architectures. These exhibit a high degree of cross‐linking, which is caused by various chalcogen bonding (ChB) motifs. Introducing electron‐withdrawing substituents onto the benzotellurazole makes the ChB motifs more apparent and results in more complex superstructures.

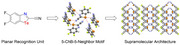

## 1 Introduction

During the past two decades, intensive research has been performed on the noncovalent interactions between a Lewis acidic chalcogen atom and Lewis bases. This type of interaction is also known as chalcogen bonding (ChB) interaction and corresponds to a σ‐hole interaction whereby electron density is shifted from one or two Lewis basic centers into the antibonding σ* orbital of a chalcogen‐R bond.^[^ ^1^, ^2^, ^3^, ^4^, ^5^, ^6^ ^]^ So far, ChBs have been successfully used in different research areas such as crystal engineering,^[^ ^7^, ^8^, ^9^, ^10^, ^11^, ^12^, ^13^, ^14^, ^15^, ^16^, ^17^, ^18^, ^19^, ^20^ ^]^ chalcogen‐bonded organic frameworks (ChOFs)^[^ ^21^, ^22^ ^]^ and polymer assemblies,^[^ ^23^, ^24^ ^]^ molecular recognition in solution,^[^ ^25^, ^26^, ^27^, ^28^, ^29^, ^30^, ^31^, ^32^, ^33^, ^34^, ^35^, ^36^, ^37^ ^]^ anion transport,^[^ ^38^, ^39^, ^40^ ^]^ catalysis,^[^ ^41^, ^42^, ^43^, ^44^, ^45^, ^46^ ^]^ and the modulation of photophysical properties in photoswitches.^[^ ^47^, ^48^, ^49^, ^50^, ^51^, ^52^ ^]^ Early systematic studies showed that the strength of the interaction depends both on the size of the chalcogen atom and on the substituent attached to the chalcogen atom. The heavier the chalcogen atom and the more electron‐withdrawing the substituent attached to the chalcogen atom, the stronger the corresponding ChB.^[^ ^2^, ^53^, ^54^, ^55^ ^]^ Accordingly, particularly strong chalcogen bonds (ChBs) are found in electron‐poor tellurium compounds such as isotellurazole oxides,^[^ ^14^, ^56^ ^]^ telluradiazole^[^ ^28^, ^57^, ^58^ ^]^ and benzotellurazoles.^[^ ^59^ ^]^ Since these heterocycles exhibit both Lewis acidic chalcogen atoms (Te) and Lewis basic centers (N), they can assemble via two ChBs and are therefore suitable as a rigid recognition unit for the design of supramolecular architectures. In the solid phase, the self‐assembly of these systems can lead to the formation of 1D and 2D polymers.

**Figure 1:**
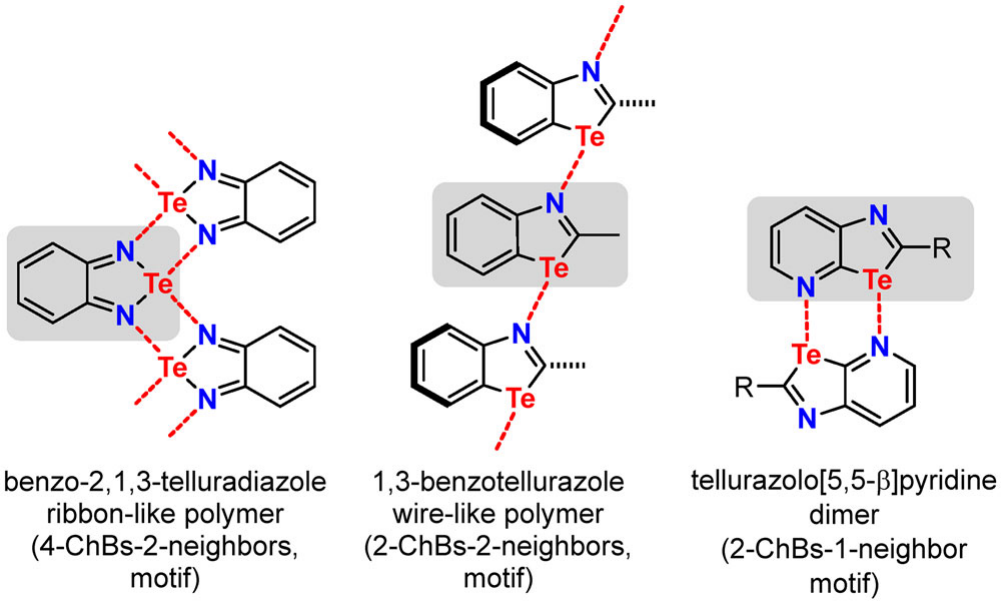
Examples of ChB‐based assemblies of tellurium‐containing nitrogen aromatics in the solid state. The bonding motifs range from 2‐ChBs‐1‐neighbor to 4‐ChBs‐2‐neighbors.

As an example, simple 1,3‐benzotellurazoles assemble in the solid state via a 2‐ChBs‐2‐neighbors motif, resulting in the formation of wire‐like (1D) supramolecular polymers (Figure 1).^[^ ^13^ ^]^ On the other hand, benzo‐2,1,3‐telluradiazoles form ribbon‐like 2D polymers in the solid phase, in which a recognition unit is connected to two neighbors via four ChBs (Figure 1; 4‐ChBs‐2‐neighbors motif).^[^ ^10^, ^57^ ^]^ 1,3‐Benzotellurazoles containing a carbonyl group at position 2 preferentially form Te⋅⋅⋅O interactions, whereby the two interacting molecules are positioned at an angle of 50–80° relative to each other. This results in the formation of 3D polymers such as helices,^[^ ^20^ ^]^ double helices^[^ ^20^ ^]^ and chalcogen‐bonded organic frameworks.^[^ ^21^ ^]^ In the case of tellurazolo[5,5‐b]pyridines, the structure in the solid state largely depends on the type of substituent at positions 2 and 5. For example, a dimer connected by two ChBs can be obtained (Figure 1; 2‐ChBs‐1‐neighbors motif).^[^ ^15^ ^]^ Repulsion of the substituents at positions 2 and 5 may result in the formation of wire‐like or ribbon‐like structures, in which each molecule is linked to two neighboring molecules via one ChB each (2‐ChBs‐2‐neighbors motif).^[^ ^17^ ^]^

**Figure 2:**
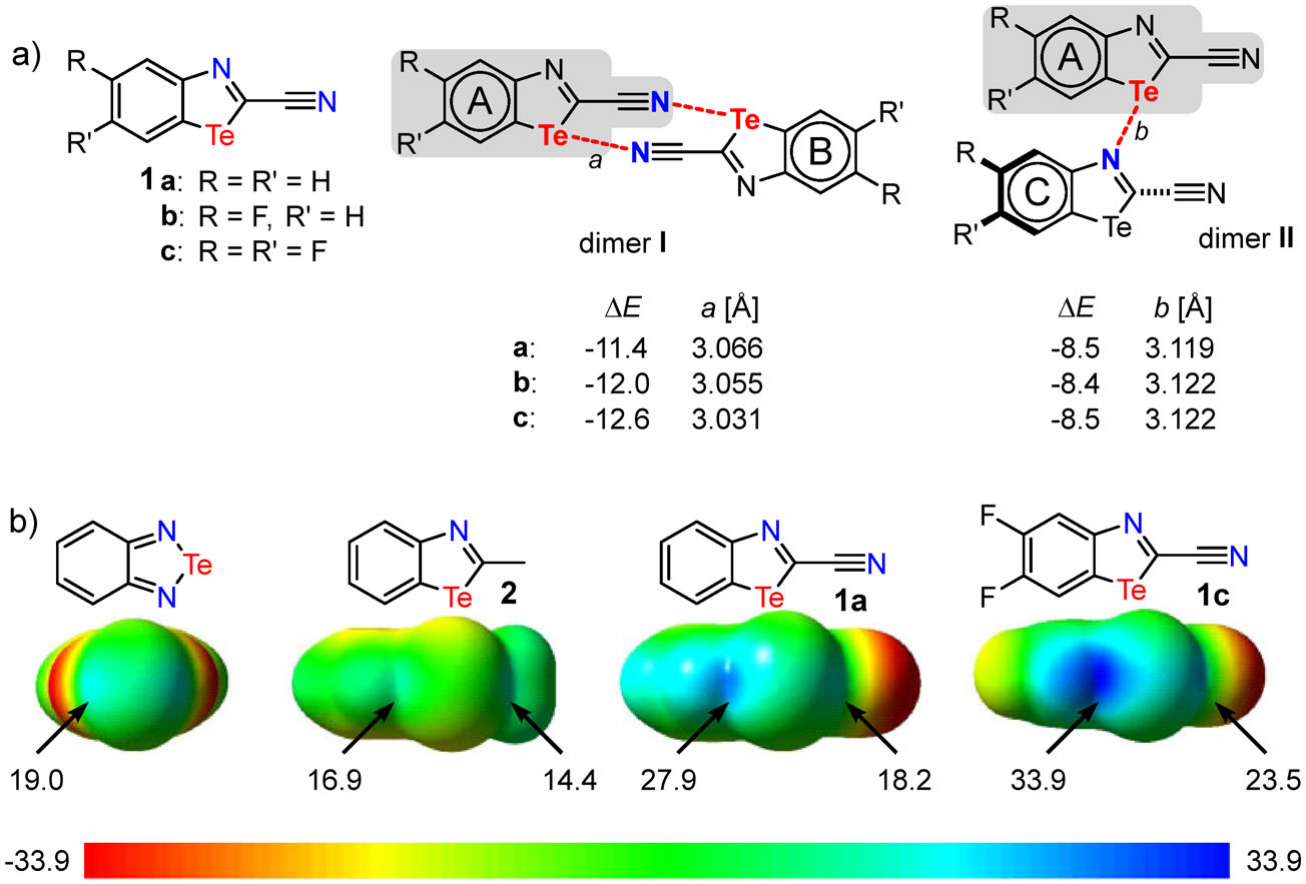
a) Cyanobenzotellurazoles **1** investigated in this study. In principle, these can form dimers **I** (two ChBs) and **II** (one ChB). The formation energies of the dimers (Δ*E*) and the Te⋅⋅⋅N distances calculated by means of B3LYP‐D3BJ are listed. The energy values are given in kcal/mol. b) Side views of ESP maps (calculated at B3LYP level of theory; *V*_s,max_ in kcal/mol) for benzo‐2,1,3‐telluradiazole and 1,3‐benzotellurazoles **1a**, **1c,** and **2**.^[^ ^61^ ^]^

Our intention was to design small planar recognition units that could form a 3D polymer in the solid phase exclusively through Te⋅⋅⋅N ChBs. In order to obtain such a polymeric architecture, it is necessary to design a system showing a large number of ChBs with their neighbors. We chose 2‐cyano‐1,3‐benzotellurazoles (Figure 2a) as candidates for this purpose, as they should form ChBs with the tellurium atoms via both the nitrogen atoms of the azole and the nitrogen atoms of the nitrile groups. The latter has already been demonstrated with a more flexible, nonplanar recognition unit.^[^ ^60^ ^]^

## 2 Results and Discussion

### 2.1 a) Concept and Synthesis

In order to obtain compounds showing a high number of ChBs in solid state, we intended to synthesize and investigate the benzotellurazoles **1a–c** (Figure 2a). These differ only in the number of fluorine atoms in the benzene ring of the benzotellurazole. We expect that the cyano group in **1a–c**, due to its electron‐withdrawing character, makes these compounds strong chalcogen bond donors. Within the series **1c**, having two electron‐withdrawing fluorine atoms should form the strongest ChBs. This can be seen from the electrostatic potential (ESP; Figure 2b). The *V*_s,max_ values of **1c** for both σ holes not only exceed the values of the methyl‐substituted benzotellurazole **2** but also those of benzo‐2,1,3‐telluradiazole (Figure 2b). To check whether **1c** with the highest *V*_s,max_ values also forms the most stable assemblies, dimers **I** and **II** (Figure 2a) were calculated by means of B3LYP‐D3BJ (see Supporting Information). A comparison of the dimerization energies for the type **I** dimers reveals that the strength of the assembly increases with the number of fluorine atoms, albeit only by 1.2 kcal/mol from **1a** to **1c**. The distance decreases slightly by 0.03 Å.

**Figure 3:**
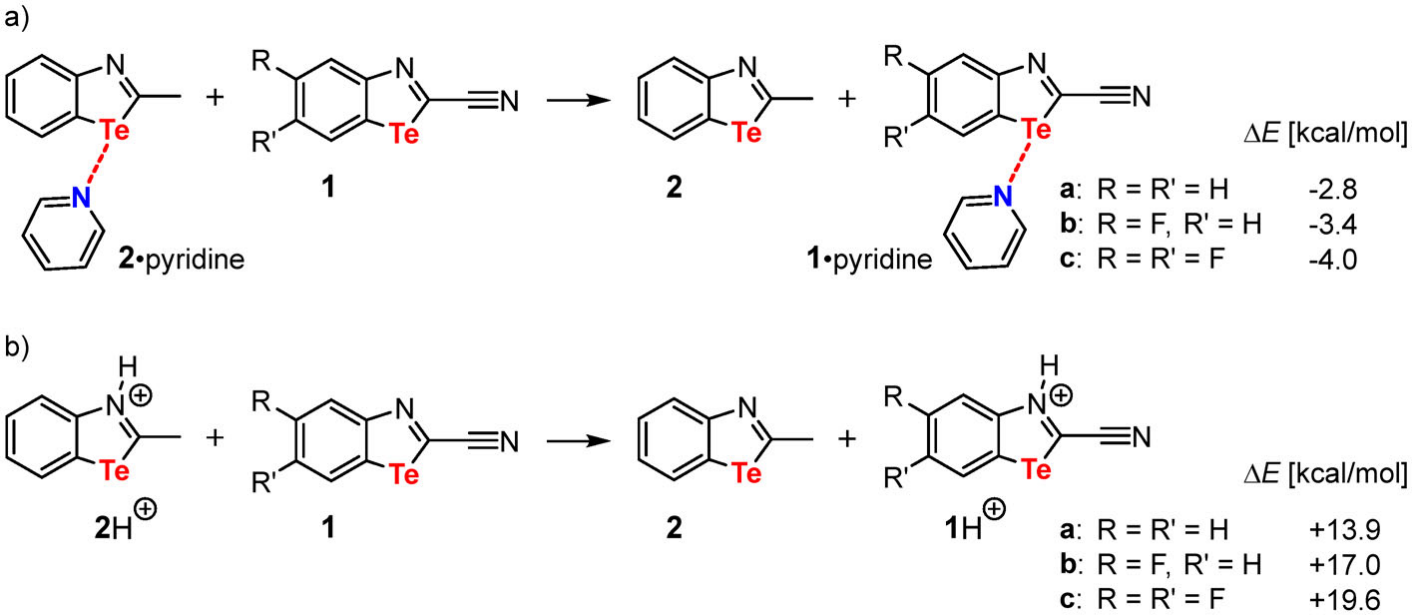
Isodesmic reactions to determine the strength of the chalcogen bonding (ChB) properties with pyridine a) and the basicity b) of the cyanobenzotellurazoles **1** relative to benzotellurazole **2** calculated at the B3LYP‐D3BJ level of theory.

A completely different picture emerges when considering dimers **II**. At first glance, it seems counterintuitive that the strength of the chalcogen bond in dimer **II**, unlike dimer **I**, is not affected by the fluorine substituents. However, the reason for this becomes obvious upon closer analysis, as can be demonstrated by isodesmic reactions (Figure 3). Using the easily accessible 2‐methyl‐1,3‐benzotellurazole (**2**)^[^ ^61^ ^]^ as a reference substance, the strength of the ChBs of **1** with pyridine increases with increasing number of fluorine atoms (Figure 3a), as *V*_s,max_ becomes more negative from **1a** to **1c** (Figure 2b). At the same time, however, the basicity of the nitrogen atom on the tellurazoles **1** decreases with increasing numbers of fluorine atoms due to their electron‐withdrawing character (Figure 3b). Overall, this leads to almost identical dimerization energies for the dimers **II** of **1a–c** (Figure 2a).

**Scheme 1:**
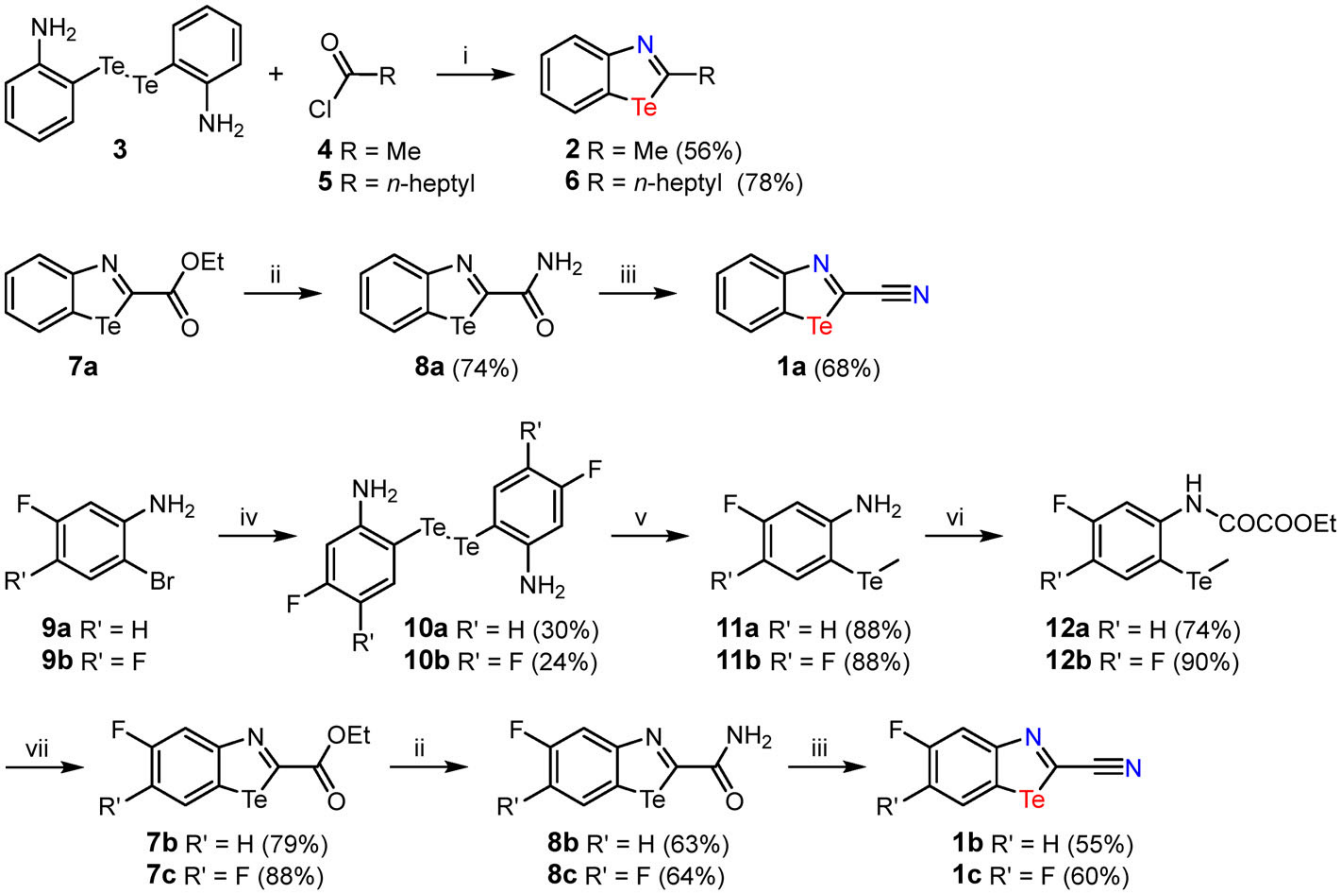
Synthesis of 2‐cyano‐1,3‐benzotellurazoles **1**. Reaction conditions: i) H_3_PO_2_, HCl_conc_, EtOH/THF, Δ. ii) NH_4_OH, EtOH. iii) POCl_3_, pyridine. iv) Te, NaH, NMP. v) MeI, NaBH_4_, MeOH/THF. vi) ClCOCOOEt, Et_3_N, DCM. vii) POCl_3_, Et_3_N, 1,4‐dioxane.

In addition to the cyano derivatives **1a–c**, we also intended to synthesize the reference compounds **2** and **6** (Scheme 1). These differ only in the size of the alkyl group at position 2. The synthesis of **2** was carried out analogously to the procedure of Junk et al.^[^ ^62^ ^]^ (Scheme 1). For this purpose, ditelluride **3** was reacted with the corresponding acid chloride in the presence of H_3_PO_2_ and HCl. The alkylbenzotellurazoles **2** and **6** were obtained in moderate yields.

The synthesis of **1a** was carried out from the already known ester **7a**.^[^ ^61^ ^]^ Reaction with an aqueous ammonia solution yields amide **8a**,^[^ ^20^ ^]^ which can be dehydrated to the nitrile **1a** in a yield of 68% using POCl_3_ in pyridine as a solvent. The fluorinated benzotellurazoles **1b** and **1c** were synthesized analogously to the procedure of Bonifazi et al.,^[^ ^13^ ^]^ using anilines **9a** and **9b** as starting materials (Scheme 1). The ditellurides **10** were converted via the anilines **11** into the anilides **12** in good overall yields. In the next step, the ethyl esters **7** were prepared using POCl_3_ and triethylamine in 1,4‐dioxane as a solvent. From the latter, the desired nitriles **1b** and **1c** were obtained in two steps.

### 2.2 b) Structures in Solid State and IQA Analyses

After successful synthesis, benzotellurazoles **1a–c**, **6**, **7b**, **7c**, **8b**, and **8c** were crystallized from a mixture of hexane and methylene chloride. Single crystals have been obtained in all cases and investigated by X‐ray diffraction. The solid‐state structures of **2**,^[^ ^63^ ^]^ **7a**, and **8a** ^[^ ^20^ ^]^ are already known, allowing a comparison of the structures depending on the substituents.

In addition to the investigations in the solid state, quantum theory of atoms in molecules (QTAIM)^[^ ^64^ ^]^ and interacting quantum atoms (IQA)^[^ ^65^ ^]^ analyses were performed. In this case, a benzotellurazole unit together with all neighbors, to which it is connected via ChBs, was defined as a model structure. This system was subsequently calculated, with the geometric parameters taken from the crystal structure data in order to represent the energy of the interacting atoms in solid state as accurately as possible. In the following, when comparing the interaction between two centers, the energies for the covalent part of the bond between the centers from IQA analysis are always referred to.

In the case of the 2‐alkyl‐substituted benzotellurazoles **2** and **6**, wire‐like structural patterns occur, forming a 2‐ChBs‐2‐neighbors motif (Figures S1 and S2). The distances of the Te⋅⋅⋅N interactions found in the crystal structure show no significant difference between the methyl‐substituted derivative **2** (3.153 Å) and the heptyl derivative **6** (3.108 Å). This means that although the alkyl residue in **6** is much larger than the methyl group in **2**, there is no change in the interaction pattern. Comparing both packings, an analogous type of pattern is found: The structure can be divided into blocks, with the alkyl groups of the benzotellurazole units aligned with each other in one block (Figures S3‐S6). The thickness of the blocks is greater in **6** (21.131 Å) than in **2** (10.818 Å) due to the large alkyl groups.

If we consider the solid‐state structures of esters **7a–c**, we find a significant change in structure within the series due to the introduction of fluorine atoms. In the nonfluorinated derivative **7a**, two independent molecules are found in the unit cell. Both form wire‐like structures, with Te⋅⋅⋅N and Te⋅⋅⋅O interactions occurring, resulting in a 4‐ChBs‐2‐neighbor motif (Figures S7 and S8). The interaction between the sp^3^‐hybridized oxygen of the ester function and the nitrogen atom takes place with one and the same σ hole. This is a so‐called two‐lone‐pair/one‐σ‐hole interaction. The distances between the tellurium and nitrogen atoms (3.158 Å and 3.301 Å, respectively) are significantly shorter than those to the oxygen (3.374 Å and 3.538 Å). The IQA calculations of the two 4‐ChBs‐2‐neighbor motifs show the same trend: The Te⋅⋅⋅N bond has values of ‐12.7 and ‐9.6 kcal/mol, respectively, while the Te⋅⋅⋅O interactions amount to only ‐6.4 and ‐4.6 kcal/mol, respectively.

**Figure 4:**
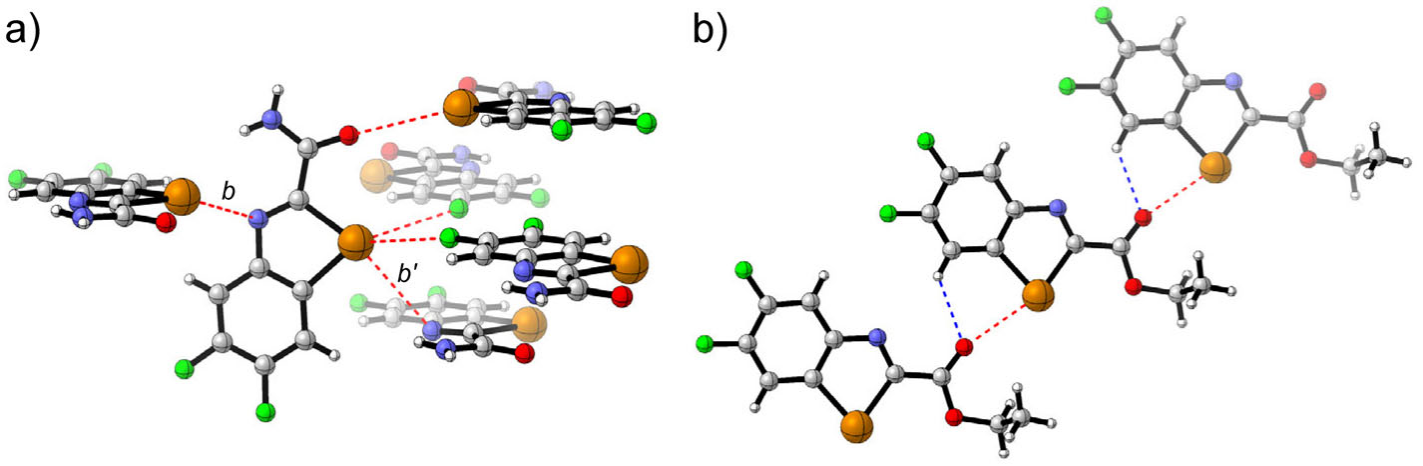
a) Illustration of one of three independent molecules of **8c** in the solid state, which displays a 6‐ChBs‐5‐neighbors motif. The six ChBs are marked in red. b) Ribbon‐like polymeric architectures of **7c** in solid state achieved by a 2‐ChBs‐2‐neighbors motif. The distance of the Te⋅⋅⋅O interaction (red) amounts to 2.925 Å. The hydrogen bonds between the carbonyl groups and the hydrogen of the aromatic rings are marked in blue.

Both fluorinated compounds, **7b** and **7c** feature ribbon‐like polymers with 2‐ChB‐2 neighbor motifs. These ribbons emerge as a result of additional hydrogen bonds (HB) between the oxygen atom of the carbonyl group and the opposite hydrogen atom of the aromatic compound (Figure 4b). Consequently, the benzotellurazoles bridged by ChB and HB lie in one plane. A similar arrangement has already been observed in tellurazolo[5,5‐b]pyridines, depending on the substitution pattern. However, in this case, the ribbon is formed by Te⋅⋅⋅N ChBs and N⋅⋅⋅H hydrogen bonds.^[^ ^17^ ^]^ The distance between the tellurium and the oxygen atom of the carbonyl group is smaller in the difluorinated system **7c** (2.925 Å) than in **7b** (3.026 Å). Analogous to the decrease in Te⋅⋅⋅O bond lengths of **7b** and **7c**, the covalent bond contribution of the Te⋅⋅⋅O interaction calculated using IQA also increases (‐12.1 kcal/mol for **7b** and ‐14.9 kcal/mol for **7c**).

Considering the packings of **7b** and **7c**, it becomes apparent that the ribbons are arranged differently from each other. In the case of **7b**, the ribbons lie in one plane, and the fluorine atoms of two adjacent ribbons are oriented toward each other (Figure S11). In **7c**, however, the ribbons are not in a plane but are arranged in a herringbone fashion at an angle of 30° to each other. The fluorine atoms of one ribbon are aligned with the ethyl groups of the neighboring ribbon (Figure S12).

In contrast to the wire‐ or ribbon‐like polymers of **7a–c**, the amides **8a–c** form a wide variety of bonding motifs in the solid state. The only bonding pattern common to all of them is the formation of HB‐bridged dimers (Figures S13–S15). However, the type of hydrogen bonds differs. In the case of the nonfluorinated amide **8a**, in addition to the CO⋅⋅⋅HN bridge, an N⋅⋅⋅HN bond is also formed with the nitrogen atom of the tellurazole (Figure S13). The fluorinated amides **8b** and **8c**, on the other hand, exhibited centrosymmetrical dimers formed by two CO⋅⋅⋅HN bridges (Figures S14, S15).

The already known benzotellurazole **8a** exhibits, in addition to the HB‐bridged dimers, a zigzag‐like polymeric architecture in the solid state achieved by a 2‐ChBs‐2‐neighbors motif (Figure S16). This zigzag pattern is due to the fact that the aromatic rings of two benzotellurazoles are not in the same plane, as in the case of **7b** and **7c**, but are oriented at an angle of 59° to each other. The Te⋅⋅⋅OC distance amounts to 3.060 Å, which is slightly larger than that in **7b** and **7c**. Nevertheless, the covalent bond contribution of the Te⋅⋅⋅O interaction calculated using IQA for **8a** is ‐13.1 kcal/mol, which lies between the values for **7b** and **7c**. This is consistent with previous findings, according to which the ideal angle between a carbonyl group and a benzotellurazole unit should be around 55–80°.^[^ ^20^, ^21^ ^]^

The monofluorinated amide **8b** contains no ChBs, only the HB‐bridged dimers mentioned above. The difluorinated amide **8c** shows three independent molecules with different ChB motifs. One of these molecules, together with its ChBs to its neighbors, is illustrated in Figure 4a. Overall, this represents a 5‐ChBs‐5‐neighbor motif. The nitrogen and oxygen atoms of the central unit interact with one tellurium center each, with distances of 3.235 Å (Te⋅⋅⋅N, *b* in Figure 4a) and 3.536 Å (Te⋅⋅⋅O). According to IQA calculations, the corresponding covalent bond energies have values of ‐11.0 kcal/mol and ‐5.2 kcal/mol, respectively. The tellurium atom of the central molecule even forms three ChBs with three neighbors: One ChB is a Te⋅⋅⋅N interaction (*b*' in Figure 4a) with a distance of 3.435 Å. Consequently, according to IQA calculations, this interaction (*b*') is significantly weaker at ‐7.2 kcal/mol than the other Te⋅⋅⋅N bond (*b*, ‐11.0 kcal/mol). The second σ hole of the tellurium atom forms ChBs with two fluorine atoms from different units, representing an intermolecular two‐lone‐pair/one‐σ‐hole interaction. The distances here amount to 3.305 Å and 3.335 Å; the corresponding values from the IQA calculations are ‐6.2 kcal/mol and ‐5.5 kcal/mol.

Due to the high number of ChBs with adjacent recognition units, there is a high degree of cross‐linking of the benzotellurazoles via ChBs in the solid state. Looking at the packing of **8c** in the direction of the *c* axis, two blocks of equal size (6.127 Å) but different arrangement and interaction type can be identified (Figure S17), with only Te⋅⋅⋅N and Te⋅⋅⋅F interactions occurring within a block. The blocks are held together by HBs, Te⋅⋅⋅O and Te⋅⋅⋅F interactions. This assembly can be explained as follows: Two types of interaction (Te⋅⋅⋅N and HB) are dominant due to their strength. The Te⋅⋅⋅N interactions lead to the formation of blocks, which are held together by HBs. The weaker ChBs, such as Te⋅⋅⋅O and Te⋅⋅⋅F stabilize the superstructure. In sum, there is a 3D polymeric architecture due to different Te⋅⋅⋅base interactions, with networking extending across the entire crystal.

**Figure 5:**
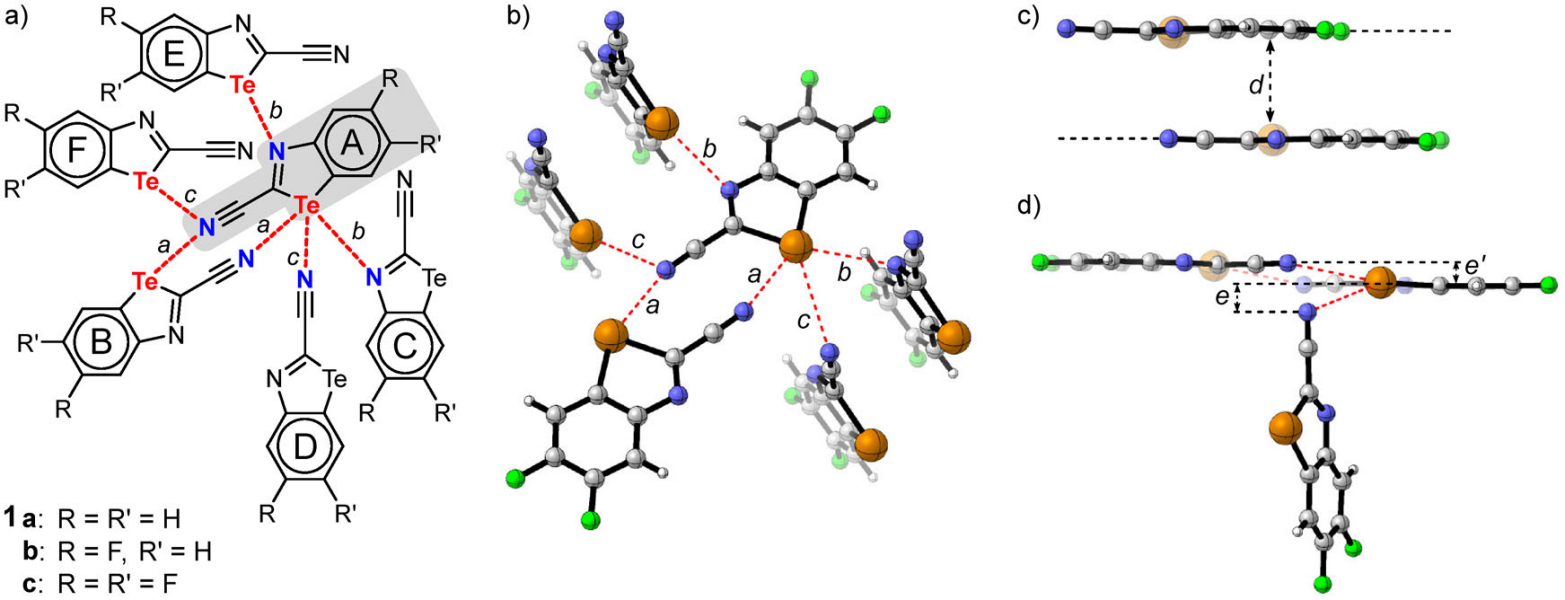
a) Schematic representation of the Te⋅⋅⋅N ChBs in the solid state of **1**, in which benzotellurazole A is connected to five further benzotellurazoles (B–F) via a 5‐ChBs‐5‐neighbor motif. Definition of the different Te⋅⋅⋅N interactions *a*–*c* in the 5‐ChBs‐5‐neighbor pattern. b) Illustration of the 5‐ChBs‐5‐neighbor motif in the solid state of **1c**. The Te⋅⋅⋅N interactions *a*, *b*, and *c* amount to 3.105 Å, 3.205 Å, and 3.499 Å, respectively. c) Definition of the distance *d* between parallel‐oriented aromatic units of **1c** in the solid state. d) The distances *e* and *e*' correspond to the minimum distance between the nitrogen atom of benzotellurazole B (*e*') and D (*e*) and the plane of benzotellurazole A.

Next, the solid‐state structures of nitriles **1a–c** will be considered. Here, the same cross‐linking pattern is always found: a benzotellurazole recognition unit (A in Figure 5a) is bound to five neighbors (B–F) via six ChBs (Figures 5, S18, S19). Three different types of Te⋅⋅⋅N interactions can be identified: The first are double ChBs forming dimers between benzotellurazoles A and B (*a* in Figure 5a), which correspond to the dimers **I** listed in Figure 2. The distance *a* decreases with increasing number of fluorine atoms: In the case of **1a**, it is 3.127 Å, while in **1c** it is only 3.105 Å. The absolute energy values calculated using IQA for the covalent part of the Te⋅⋅⋅N interaction also increase slightly from ‐13.0 kcal/mol for **1a** to ‐13.4 kcal/mol for **1c**. It is interesting to note that, in contrast to the calculated dimers **I**, the dimer formed from benzotellurazoles A and B does not lie in one plane but rather is arranged parallel to each other. The distance between these planes (*e*' in Figure 5d) ranges from 0.427 Å for **1a** to 0.527 Å for **1c**. The reason for this will be discussed below.

The second type (*b* in Figure 5a) is the interaction between the tellurium atom of one recognition unit and the nitrogen atom in the ring of another unit. This leads to the already known formation of wire‐like polymers and thus to the interaction type dimer **II** in Figure 2, in which the two benzotellurazole units are almost perpendicular to each other. Here, the distances increase with the number of fluorine atoms and range from 3.138 Å (**1a**) to 3.205 Å (**1c**). The corresponding energies determined by IQA decrease in magnitude from ‐13.3 kcal/mol (**1a**) through ‐12.9 kcal/mol (**1b**) to ‐11.6 kcal/mol (**1c**). The third type (*c* in Figure 5a) represents the interaction between the tellurium atom of one unit and the nitrile nitrogen of another unit, whereby the two benzotellurazoles are arranged almost perpendicular to each other. The distance *c* amounts to ca. 3.5 Å (**1a**: 3.491 Å; **1b**: 3.497 Å; **1c**: 3.499 Å) and is considerably longer than the distances *a* and *b*. Accordingly, the energy values from the IQA calculations for this Te⋅⋅⋅N interaction (approximately ‐6.5 kcal/mol) are smaller than those for *a* and *b*.

QTAIM analyses confirm the presence of a 5‐ChBs‐5‐neighbor motif for **1a–c**, as bond critical points are found for the six Te⋅⋅⋅N interactions in all cases (Figures S20–S22 and Tables S1–S3). This means that the tellurium atoms form three ChBs. Since divalent tellurium atoms exhibit two σ holes, this is only possible if a two‐lone‐pair/one‐σ‐hole interaction occurs in one case. This is why the two benzotellurazoles, A and B, do not lie in the same plane (Figure 5b). In order for the nitrile groups of units B and D to interact simultaneously with only one σ hole of the tellurium atom in benzotellurazole A, the two nitrogen atoms of the nitrile groups are not in the same plane as molecule A but are offset: The distances *e* and *e*' are approximately 0.5 Å for *e* and 1.0 Å for *e*'. To our knowledge, these are the first examples where a divalent tellurium atom forms three intermolecular Te⋅⋅⋅N interactions, whereby all three distances are smaller than 3.5 Å (for the definition of *a‐c* and their distances, see Figures 5b, S18, S19).

It is worth noting that in the case of **1a–c**, there is another interaction between the tellurium atom and a carbon atom of a staggered unit (Figure 5c). The distance found for *d* decreases with increasing number of fluorine atoms from 3.576 Å for **1a** through 3.509 Å for **1b** to 3.478 Å for **1c**. These distances are thus slightly below the sum of the van der Waals radii of the atoms (3.760 Å).^[^ ^66^ ^]^

**Figure 6:**
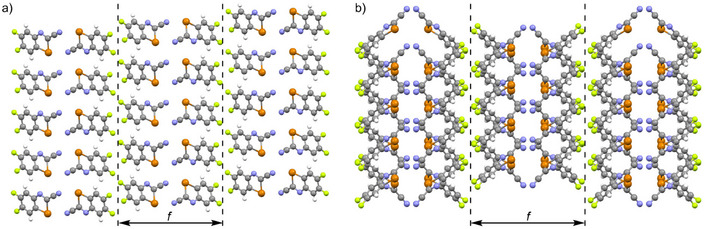
Solid‐state packing of benzotellurazole **1c**, viewed along the *b* (a) and *c* (b) axes. The block thickness *f* amounts to 13.952 Å.

Comparing the crystal packings of **1a–c**, one finds a pattern that has already been observed in **2** and **6**. The system can be divided into blocks, with the CN groups of the benzotellurazole units aligned with each other in each block (Figures 6, S23, S24). The thickness of the blocks *f* increases only slightly with increasing number of fluorine atoms, from 12.844 Å for **1a** through 13.468 Å for **1b** to 13.952 Å for **1c**. It is interesting to note that the Te⋅⋅⋅N interaction only occurs within the blocks. The 3D polymers based on ChBs thus decompose into blocks here, whereas in **7c** the ChBs are not limited to individual blocks.

### 2.3 c) Titrations With Lewis Bases

As mentioned at the beginning, according to calculations, the strength of the ChBs of benzotellurazoles **1a–c** should increase with an increasing number of fluorine atoms. Furthermore, due to the electron‐withdrawing character of the nitrile group, **1a** should be a better ChB donor than the methyl derivative **2** (see isodesmic reactions in Figure 3). To confirm this experimentally, we performed ^1^H NMR titration of the benzotellurazoles **1a–c** and **2** in deuterated chloroform. Preliminary performed ^1^H NMR dilution experiments showed that no dimerization of the benzotellurazoles occurs in chloroform.

**Figure 7:**
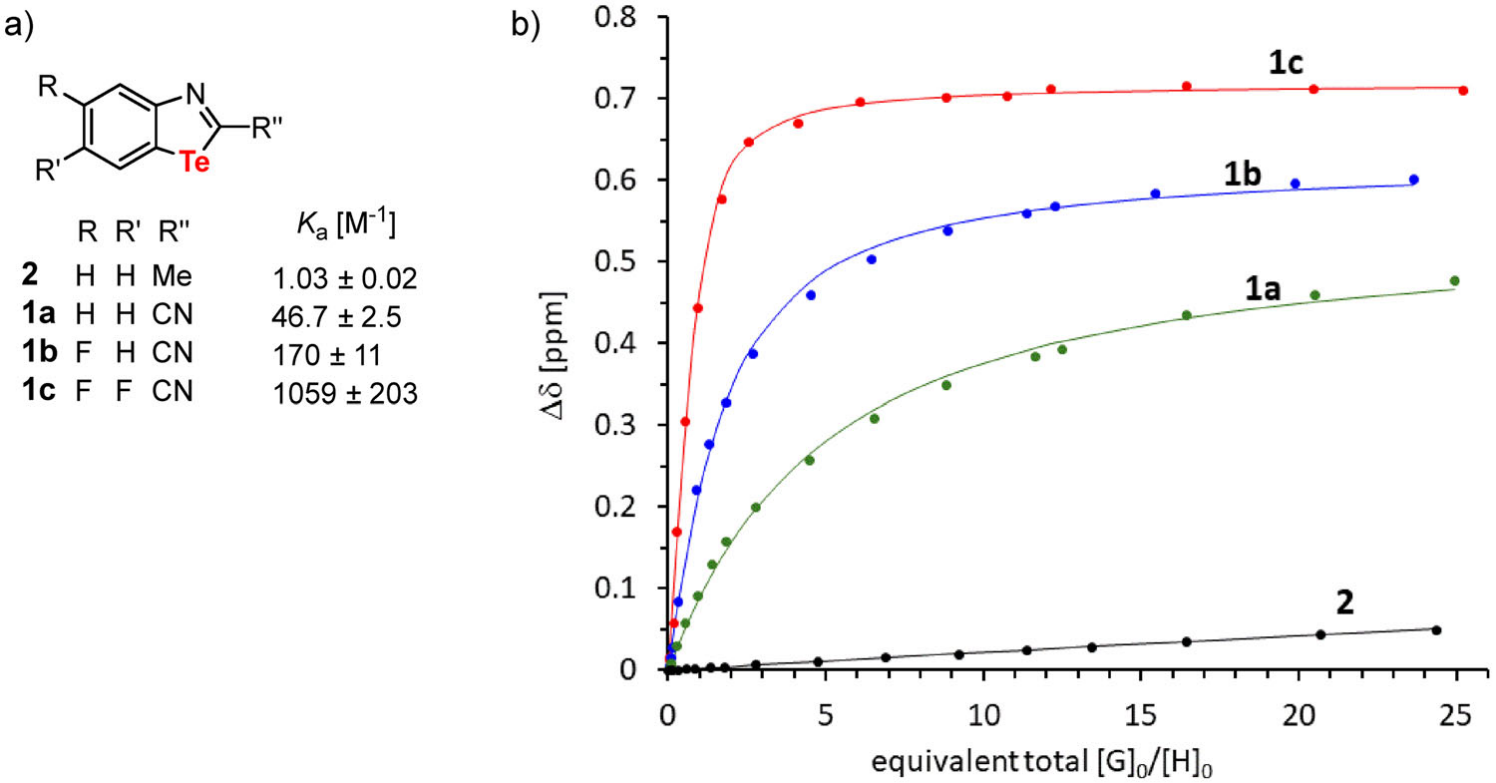
a) Binding constants (*K*_a_) from NMR titrations of compounds **1** and **2** with tetrabutylammonium fluoride (TBAF) in CDCl_3_. b) Plot of the change in chemical shift |Δδ| versus the equivalent total [G]_0_/[H]_0_ (guest: *n*‐Bu_4_N^+^F), fit to a 1:1 binding isotherm.

Since benzo‐2,1,3‐telluradiazoles have proven to provide good anion binding (e.g., chloride, bromide, iodide, etc.),^[^ ^28^ ^]^ we used tetrabutylammonium fluoride (TBAF) as a Lewis base. The results of the titrations are summarized in Figure 7. To ensure that the observed phenomenon (Figure S26) is the formation of a 1:1 complex, a Job's plot was generated for the complex of **1c** and *n*‐Bu_4_N^+^F^−^ (Figure S27). The maximum at 0.5 underlines the presence of a 1:1 complex.

While the methyl derivative **2** has a binding constant of only 1.0 m ^−1^ with TBFA, replacing the methyl group with a nitrile group (**1a**) leads to an almost 50‐fold increase in the association constant. The introduction of one fluorine atom (**1b**) leads to a threefold increase (compared to **1a**), while the introduction of two fluorine atoms (**1c**) leads to a 20‐fold increase in the constant. The binding constants of **2** and **1c** differ by a factor of 1000, which emphasizes the importance of substituents for the strength of ChBs in tellurium‐containing nitrogen cycles.^[^ ^28^ ^]^

In order to examine whether the trend toward complex formation can also be reproduced using quantum chemistry, the free energies of the complexes formed by benzotellurazoles **1a–c** and **2** with tetramethylammonium fluoride (TMAF) were calculated using B97‐D3^[^ ^67^ ^]^ and the basis set def2‐TZVP.^[^ ^68^ ^]^ To take solvent effects into account, the solvent model CPCM^[^ ^69^ ^]^ (chloroform as solvent) was used. This approximation was applied as it led to good agreement with experimental data for similar systems.^[^ ^28^ ^]^ The calculated values for the complexes of **1a–c** differ from the experimental values by only 1.0 kcal/mol (Table S4). For the fluorine complex of **2**, the deviation is significantly higher (2.7 kcal/mol). QTAIM calculations on the complexes show that the electron density at the ChB critical point slightly increases with increasing strength of the complexes (Table S5).

## 3 Conclusion

This study demonstrates the potential of substituting protons on the benzene backbone of 1,3‐benzotellurazoles with fluorine atoms to control the preferred solid‐state architecture. We show that, depending on the 2‐substituent, the introduction of one or two fluorine atoms leads to strikingly different chalcogen‐bonding motifs in the crystal structures. Quantum chemical calculations reveal that electron‐withdrawing substituents not only enhance the chalcogen bond donor ability but also reduce the Lewis basicity of the chalcogenazole nitrogen. Thus, in ester‐ and amide‐substituted systems, fluorination favors the interactions between the tellurium center and the carbonyl group of the 2‐residue. In contrast, when a nitrile substituent is present, the planar recognition units assemble into a 3D highly cross‐linked, polymer‐like architecture characterized by a 5‐ChBs‐5‐neighbors motif including a two‐lone‐pair/one‐σ‐hole interaction, regardless of the degree of fluorination. These results demonstrate how electronic tuning can be exploited to direct noncovalent interactions, offering valuable design principles for supramolecular chemistry and providing powerful guidelines for the development of functional supramolecular materials architectural design beyond self‐assembly or self‐assembly strategies for advanced applications.

## Supporting Information

Figures and tables, NMR spectra, synthesis of new compounds, computational details, crystal structure data and ^1^H NMR, ^13^C NMR and ^125^Te NMR spectra of the new compounds.

Deposition Numbers CCDC‐2483497 (for **1a**), ‐2483498 (for **1b**), ‐2483499 (for **1c**), ‐2483500 (for **2**), ‐2483501 (for **6**), ‐2483502 (for **7b**), ‐2483503 (for **7c**), ‐2483504 (for **8b**), ‐2483505 (for **8c**) contain the supplementary crystallographic data for this paper. These data are provided free of charge by the joint Cambridge Crystallographic Data Centre and Fachinformationszentrum Karlsruhe Access Structures service.

The authors have cited additional references within the Supporting Information.^[^ ^70^, ^71^, ^72^, ^73^, ^74^, ^75^, ^76^, ^77^, ^78^, ^79^ ^]^

## Conflict of Interest

There is no conflict of interest to declare.

## Supporting information



Supporting Information

Supporting Information

## Data Availability

The data that support the findings of this study are available in the supplementary material of this article.
