# Supplementary material for: Supramolecular 3D Polymers Created via Chalcogen Bonding Using Benzotellurazoles as Planar Recognition Units
Source: Chemistry. 2025 Oct 21;31(72):e02731. doi: 10.1002/chem.202502731 (PMC12731526; doi:10.1002/chem.202502731)
Supplement: Supplementary file 1 — Supporting Information [file CHEM-31-e02731-s001.pdf]

Supporting Information

Supramolecular Three-Dimensional Polymers Created via Chalcogen Bonding Using Benzotellurazoles as Planar Recognition Units

Jan Schulte†, Saber Mehrparvar†, Paul Kamminga†, Christoph Wölper† and Gebhard Haberhauer*†

† Institut für Organische Chemie, Universität Duisburg-Essen, Universitätsstr. 7, D-45117 Essen, Germany

1. **Figures and Tables** S2
2. **Synthesis of New Compounds** S20
3. **Computational Details** S32
4. **Crystal Structure Data** S46
5. **^{1}H NMR, ^{13}C NMR and ^{125}Te NMR Spectra of the New Compounds** S64

1. Figures and Tables

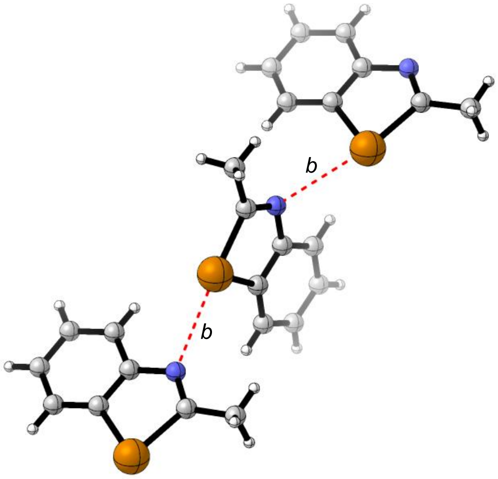

Figure S1. Wire-like polymeric architectures of **2** in solid state achieved by a 2-ChBs-2-neighbors motif. The distance *b* of the Te···N interaction amounts to 3.153 Å.

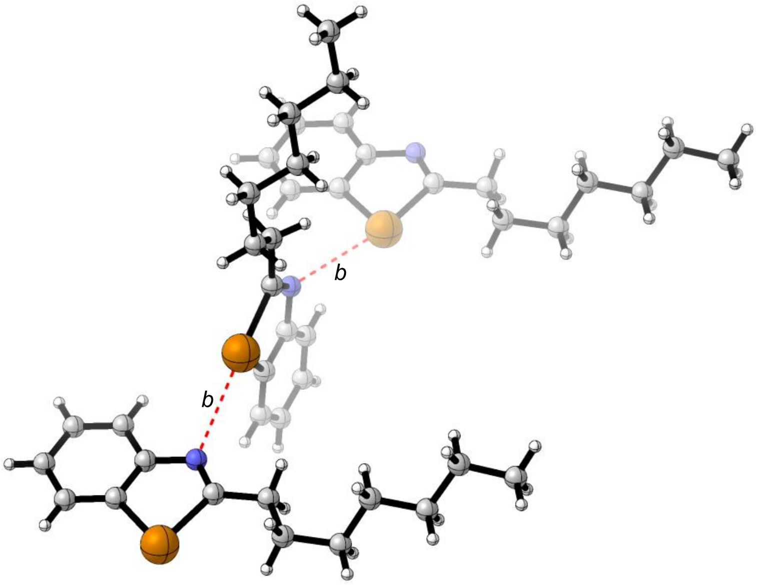

Figure S2. Wire-like polymeric architectures of **6** in solid state achieved by a 2-ChBs-2-neighbors motif. The distance *b* of the Te···N interaction amounts to 3.108 Å.

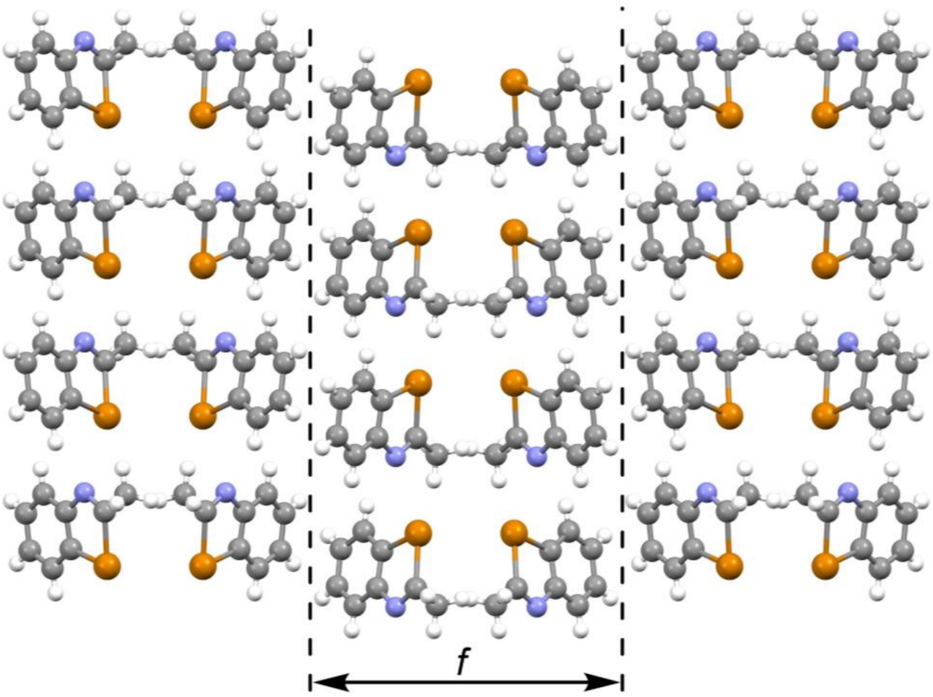

Figure S3. Solid-state packing of benzotellurazole **2**, viewed along the *a* axis. The block thickness *f* amounts to 10.818 Å.

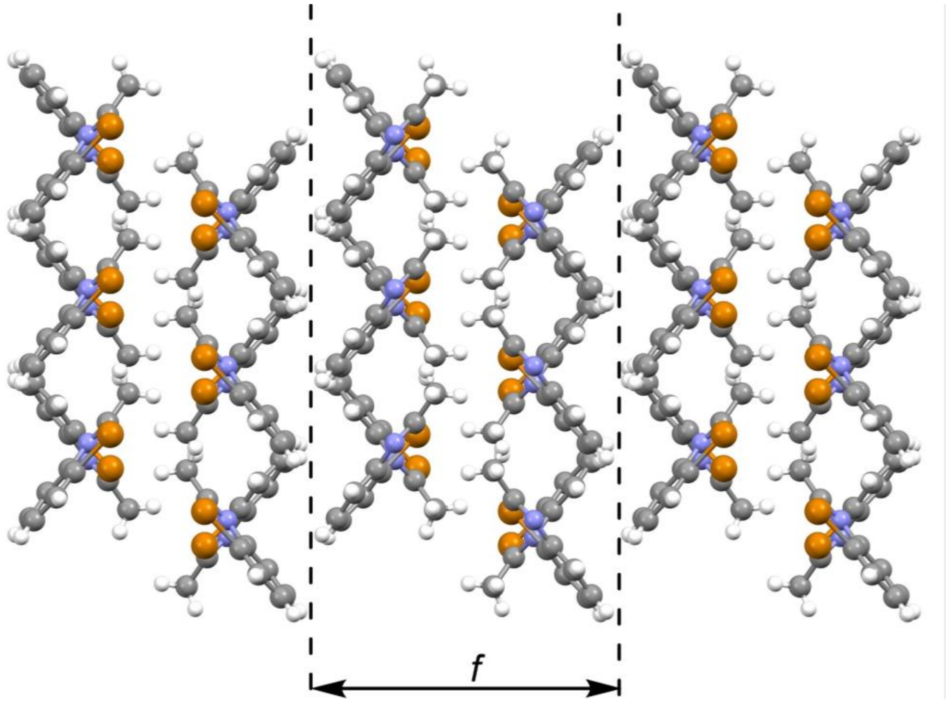

Figure S4. Solid-state packing of benzotellurazole **2**, viewed along the *b* axis. The block thickness *f* amounts to 10.818 Å.

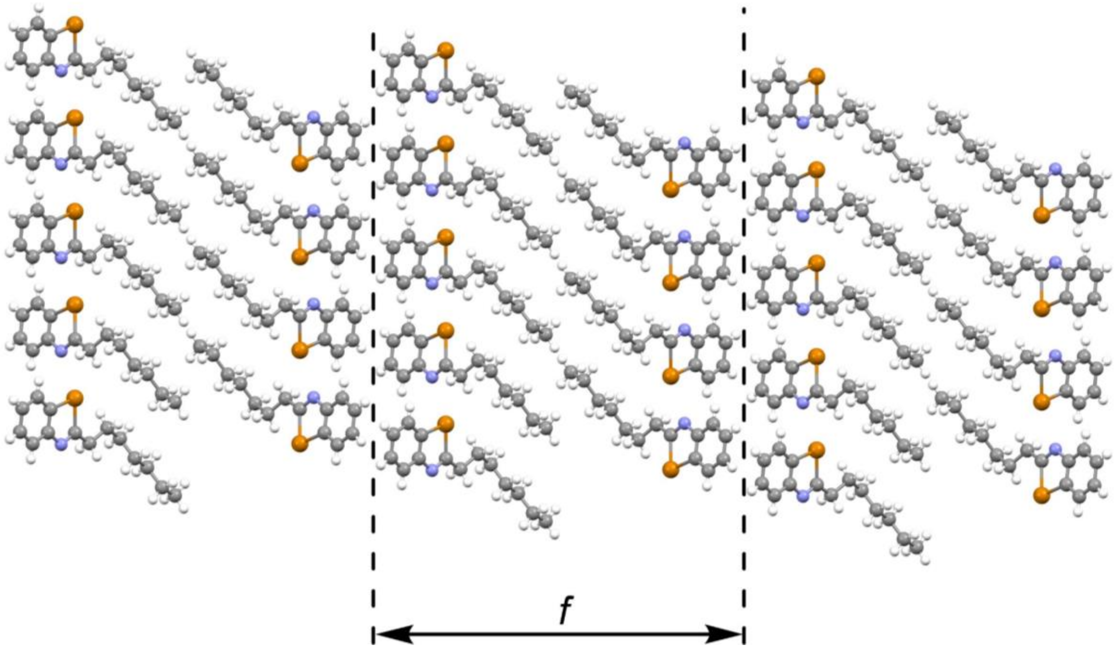

Figure S5. Solid-state packing of benzotellurazole **6**, viewed along the *b* axis. The block thickness *f* amounts to 21.131 Å.

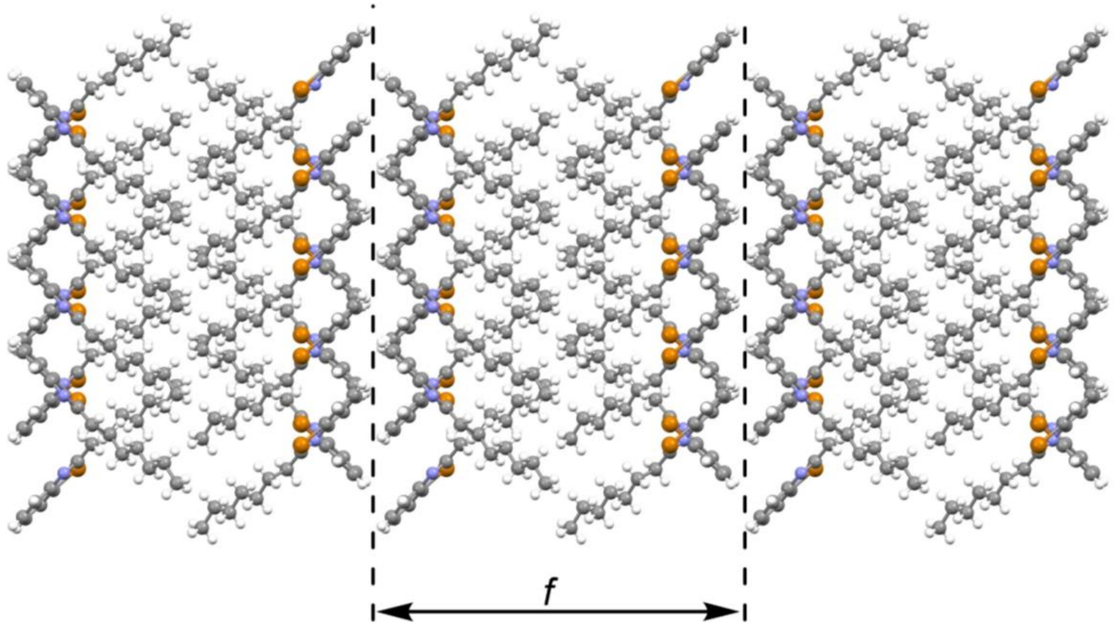

Figure S6. Solid-state packing of benzotellurazole **6**, viewed along the *c* axis. The block thickness *f* amounts to 21.131 Å.

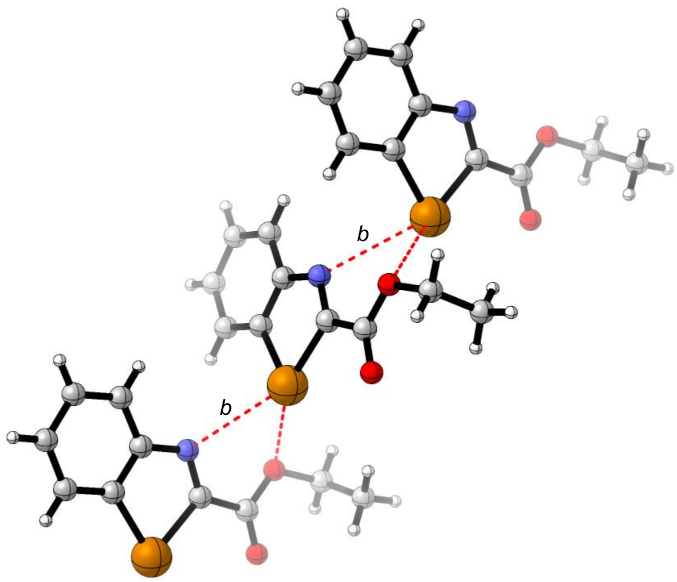

Figure S7. The first of two wire-like polymers of compound **7a** in the solid state achieved by a 4-ChBs-2-neighbors motif. The distance *b* of the Te···N interaction amounts to 3.158 Å; the Te···O bond is to 3.374 Å.

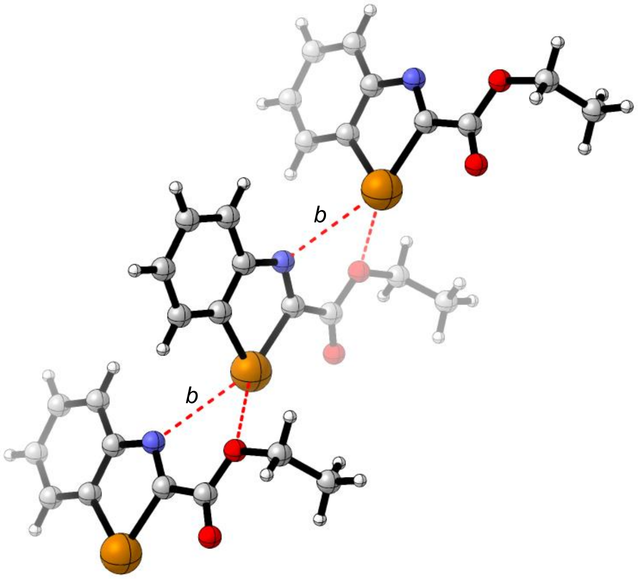

Figure S8. The second of two wire-like polymers of compound **7a** in the solid state achieved by a 4-ChBs-2-neighbors motif. The distance *b* of the Te···N interaction amounts to 3.301 Å; the Te···O bond is to 3.538 Å.

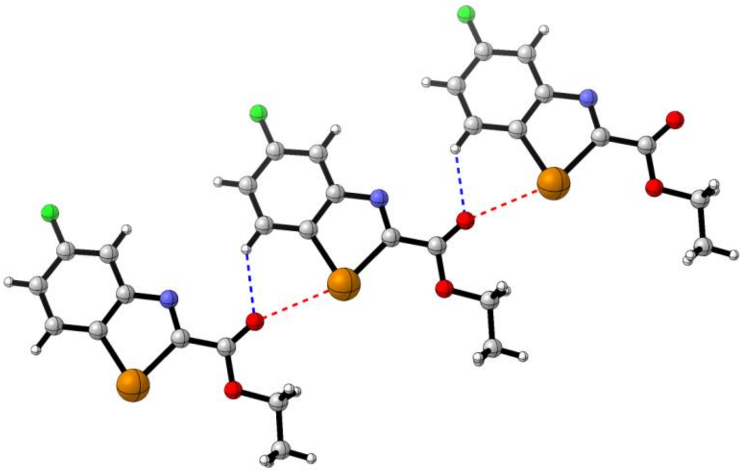

Figure S9. Ribbon-like polymeric architectures of **7b** in solid state achieved by a 2-ChBs-2-neighbors motif. The distance of the Te···O interaction (red) amounts to 3.026 Å. The hydrogen bond between the carbonyl groups and the hydrogen of the aromatic rings are marked in blue.

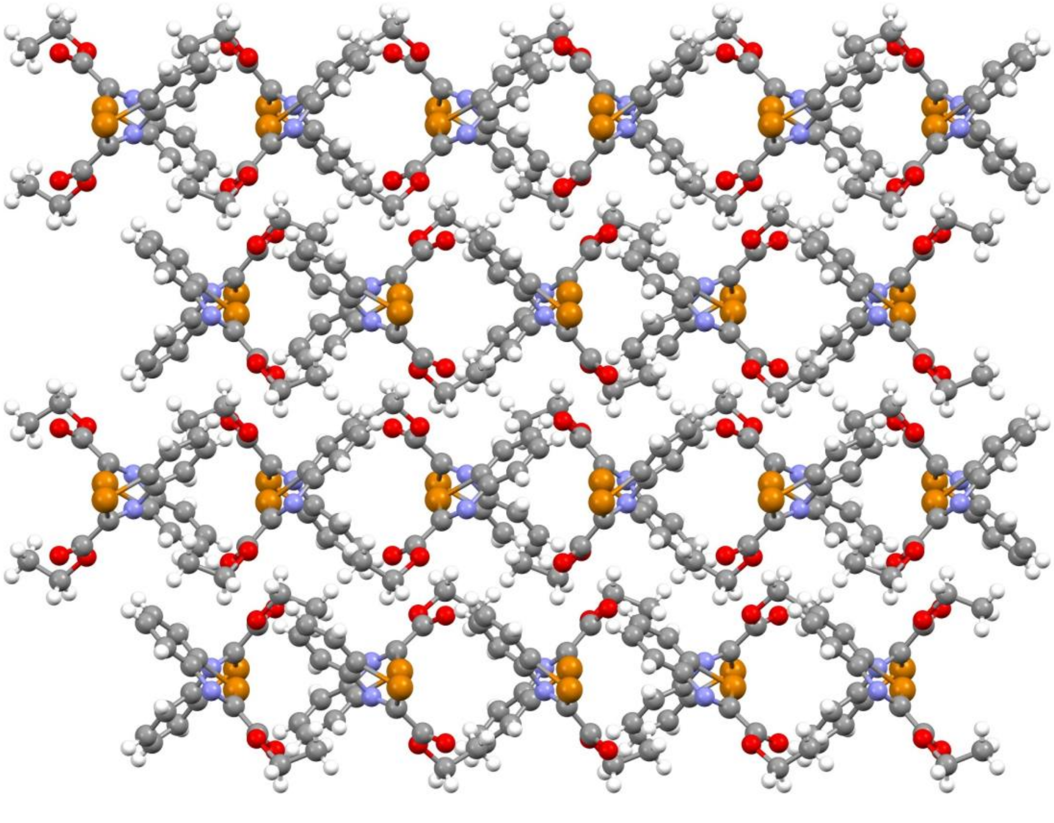

Figure S10. Solid-state packing of benzotellurazole **7a**, viewed along the *b* axis.

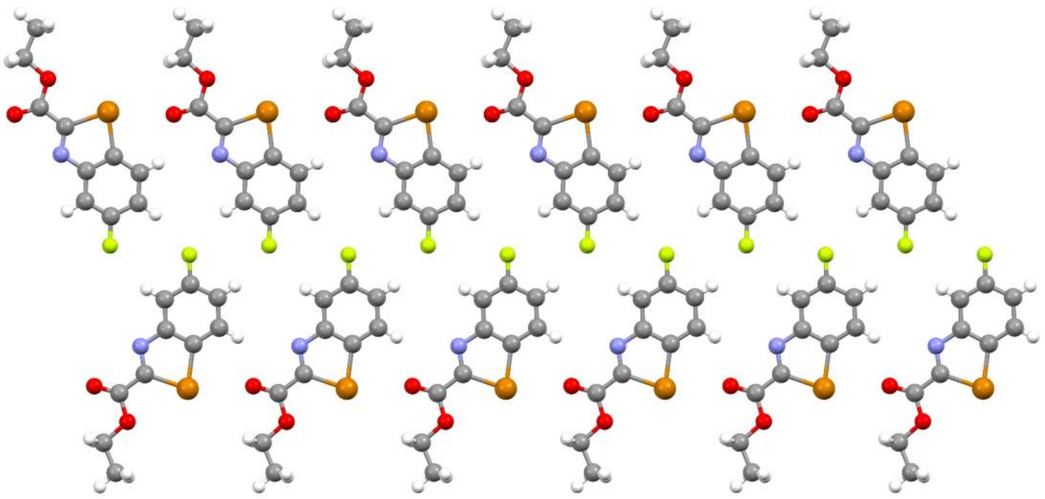

Figure S11. In the solid state of **7b**, the ribbon-like polymers formed via ChBs and HBs lie in one plane.

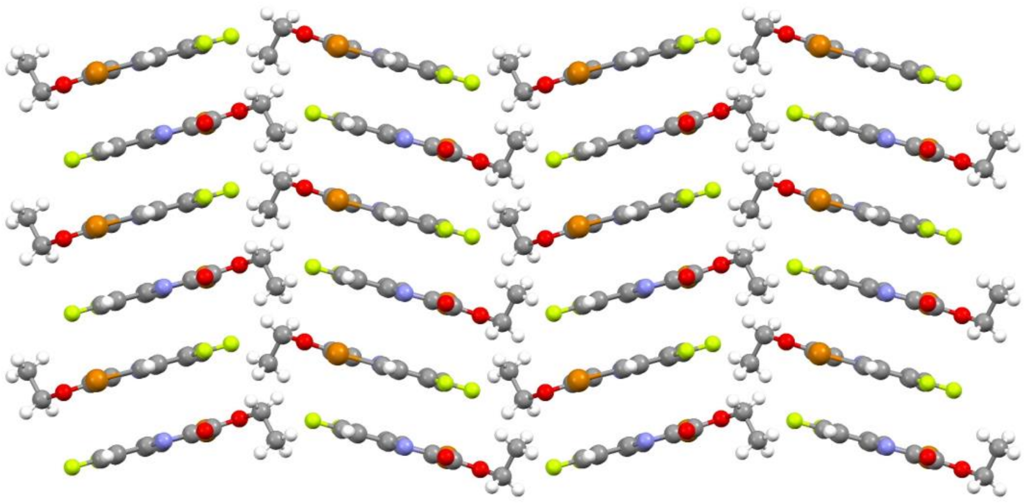

Figure S12. In the solid state of **7c**, the ribbon-like polymers formed via ChBs and HBs are arranged in a herringbone fashion.

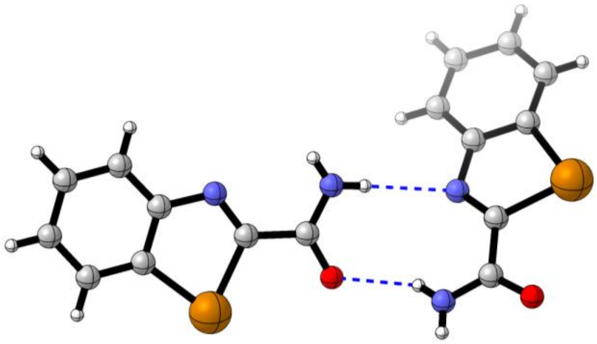

Figure S13. Dimer formation through double hydrogen bonds (marked in blue) in the crystal structure of amide **8a**.

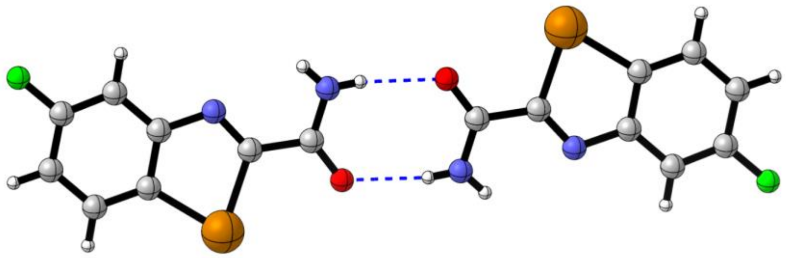

Figure S14. Dimer formation through double hydrogen bonds (marked in blue) in the crystal structure of amide **8b**.

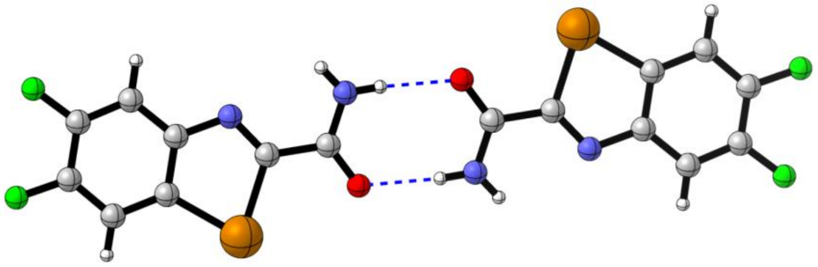

Figure S15. Dimer formation through double hydrogen bonds (marked in blue) in the crystal structure of amide **8c**.

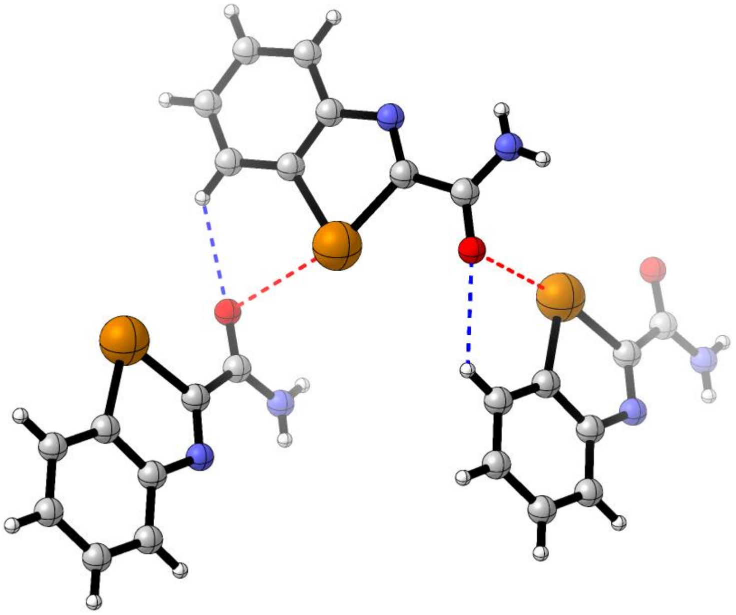

Figure S16. Zigzag-like polymeric architectures of **8a** in solid state achieved by a 2-ChBs-2-neighbors motif. The distance of the Te···O interaction (red) amounts to 3.060 Å. The hydrogen bond between the carbonyl groups and the hydrogen of the aromatic rings are marked in blue.

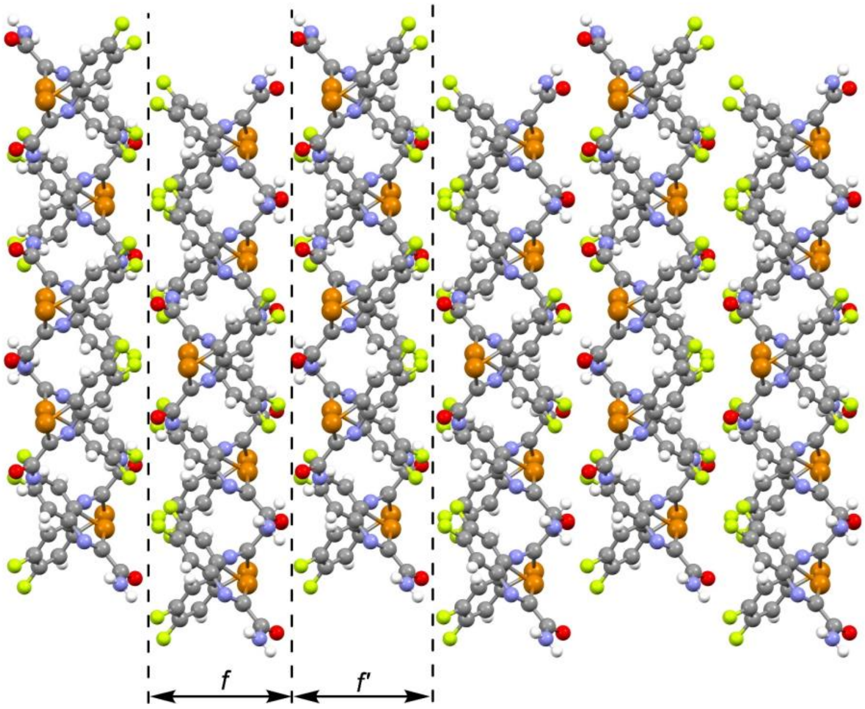

Figure S17. Solid-state packing of benzotellurazole **8c**, viewed along the *c* axis. The block thickness *f* and *f*' amount to 6.127 Å.

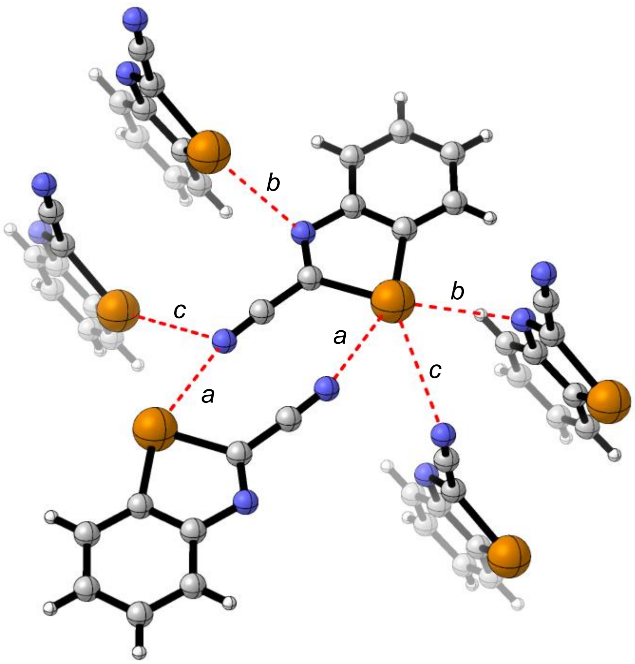

Figure S18. Representation of the 5-ChBs-5-neighbor motif in the solid state of **1a**. The Te···N interactions *a*, *b* and *c* amount to amount to 3.127 Å, 3.138 Å and 3.491 Å, respectively.

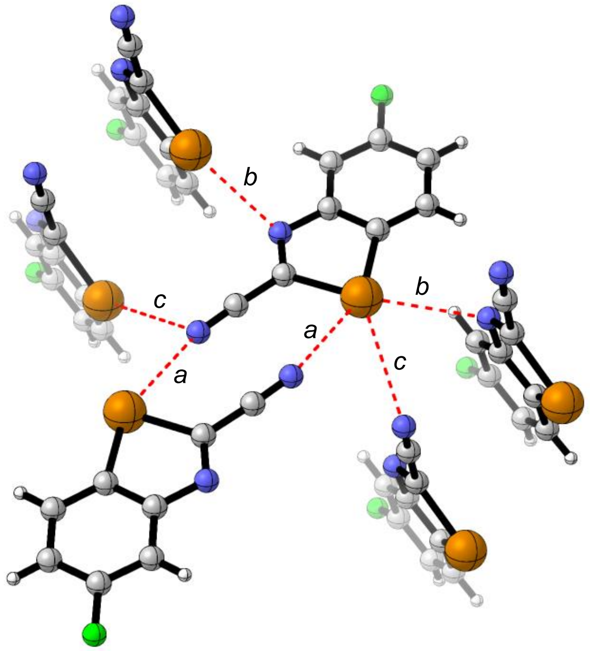

Figure S19. Representation of the 5-ChBs-5-neighbor motif in the solid state of **1b**. The Te···N interactions *a*, *b* and *c* amount to amount to 3.120 Å, 3.148 Å and 3.497 Å, respectively.

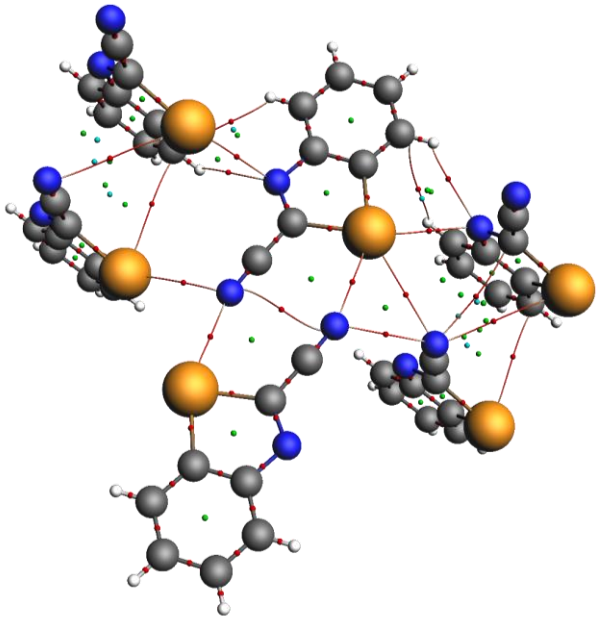

Figure S20. Quantum theory of atoms in molecules (QTAIM) analysis of the 5-ChBs-5-neighbor motif of **1a** (B3LYP-D3BJ/TZ2P). Bond critical points are shown in red, ring critical points in green and cage critical points in light-blue.

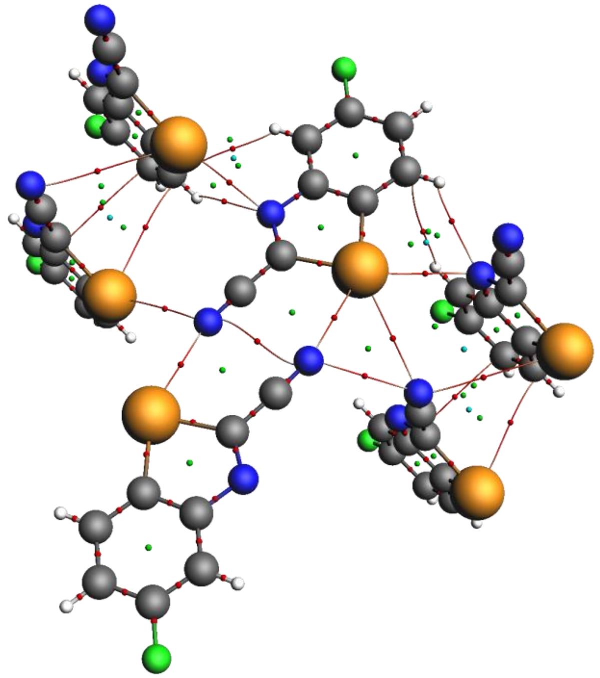

Figure S21. Quantum theory of atoms in molecules (QTAIM) analysis of the 5-ChBs-5-neighbor motif of **1b** (B3LYP-D3BJ/TZ2P). Bond critical points are shown in red, ring critical points in green and cage critical points in light-blue.

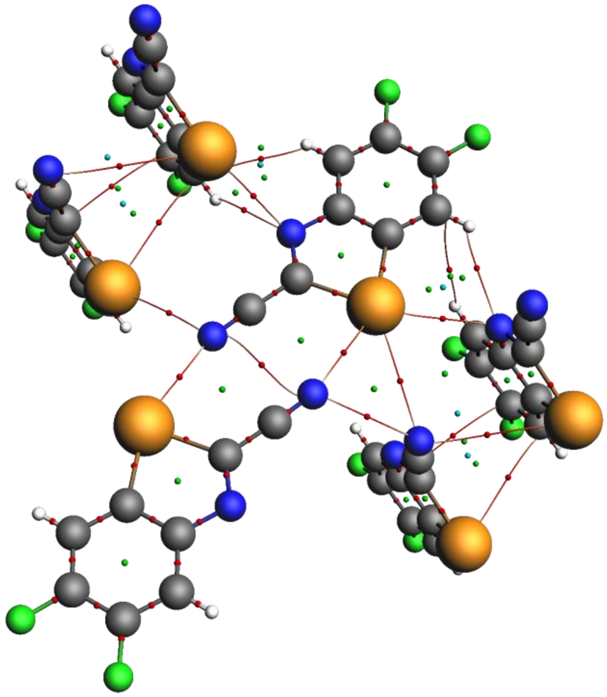

Figure 22. Quantum theory of atoms in molecules (QTAIM) analysis of the 5-ChBs-5-neighbor motif of **1c** (B3LYP-D3BJ/TZ2P). Bond critical points are shown in red, ring critical points in green and cage critical points in light-blue.

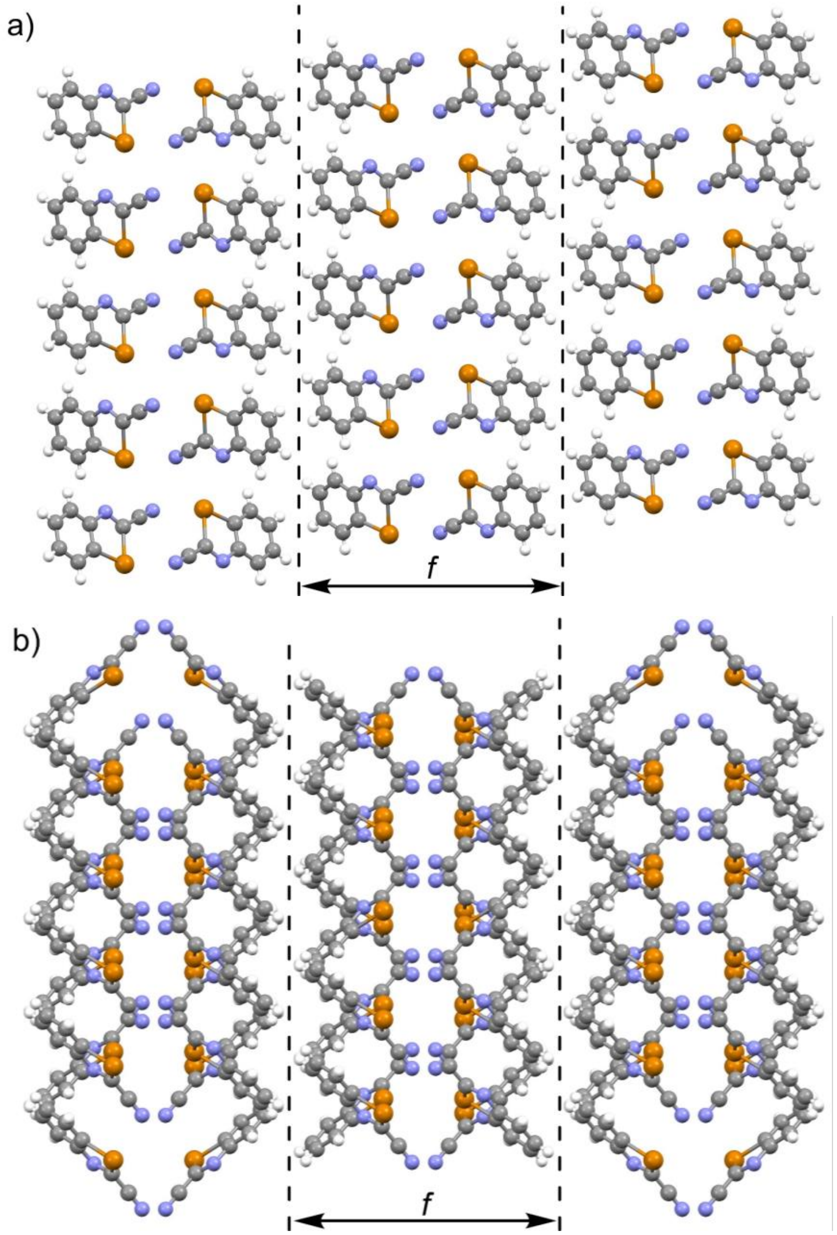

Figure S23. Solid-state packing of benzotellurazole **1a**, viewed along the *b* (a) and *c* (b) axes. The block thickness *f* amounts to 12.844 Å.

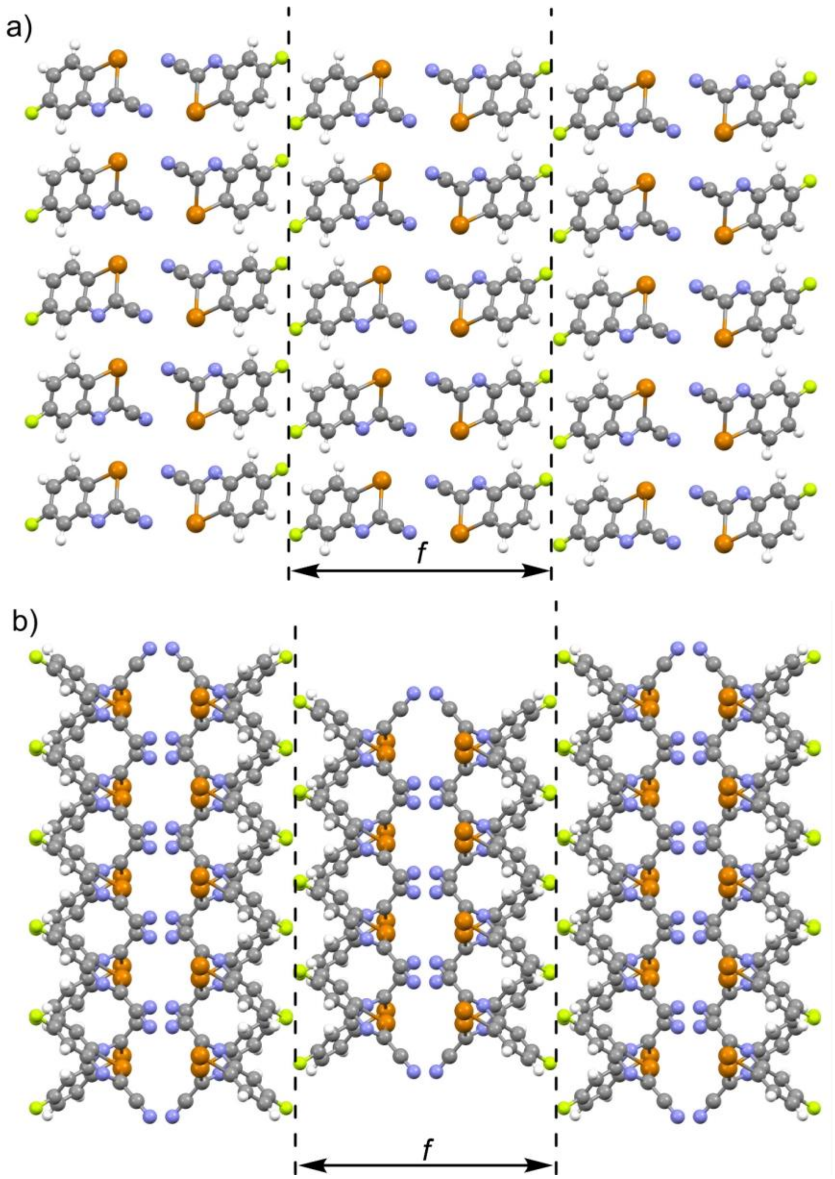

Figure S24. Solid-state packing of benzotellurazole **1b**, viewed along the *b* (a) and *c* (b) axes. The block thickness *f* amounts to 13.468 Å.

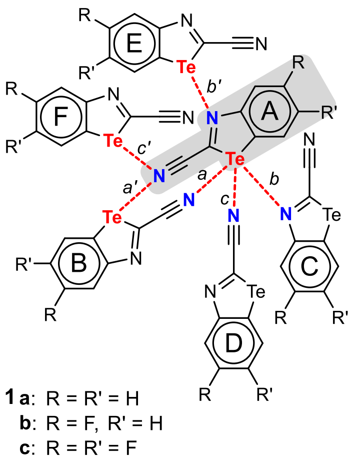

Figure S25. Schematic representation of the Te···N chalcogen bonds in the solid state of **1**, in which the recognition unit A is connected to five further units (B–F) via a 5-ChBs-5-neighbor motif. Definition of the different Te···N interactions *a*–*c* in the 5-ChBs-5-neighbor pattern.

Table S1. Electron density (ρ_{CP}) calculated at the bond critical points and interaction energies ($V_{inter(Total)}$) between the two atoms of the Te···N interactions in the hexamer of **1a** calculated by means of B3LYP-D3BJ/TZ2P. The electron densities (ρ_{CP}) are given in a.u. The energies are given in kcal/mol. The Te···N interactions a-c and a'-c' are defined in Figure S25.

Te···N interaction	ρ_{CP}	$V_{inter(Total)}$	Ionic part	Covalent part
a	0.01385341	-111.49	-98.95	-12.54
b	0.01500485	-104.82	-92.01	-12.81
c	0.07282842	-85.12	-79.33	-5.79
a‘	0.01388267	-102.63	-89.28	-13.35
b‘	0.01522225	-94.46	-80.70	-13.76
c‘	0.00767722	-75.04	-67.71	-7.32

Table S2. Electron density (ρ_{CP}) calculated at the bond critical points and interaction energies ($V_{inter(Total)}$) between the two atoms of the Te···N interactions in the hexamer of **1b** calculated by means of B3LYP-D3BJ/TZ2P. The electron densities (ρ_{CP}) are given in a.u. The energies are given in kcal/mol. The Te···N interactions a-c and a'-c' are defined in Figure S25.

Te···N interaction	ρ_{CP}	$V_{inter(Total)}$	Ionic part	Covalent part
a	0.01401358	-113.86	-101.30	-12.56
b	0.01472728	-105.62	-93.15	-12.47
c	0.07224113	-85.98	-80.24	-5.74
a'	0.01402991	-105.65	-92.24	-13.41
b'	0.01491691	-94.84	-81.51	-13.34
c'	0.00761069	-76.09	-68.88	-7.21

Table S3. Electron density (ρ_{CP}) calculated at the bond critical points and interaction energies ($V_{inter(Total)}$) between the two atoms of the Te···N interactions in the hexamer of **1c** calculated by means of B3LYP-D3BJ/TZ2P. The electron densities (ρ_{CP}) are given in a.u. The energies are given in kcal/mol. The Te···N interactions a-c and a'-c' are defined in Figure S25.

Te···N interaction	ρ_{CP}	$V_{inter(Total)}$	Ionic part	Covalent part
a	0.01438802	-116.02	-102.95	-13.06
b	0.01327218	-102.62	-91.41	-11.21
c	0.00717777	-87.08	-81.38	-5.70
a'	0.01438825	-108.36	-94.54	-13.82
b'	0.01346344	-92.03	-80.01	-12.02
c'	0.00758173	-76.91	-69.71	-7.20

Table S4. Experimental ($\Delta G_{exp.}$) and calculated free energies ($\Delta G_{B97\text{-}D3}$) of the complexes of the benzotellurazoles **1a–c** and **2** with fluoride in chloroform as solvent. The energies are given in kcal/mol.

Complex	$\Delta G_{exp.}$[a]	$\Delta G_{B97\text{-}D3}$[b]
**2**•F^-	0.0	2.7
**1a**•F^-	-2.3	-3.3
**1b**•F^-	-3.0	-4.2
**1c**•F^-	-4.1	-5.1

[a] Tetrabutylammonium fluoride (TBAF) was used. [b] Tetramethylammonium fluoride (TMAF) was used.

Table S5. Electron density (ρ_{CP}) calculated at the bond critical points and interaction energies ($V_{inter(Total)}$) between the two atoms of the Te···F interactions in the complexes of the benzotellurazoles **1a–c** and **2** with tetramethylammonium fluoride (TMAF) calculated by means of B3LYP-D3BJ/TZ2P//B97-D3(CPCM,$CHCl_3$/def2-TZVP. The electron densities (ρ_{CP}) are given in a.u. The energies are given in kcal/mol.

Complex	ρ_{CP}	$V_{inter(Total)}$	Ionic part	Covalent part
**2**•TMAF	0.04121409	-136.59	-93.99	-42.60
**1a**•TMAF	0.06372024	-209.66	-145.26	-64.39
**1b**•TMAF	0.06414914	-211.27	-146.61	-64.66
**1c**•TMAF	0.06419583	-211.69	-147.13	-64.55

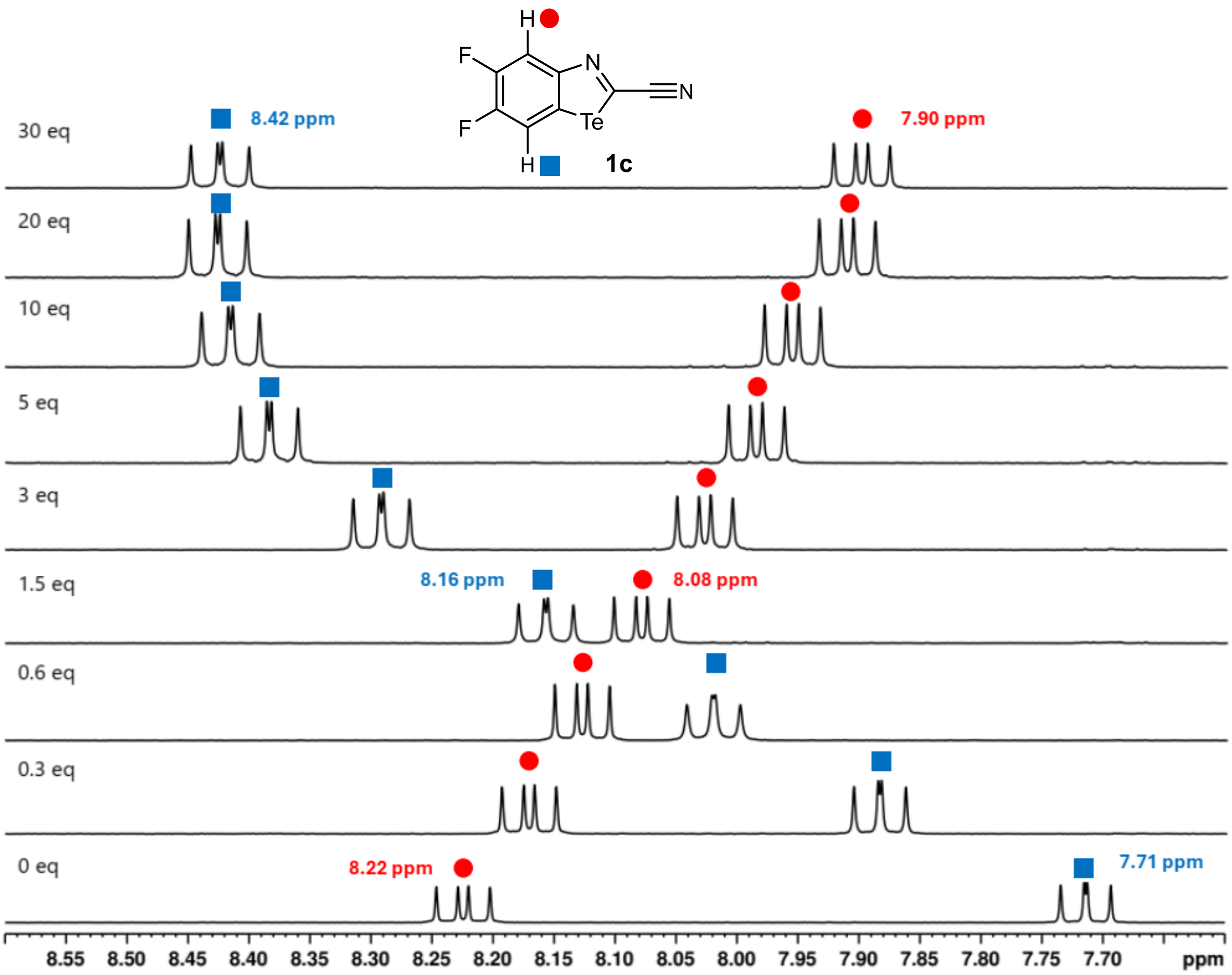

Figure S26. Section from the ^{1}H NMR spectra of **1c** with different equivalents of *n*-$Bu_4N^+F^-$ ($CDCl_3$, 400 MHz, c(**1c**) = 5 mM).

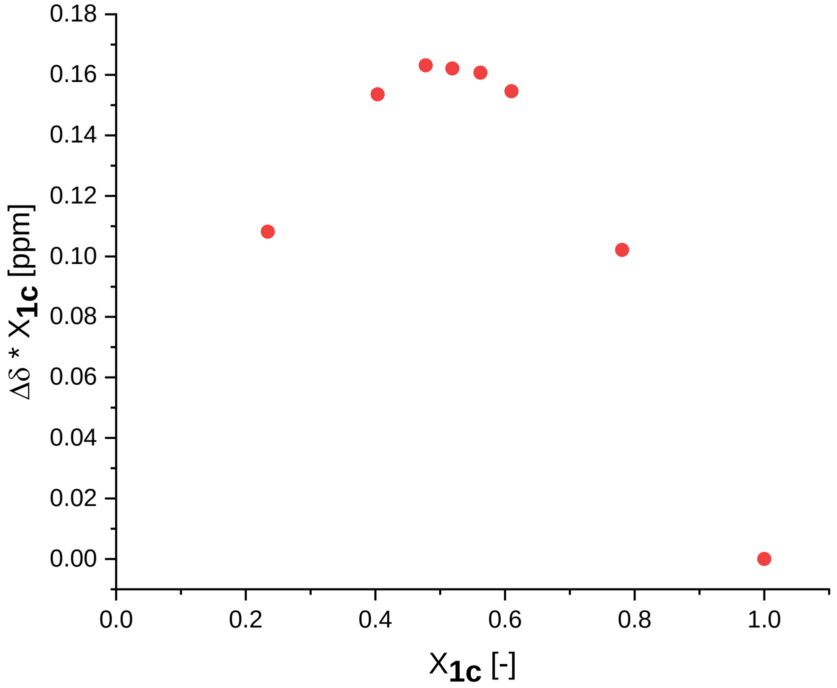

Figure S27. Job's plot from the chemical shift change |Δδ| of the protons of **1c** in ^{1}H NMR spectra by varying the ratio between **1c** and *n*-$Bu_4N^+F^-$.

2. Synthesis of New Compounds

General remarks. All chemicals were reagent grade and used without further purification. Reactions were monitored by TLC analysis with 0.20 mm *Machevery-Nagel* POLYGRAM SIL G/UV254 with fluorescent indicator UV254. Flash chromatography was carried out on silica 60 (40-63 μm, 230-400 mesh). ^{1}H, ^{13}C, ^{19}F and ^{125}Te NMR spectra were measured with Bruker Avance NEO 400 and Avance HD 600 spectrometers. All chemical shifts (δ) are given in ppm. The spectra were referenced to the peak for the protium impurity in the deuterated solvents indicated in brackets in the analytical data. Signal multiplicity for ^{1}H, ^{19}F and ^{13}C NMR was determined as s (singlet), d (doublet), t (triplet), sext (sextet), sept (septet), m (multiplet), dd (doublet of doublets), td (triplet of doublets) and br (broad signal). ^{13}C NMR spectra were measured with ^{1}H decoupling and the ^{13}C assignment was achieved via DEPT 135, HSQC, HMBC, and COSY spectra. ^{13}C signal degree of substitution was determined as p (primary), s (secondary), t (tertiary), q (quaternary). HR-MS spectra were recorded with a Bruker BioTOF III Instrument with ESI as ionization source. UV/Vis absorption spectra were obtained with a Jasco V-550 spectrophotometer. The infrared spectra were recorded with the Shimadzu IR Tracer 100 and the characteristic wave numbers are given in cm^{-1}.

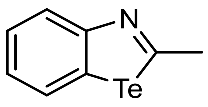

2

Synthesis of benzotellurazole 2. To a solution of ditelluride **3** (1.32 g, 3.0 mmol) in THF (24 mL), acid chloride **4** (490 mg, 6.1 mmol) was added and the mixture was stirred for 5 min. Then, 50% aq. hypophosphorous acid (0.6 mL) was added and the solution was stirred for 5 min at room temperature. This step was followed by an addition of 95% ethanol (9.6 ml) and 36% hydrochloric acid (6 mL). The resulting mixture was heated to reflux for 1–3 hours. After cooling to room temperature, the mixture was chilled in an ice-bath for 30 min. Ammonia solution (5% aq.) was added to the cooled mixture to basify to pH = 8 and the solution was repeatedly extracted with CH_2Cl_2. The organic layers were combined, dried over $MgSO_4$, and the solvent was removed in vacuo. The residue was purified by chromatography on silica gel (*n*-pentane/EtOAc 6:1) to yield benzotellurazole **2** as a yellow solid (860 mg, 3.51 mmol, 56%). M.p.: 93–95 °C. ^{1}H NMR (400 MHz, $CDCl_3$): δ = 8.06 (dd, $^{3}J_{H,H}$ = 8.0, $^{4}J_{H,H}$ = 1.2 Hz, 1 H; C_{ar}H), 7.86–7.84 (m, 2 H; C_{ar}H, C_{ar}H),

7.15 (td, ${}^3J_{H,H}$ = 7.6, ${}^4J_{H,H}$ = 1.2 Hz, 1 H; $C_{ar}H$), 2.86 ppm (s, 1 H; CH_3). ${}^{13}C$ NMR (101 MHz, $CDCl_3$): δ = 170.5 (q; C_{ar}), 160.7 (q; C_{ar}), 135.4 (q; C_{ar}), 131.8 (t; C_{ar}), 126.9 (t; C_{ar}), 125.6 (t; C_{ar}), 124.8 (t; C_{ar}), 30.9 ppm (p; CH_3). ${}^{125}Te$ NMR (126 MHz, $CDCl_3$): δ = 913.5 ppm. IR (ATR): $\tilde{\nu}$ = 3046, 1582, 1422, 1288, 1130, 980, 843, 760, 635 cm^{-1}. UV/Vis (CH_3CN): λ_{max} (log ε) = 238 nm (4.09). HRMS (ESI-TOF) *m/z*: $[C_8H_7N^{130}Te+H]^+$ calculated: 247.9717; found: 247.9721.

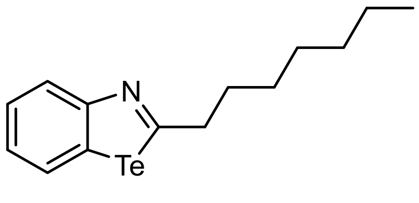

6

Synthesis of benzotellurazole 6. To a solution of ditelluride **3** (351 mg, 0.8 mmol) in THF (5 mL), acid chloride **5** (276 mg, 1.7 mmol) was added and the mixture was stirred for 5 min. Then, 50% aq. hypophosphorous acid (0.16 mL) was added and the solution was stirred for 5 min at room temperature. This step was followed by an addition of 95% ethanol (2.5 mL) and 36% hydrochloric acid (1.6 mL). The resulting mixture was heated to reflux for 1–3 hours. After cooling to room temperature, the mixture was chilled in an ice-bath for 30 min. Ammonia solution (5% aq.) was added to the cooled mixture to basify to pH = 8 and the solution was repeatedly extracted with CH_2Cl_2. The organic layers were combined, dried over $MgSO_4$, and the solvent was removed in vacuo. The residue was purified by chromatography on silica gel (hexanes/EtOAc 3:1) to yield benzotellurazole **6** as a yellow solid (206 mg, 0.63 mmol, 78%). M.p.: 50–51 °C. ${}^1H$ NMR (400 MHz, $CDCl_3$): δ = 8.06 (dd, ${}^3J_{H,H}$ = 8.1, ${}^4J_{H,H}$ = 1.1 Hz, 1 H; $C_{ar}H$), 7.88 (dd, ${}^3J_{H,H}$ = 7.8, ${}^4J_{H,H}$ = 1.0 Hz, 1 H; $C_{ar}H$), 7.46–7.42 (m, 1 H; $C_{ar}H$), 7.16–7.12 (m, 1 H; $C_{ar}H$), 3.04 (t, ${}^3J_{H,H}$ = 7.6, 2 H; CH_2), 1.85–1.77 (m, 2 H; CH_2), 1.51–1.43 (m 2 H; CH_2), 1.39–1.25 (m, 6 H; CH_2), 0.88 ppm (t, ${}^3J_{H,H}$ = 7.0, 3 H; CH_3). ${}^{13}C$ NMR (101 MHz, $CDCl_3$): δ = 178.8 (q; C_{ar}), 160.6 (q; C_{ar}), 134.1 (q; C_{ar}), 131.7 (t; C_{ar}), 126.7 (t; C_{ar}), 125.6 (t; C_{ar}), 124.5 (t; C_{ar}), 43.5 (s; CH_2), 31.6 (p; CH_3), 31.3 (p; CH_3), 29.1 (p; CH_3), 29.0 (p; CH_3), 22.6 (p; CH_3), 14.1 ppm (p; CH_3). ${}^{125}Te$ NMR (126 MHz, $CDCl_3$): δ = 876.9 ppm. IR (ATR): $\tilde{\nu}$ = 3352, 3046, 2918, 1582, 1516, 1464, 1439, 1290, 1130, 1016, 847, 768, 716, 658, 650 cm^{-1}. UV/Vis (CH_3CN): λ_{max} (log ε) = 312 (3.41), 239 nm (4.26). HRMS (ESI-TOF) *m/z*: $[C_{14}H_{19}N^{130}Te+H]^+$ calculated: 332.0653; found: 332.0654.

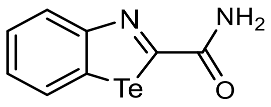

8a

Synthesis of benzotellurazole 8a. To a solution of benzotellurazole **7a** (1.15 g, 3.80 mmol) in ethanol (40 mL), ammonia solution (16 mL, 25% aq.) was added, and the mixture was subsequently allowed to heat to reflux for 5 hours. After cooling to room temperature, ethanol was removed under vacuo, the residue was repeatedly extracted with CH_2Cl_2 (2×30 mL). The organic layers were combined, dried with $MgSO_4$, and concentrated in vacuo to provide benzotellurazole **8a** as a yellow solid (766 mg, 2.8 mmol, 74%). M.p.: 172–174 °C. ^{1}H NMR (400 MHz, $CDCl_3$): δ = 8.24 (dd, $^3J_{H,H}$ = 8.0, $^4J_{H,H}$ = 0.8 Hz, 1 H; C_{ar}H), 8.01–7.99 (m, 1 H; C_{ar}H), 7.55–7.14 (m, 1 H; C_{ar}H), 7.15 (br.s, 1 H; NH), 5.73 ppm (br.s, 1 H; NH). ^{13}C NMR (101 MHz, $CDCl_3$): δ = 172.0 (q; C_{ar}), 166.5 (q; CO), 161.6 (q; C_{ar}), 137.8 (q; C_{ar}), 132.4 (t; C_{ar}), 128.4 (t; C_{ar}), 127.3 (t; C_{ar}), 126.3 ppm (t; C_{ar}). ^{125}Te NMR (126 MHz, $CDCl_3$): δ = 932.4 ppm. IR (ATR): $\tilde{\nu}$ = 3181, 1668, 1643, 1497, 1292, 1051, 854, 770, 662 cm^{-1}. UV/Vis (MeOH): λ_{max} (log ε) = 244 nm (3.50). HRMS (ESI-TOF) *m*/*z*: $[C_8H_6N_2O^{130}Te+H]^+$ calculated: 276.9615; found: 276.9507.

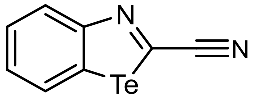

1a

Synthesis of benzotellurazole 1a. Benzotellurazole **8a** (710 mg, 2.6 mmol) was dissolved in anhydrous pyridine (28 mL) and stirred under argon at 0° C, followed by dropwise addition of $POCl_3$ (6 mL). After 30 minutes, the ice bath was removed and the reaction was stirred overnight at room temperature. The reaction was then quenched by adding cold water dropwise, after which the solution was extracted with CH_2Cl_2. The aqueous layer was saturated with NaCl and repeatedly re-extracted with CH_2Cl_2 (2×40 mL). The organic layers were combined, dried over $MgSO_4$ and concentrated under vacuum. The residue was then purified using flash column chromatography with silica gel (*n*-pentane/EtOAc, 9:1) to yield benzotellurazole **1a** as a yellow solid (450 mg, 1.76 mmol, 68%). M.p.: 170–173 °C. ^{1}H NMR (400 MHz, $CDCl_3$): δ = 8.46–8.44 (m, 1 H; C_{ar}H), 8.00 (dd, $^3J_{H,H}$ = 8.0 Hz, $^4J_{H,H}$ = 0.8 Hz, 1 H; C_{ar}H), 7.64–7.60 (m, 1 H; C_{ar}H), 7.42–7.38 ppm (m, 1 H; C_{ar}H). ^{13}C NMR (101 MHz, $CDCl_3$): δ = (q; C_{ar}), 138.0 (q; C_{ar}), 136.7 (q; C_{ar}), 131.6 (t; C_{ar}), 129.3

(t; C_{ar}), 128.3 (t; C_{ar}), 128.0 (t; C_{ar}), 120.1 ppm (q; CN). ^{125}Te NMR (126 MHz, $CDCl_3$): δ = 1140.2 ppm. IR (ATR): $\tilde{\nu}$ = 2920, 2220, 1582, 1456, 1300, 1086, 760, 654 cm^{-1}. UV/Vis (CH_3CN): λ_{max} (log ε) = 296 (3.87), 244 nm (4.04). HRMS (ESI-TOF) *m*/*z*: $[C_8H_4N_2{}^{130}Te+H]^+$ calculated: 258.9509; found: 258.9509.

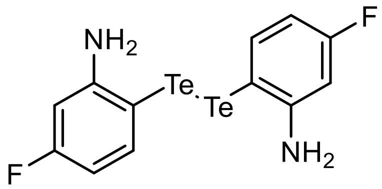

10a

Synthesis of ditelluride 10a. A suspension of NaH (60% dispersion in mineral oil, 6.6 g, 164.6 mmol) was added to tellurium (powder, 7.0 g, 54.9 mmol) in anhydrous NMP under an argon atmosphere. After stirring at 185 °C for 1h, 2-bromo-5-fluoroaniline (10.4 g, 54.9 mmol) was added to the suspension. Stirring was continued at 185 °C for 4 h. It was then allowed to cool to room temperature, diluted with water (100 mL), and buffered by the addition of NH_4Cl (8.9 g). The mixture was stirred overnight. Afterwards, the aqueous phase was repeatedly extracted with Et_2O until the organic phase became slightly yellow (4×100 mL). The combined organic extracts were washed with H_2O (3×100 mL), dried over $MgSO_4$, filtered and the solvents concentrated in vacuo. The crude product was purified by crystallization from hot hexanes to yield ditelluride **10a** as an orange solid (3.85 g, 8.1 mmol, 30%). M.p.: 115–116 °C. ^{1}H NMR (400 MHz, $CDCl_3$): δ = 7.57 (dd, $^3J_{H,H}$ = 8.5, $^4J_{H,F}$ = 6.8 Hz, 2 H; C_{ar}H), 6.43 (dd, $^3J_{H,F}$ = 10.9, $^4J_{H,H}$ = 2.5 Hz, 2 H; C_{ar}H), 6.25 (td, $^3J_{H,H}$ = 8.5, $^4J_{H,H}$ = 2.6 Hz, 2 H; C_{ar}H), 4.32 ppm (s, 4 H; NH). ^{13}C NMR (101 MHz, $CDCl_3$): δ = 165.7 (d, $^1J_{C,F}$ = 247.1 Hz, q; C_{ar}F), 152.5 (q; C_{ar}), 144.9 (d, $^3J_{C,F}$ = 9.8 Hz, t; C_{ar}), 106.2 (d, $^2J_{C,F}$ = 21.4 Hz, t; C_{ar}), 100.1 (d, $^2J_{C,F}$ = 24.5 Hz, t; C_{ar}), 88.6 ppm (q; C_{ar}). ^{125}Te NMR (126 MHz, $CDCl_3$): δ = 274.8 ppm. ^{19}F NMR (151 MHz, $CDCl_3$): δ = -109.17 ppm (m). IR (ATR): $\tilde{\nu}$ = 3373, 3283, 3171, 2922, 1609, 1566, 1474, 1416, 1279, 1246, 1165, 1119, 1076, 1020, 970, 830, 779, 739, 710 cm^{-1}. UV/Vis (CH_3CN): λ_{max} (log ε) = 338 (3.70), 229 nm (4.49). HRMS (ESI-TOF) *m*/*z*: $[C_{12}H_{10}F_2N_2{}^{130}Te_2+H]^+$ calculated: 476.8979; found: 476.7971.

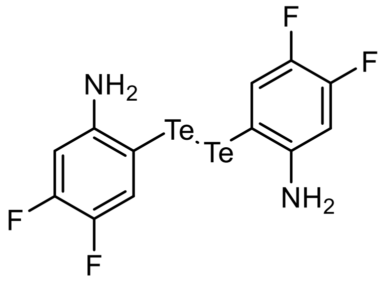

10b

Synthesis of ditelluride 10b. A suspension of NaH (60% dispersion in mineral oil, 6.01 g, 156.7 mmol) was added to the tellurium (Powder, 7.49 g, 58.8 mmol) in anhydrous NMP under an argon atmosphere. After stirring at 185 °C for 1h, 2-bromo-4,5-difluoroaniline (8.15 g, 39.2 mmol) was added to the suspension. Stirring continued at 185 °C for 4 h. It was then allowed to cool to room temperature, and diluted with water (100 mL), and buffered by the addition of NH_4Cl (8.9 g). The mixture was stirred overnight. Afterwards, the aqueous phase was repeatedly extracted with Et_2O until the organic phase became slightly yellow (4x 100 mL). The combined organic extracts were washed with H_2O (3x 100 mL), dried over $MgSO_4$, filtered and the solvents concentrated in vacuo. The crude product was purified by crystallization from hot hexanes to yield ditelluride **10b** as an orange solid (2.37 g, 4.6 mmol, 24%). M.p.: 75 °C. ^{1}H NMR (400 MHz, $CDCl_3$): δ = 7.44 (t, $^3J_{H,F}$ = 9.3 Hz, 2 H; C_{ar}H), 6.54 (dd, $^3J_{H,F}$ = 12.0, $^4J_{H,F}$ = 6.7 Hz, 2 H; C_{ar}H), 4.14 (s, 4 H; NH).^{13}C NMR (101 MHz, $CDCl_3$): δ = 152.65 (dd, $^1J_{C,F}$ = 250.5 Hz, $^2J_{C,F}$ = 13.7 Hz, q; C_{ar}), 147.69 (q; C_{ar}), 142.61 (dd, $^1J_{C,F}$ = 243.3 Hz, $^2J_{C,F}$ = 12.9 Hz, q; C_{ar}), 130.51 (d, $^2J_{C,F}$ = 17.4 Hz, t; C_{ar}), 101.60 (d, $^2J_{C,F}$ = 20.6 Hz, t; C_{ar}), 86.88 (q; C_{ar}). ^{125}Te NMR (126 MHz, $CDCl_3$): δ = 274.8 ppm. ^{19}F NMR (151 MHz, $CDCl_3$): δ = -133.15 (m), -150.58 ppm (m). IR (ATR): ṽ = 3356, 3267, 3163, 3046, 1614, 1585, 1509, 1404, 1323, 1285, 1194, 1159, 986, 880, 841, 802, 758, 718, 677, 642 cm^{-1}. UV/Vis (CH_3CN): λ_{max} (log ε) = 345 nm (4.05).

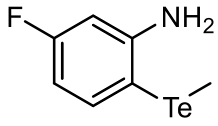

11a

Synthesis of aniline 11a. To a solution of **10a** (2.85 g, 6.0 mmol) in dry THF (120 mL) under argon atmosphere $NaBH_4$ (698 mg, 18.0 mmol) and MeOH (1.2 mL, 961 mg, 30 mmol) were added. The solution was stirred for an hour, until MeI (822 µL, 1.91 g, 13.2 mmol) was added.

Afterwards the solution continued to stir until TLC showed the consumption of the starting material (2 h). Then 20 mL water were added, and the aqueous phase was extracted with Et_2O (3 x 30 mL). The combined organic extracts were washed with H_2O (10 mL) and brine (10 mL), dried over $MgSO_4$, filtered and the solvents removed under vacuum. The crude was purified by flash column chromatography with silica gel (hexanes/CH_2Cl_2, 1:3) to obtain **11a** as a light brown liquid. (2.68 g, 10.6 mmol, 88%). ^{1}H NMR (400 MHz, $CDCl_3$): δ = 7.68 (dd, $^3J_{H,H}$ = 8.4, $^4J_{H,F}$ = 6.8 Hz, 1 H; C_{ar}H), 6.46 (dd, $^3J_{H,F}$ = 10.9 Hz, $^4J_{H,H}$ = 2.6 Hz, 1 H; C_{ar}H), 6.30 (td, $^3J_{H,F}$ = 8.5, $^4J_{H,H}$ = 2.6 Hz, 1 H; C_{ar}H),4.45 (s, 2 H; NH), 2.00 ppm (s, 3 H; CH_3). ^{13}C NMR (101 MHz, $CDCl_3$): δ = 165.0 (d, $^1J_{C,F}$ = 245.0 Hz, q; C_{ar}), 151.6 (d, $^3J_{C,F}$ = 10.9 Hz, q; C_{ar}), 143.6 (d, $^3J_{C,F}$ = 9.4 Hz, t; C_{ar}), 106.2 (d, $^2J_{C,F}$ = 20.9 Hz, t; C_{ar}), 100.1 (d, $^2J_{C,F}$ = 24.5 Hz, t; C_{ar}), 93.2 (d, $^4J_{C,F}$ = 2.5 Hz, q; C_{ar}), -16.6 ppm (p; CH_3). ^{125}Te NMR (126 MHz, $CDCl_3$): δ = 126.5 ppm. ^{19}F NMR (151 MHz, $CDCl_3$): δ = -111.7 ppm (m). IR (ATR): $\tilde{\nu}$ = 3445, 3343, 2926, 1568, 1476, 1422, 1279, 1254, 1217, 1150, 1115, 970, 783, 617 cm^{-1}. UV/Vis (CH_3CN): λ_{max} (log ε) = 300 nm (3.48). HRMS (ESI-TOF) *m*/*z*: $[C_7H_8FN^{130}Te+H]^+$ calculated: 255.9776; found: 255.9773.

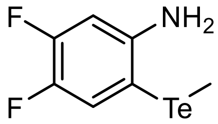

11b

Synthesis of aniline 11b. To a solution of **10b** (2.0 g, 3.9 mmol) in dry THF (100 mL) under argon atmosphere $NaBH_4$ (444 mg, 11.7 mmol) and MeOH (793 µL, 626 mg, 19.6 mmol) were added. The solution was stirred for an hour, until MeI (609 µL, 1.39 g, 9.8 mmol) was added. Afterwards the solution continued to stir until TLC showed the consumption of the starting material (2 h). Then 20 mL water were added, and the aqueous phase was extracted with Et_2O (3×30 mL). The combined organic extracts were washed with H_2O (10 mL) and brine (10 mL), dried over $MgSO_4$, filtered and the solvents removed under vacuum. The crude was purified by flash column chromatography with silica gel (hexanes/CH_2Cl_2, 1:3) to obtain **11b** as a light brown liquid (1.86 g, 6.9 mmol, 88%). ^{1}H NMR (400 MHz, $CDCl_3$): δ = 7.50 (t, $^3J_{H,F}$ = 9.4 Hz, 1 H; C_{ar}H), 6.55 (dd, $^3J_{H,F}$ = 12.1, $^4J_{H,F}$ = 6.8 Hz, 1 H; C_{ar}H), 4.22 (s, 2 H; NH), 2.04 ppm (s, 3 H; CH_3). ^{13}C NMR (101 MHz, $CDCl_3$): δ = 151.7 (dd, $^1J_{C,F}$ = 248.2 Hz, $^2J_{C,F}$ = 13.1 Hz, q; C_{ar}), 146.7 (dd, $^3J_{C,F}$ = 8.9 Hz, $^4J_{C,F}$ = 2.2 Hz, q; C_{ar}), 142.9 (dd, $^1J_{C,F}$ = 241. Hz, $^2J_{C,F}$ = 12.5 Hz, q; C_{ar}), 129.2 (t; C_{ar}), 100.6 (d,

$^{2}J_{C,F}$ = 20.0 Hz, t; C_{ar}), 91.9 (t, $^{3}J_{C,F}$ = 3.6 Hz, q; C_{ar}), -16.2 ppm (p; CH_3). ^{125}Te NMR (126 MHz, $CDCl_3$): δ = 169.6 ppm. ^{19}F NMR (151 MHz, $CDCl_3$): δ = -135.6 (m), -151.3 ppm (m). IR (ATR): ṽ = 3345, 3343, 2928, 1612, 1587, 1408, 1317, 1285, 1233, 1221, 1196, 1159, 989, 874, 833, 797, 617 cm^{-1}. UV/Vis (CH_3CN): λ_{max} (log ε) = 309 nm (3.61). HRMS (ESI-TOF) *m*/*z*: $[C_7H_7F_2N^{130}Te+H]^+$ calculated: 273.9682; found: 273.9682.

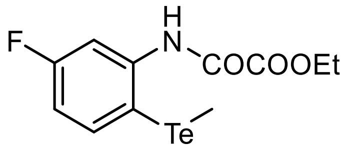

12a

Synthesis of aniline 12a. To a solution of **11a** (2.28 g, 9.0 mmol) in dry CH_2Cl_2 (25 mL), ethyl chlorooxoacetat (1.35 g, 9.9 mmol) and Et_3N (1.5 mL, 1.09 g, 10.8 mmol) were added. The reaction was stirred at room temperature until TLC showed the consumption of the starting material (2 h). The mixture was diluted with CH_2Cl_2 (50 mL), washed with H_2O (10 mL) and brine (10 mL), dried over $MgSO_4$, filtered and the solvents removed under vacuum. The crude was purified by flash column chromatography with silica gel (hexanes/EtOAc, 3:1) to obtain **12a** as a white solid. (2.54 g, 7.2 mmol, 80%). M.p.: 99–100 °C. ^{1}H NMR (400 MHz, $CDCl_3$): δ = 10.08 (s, 1 H; NH), 8.27 (dd, $^{3}J_{H,F}$ = 11.1, $^{4}J_{H,H}$ = 2.7 Hz, 1 H; C_{ar}H), 7.92 (dd, $^{3}J_{H,H}$ = 8.5 Hz, $^{4}J_{H,F}$ = 6.5 Hz, 1 H; C_{ar}H), 6.76 (td, $^{3}J_{H,F}$ = 8.2, $^{4}J_{H,H}$ = 2.8 Hz, 1 H; C_{ar}H), 4.45 (q, $^{3}J_{H,H}$ = 7.1 Hz, 2 H; CH_2), 2.06 (s, 3 H; CH_3), 1.45 ppm (t, $^{3}J_{H,H}$ = 7.1 Hz, 3 H; CH_3). ^{13}C NMR (101 MHz, $CDCl_3$): δ = 165.3–162.9 (d, $^{1}J_{C,F}$ = 248.0 Hz, q; C_{ar}), 160.4 (q; CO), 154.2 (q; CO), 143.4 (d, $^{3}J_{C,F}$ = 8.7 Hz, t; C_{ar}), 141.2 (d, $^{3}J_{C,F}$ = 11.8 Hz, q; C_{ar}), 113.2 (d, $^{2}J_{C,F}$ = 21.2 Hz, t; C_{ar}), 107.3 (d, $^{2}J_{C,F}$ = 27.7 Hz, t; C_{ar}), 99.5 (d, $^{4}J_{C,F}$ = 3.6 Hz, q; C_{ar}), 63.9 (s; CH_2), 14.0 (p; CH_3) -14.5 ppm (p; CH_3). ^{125}Te NMR (126 MHz, $CDCl_3$): δ = 154.8 ppm. ^{19}F NMR (151 MHz, $CDCl_3$): δ = -107.9 ppm (s). IR (ATR): ṽ = 3265, 3100, 2982, 1713, 1580, 1441, 1414, 1369, 1306, 1277, 1223, 1186, 1144, 1009, 976, 868, 808, 617, 519 cm^{-1}. UV/Vis (CH_3CN): λ_{max} (log ε) = 271 nm (4.32). HRMS (ESI-TOF) *m*/*z*: $[C_{11}H_{12}FNO_3{}^{130}Te+H]^+$ calculated: 355.9937; found: 355.9934.

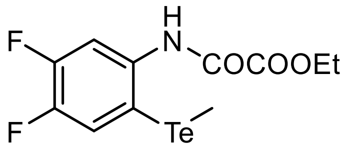

12b

Synthesis of aniline 12b. To a solution of **11b** (1.62 g, 6.0 mmol) in dry CH_2Cl_2 (25 mL), ethyl chlorooxoacetat (983 mg, 7.2 mmol) and Et_3N (1.0 mL, 729 mg, 7.2 mmol) were added. The reaction was stirred at room temperature until TLC showed the consumption of the starting material (2 h). The mixture was diluted with CH_2Cl_2 (50 mL), washed with H_2O (10 mL) and brine (10 mL), dried over $MgSO_4$, filtered and the solvents removed under vacuum. The crude was purified by flash column chromatography with silica gel (CH_2Cl_2) to obtain **12b** as a white solid (1.99 g, 5.4 mmol, 90%). M.p.: 105 °C. ^{1}H NMR (400 MHz, $CDCl_3$): δ = 9.91 (s, 1 H; NH), 8.38 (dd, $^3J_{H,F}$ = 12.5, $^4J_{H,F}$ = 7.5 Hz, 1 H; C_{ar}H), 7.73 (t, $^4J_{H,F}$ = 8.9 Hz, 1 H; C_{ar}H), 4.45 (q, $^3J_{H,H}$ = 7.2 Hz, 2 H; CH_2), 2.11 (s, 3 H; CH_3), 1.45 ppm (t, $^3J_{H,H}$ = 7.2 Hz, 3 H; CH_3). ^{13}C NMR (101 MHz, $CDCl_3$): δ = 160.5 (q; CO), 154.1 (q; CO), 152.3–149.7 (dd, $^1J_{C,F}$ = 250.4 Hz, $^2J_{C,F}$ = 13.2 Hz, q; C_{ar}), 148.1–145.5 (dd, $^1J_{C,F}$ = 251.9 Hz, $^2J_{C,F}$ = 12.9 Hz, q; C_{ar}), 145.6 (d, $^2J_{C,F}$ = 12.8 Hz, q; C_{ar}), 136.3 (dd $^3J_{C,F}$ = 9.7 Hz, $^4J_{C,F}$ = 3.2 Hz, t; C_{ar}), 129.6 (d, $^2J_{C,F}$ = 17.3 Hz, t; C_{ar}), 109.0 (d, $^2J_{C,F}$ = 23.3 Hz, t; C_{ar}), 99.1 (t, $^3J_{C,F}$ = 3.9 Hz, q; C_{ar}), 64.0 (s; CH_2), 14.0 (p; CH_3), -14.1 ppm (p; CH_3). ^{125}Te NMR (126 MHz, $CDCl_3$): δ = 196.3 ppm. ^{19}F NMR (151 MHz, $CDCl_3$): δ = -131.9 (m), -139.8 ppm (m). IR (ATR): $\tilde{\nu}$ = 3221, 2924, 1730, 1701, 1597, 1524, 1458, 1402, 1373, 1306, 1294, 1252, 1217, 1196, 1179, 1016, 880, 845, 733, 513 cm^{-1}. UV/Vis (CH_3CN): λ_{max} (log ε) = 273 nm (3.89). HRMS (ESI-TOF) *m*/*z*: $[C_{11}H_{11}F_2NO_3{}^{130}Te+H]^+$ calculated: 373.9842; found: 373.9841, $[C_{11}H_{11}F_2NO_3{}^{130}Te+Na]^+$ calculated: 395.9662; found: 395.9661.

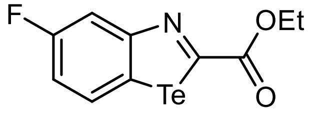

7b

Synthesis of aniline 7b. A solution of **12a** (1.04 g, 3.0 mmol) and Et_3N (2.5 mL, 1.79 g, 17.7 mmol) was prepared under anhydrous conditions in dry 1,4-dioxane (20 mL). Then $POCl_3$ (540 µL, 905 mg, 5.9 mmol) was added dropwise under stirring and the reaction was heated to reflux for 3 h. The mixture was diluted with a saturated solution of $NaHCO_3$ (10 mL) and then extracted

with CH_2Cl_2 (3×30 mL). The organic layers were combined, dried over $MgSO_4$ and concentrated under vacuum. The residue was then purified using flash column chromatography with silica gel (CH_2Cl_2) to yield benzotellurazole **7b** as a yellow solid (743 mg, 2.3 mmol, 79%). M.p.: 112–114 °C. ^{1}H NMR (400 MHz, $CDCl_3$): δ = 8.15 (dd, $^{3}J_{H,F}$ = 9.8, $^{4}J_{H,H}$ = 2.6 Hz, 1 H; $C_{ar}H$), 7.92 (dd, $^{3}J_{H,H}$ = 8.7 Hz, $^{4}J_{H,F}$ = 5.6 Hz, 1 H; $C_{ar}H$), 7.12 (td, $^{3}J_{H,F}$ = 8.6, $^{4}J_{H,H}$ = 2.6 Hz, 1 H; $C_{ar}H$), 4.51 (q, $^{3}J_{H,H}$ = 7.1 Hz, 2 H; CH_2), 1.47 ppm (t, $^{3}J_{H,H}$ = 7.1 Hz, 3 H; CH_3). ^{13}C NMR (101 MHz, $CDCl_3$): δ = 168.5 (q; C_{ar}), 165.4 (q; CO), 164.0–161.6 (d, $^{1}J_{C,F}$ = 245.0 Hz, q; C_{ar}), 162.5 (d, $^{3}J_{C,F}$ = 11.8 Hz, q; C_{ar}), 132.4 (d, $^{3}J_{C,F}$ = 9.0 Hz, t; C_{ar}), 131.9 (d, $^{4}J_{C,F}$ = 2.4 Hz, q; C_{ar}), 115.6 (d, $^{2}J_{C,F}$ = 23.7 Hz, t; C_{ar}), 115.2 (d, $^{2}J_{C,F}$ = 22.6 Hz, t; C_{ar}), 63.5 (s; CH_2), 14.0 ppm (p; CH_3). ^{125}Te NMR (126 MHz, $CDCl_3$): δ = 992.0 ppm. ^{19}F NMR (151 MHz, $CDCl_3$): δ = -115.1 ppm (m). IR (ATR): ṽ = 2982, 1713, 1582, 1553, 1489, 1366, 1246, 1221, 1055, 1028, 961, 868, 816, 748, 712, 652, 619 cm^{-1}. UV/Vis (CH_3CN): λ_{max} (log ε) = 377 (3.58), 292 nm (4.03). HRMS (ESI-TOF) *m*/*z*: $[C_{10}H_8FNO_2{}^{130}Te+H]^+$ calculated: 323.9674; found: 323.9674, $[C_{10}H_8FNO_2{}^{130}Te+Na]^+$ calculated: 345.9494; found: 345.9494.

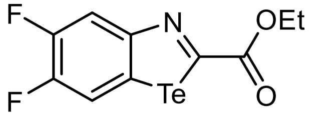

7c

Synthesis of aniline 7c. A solution of **12b** (1.87 g, 5.1 mmol) and Et_3N (4.2 mL, 3.06 g, 30.3 mmol) was prepared under anhydrous conditions in dry 1,4-dioxane (30 mL). Then $POCl_3$ (920 µL, 1.55 g, 10.1 mmol) was added dropwise under stirring and the reaction was heated to reflux for 3 h. The mixture was diluted with a saturated solution of $NaHCO_3$ (10 mL) and then extracted with CH_2Cl_2 (3×30 mL). The organic layers were combined, dried over $MgSO_4$ and concentrated under vacuum. The residue was then purified using flash column chromatography with silica gel (CH_2Cl_2) to yield benzotellurazole **7c** as a yellow solid (1.51 g, 4.5 mmol, 88%). M.p.: 179–180 °C. ^{1}H NMR (400 MHz, $CDCl_3$): δ = 8.21 (dd, $^{3}J_{H,F}$ = 10.9, $^{4}J_{H,F}$ = 7.2 Hz, 1 H; $C_{ar}H$) 7.70 (dd, $^{3}J_{H,F}$ = 9.0, $^{4}J_{H,F}$ = 8.0 Hz, 1 H; $C_{ar}H$), 4.51 (q, $^{3}J_{H,H}$ = 7.1 Hz, 2 H; CH_2), 1.47 ppm (t, $^{3}J_{H,H}$ = 7.1 Hz, 3 H; CH_3).^{13}C NMR (101 MHz, $CDCl_3$): δ = 167.4 (d, $^{4}J_{C,F}$ = 3.3 Hz q; C_{ar}), 165.3 (q; CO), 157.9 (dd, $^{3}J_{C,F}$ = 9.3 Hz, $^{4}J_{C,F}$ = 2.7 Hz, q; C_{ar}), 152.2–149.6 (dd, $^{1}J_{C,F}$ = 248.7 Hz, $^{2}J_{C,F}$ = 14.6 Hz, q; C_{ar}), 150.8–148.1 (dd, $^{1}J_{C,F}$ = 255.9 Hz, $^{2}J_{C,F}$ = 14.3 Hz, q; C_{ar}), 131.9 (m, q; C_{ar}), 118.9 (d, $^{2}J_{C,F}$ = 19.9 Hz, t; C_{ar}), 116.5 (d, $^{2}J_{C,F}$ = 18.3 Hz, t; C_{ar}),63.6 (s; CH_2), 14.3 ppm (p; CH_3). ^{125}Te NMR

(126 MHz, $CDCl_3$): δ = 1029.7 ppm. ^{19}F NMR (151 MHz, $CDCl_3$): δ = -135.6 (m), -136.8 ppm (m). IR (ATR): ṽ = 3059, 3003, 1717, 1566, 1497, 1456, 1447, 1368, 1288, 1250, 1126, 1097, 1049, 1011, 978, 903, 858, 800, 770, 752, 652 cm^{-1}. UV/Vis (CH_3CN): λ_{max} (log ε) = 371 (3.41), 295 (3.90) 247 nm (3.98). HRMS (ESI-TOF) *m*/*z*: $[C_{10}H_7F_2NO_2{}^{130}Te+H]^+$ calculated: 341.9580; found: 341.9578, $[C_{10}H_7F_2NO_2{}^{130}Te+Na]^+$ calculated: 363.9400; found: 363.9402.

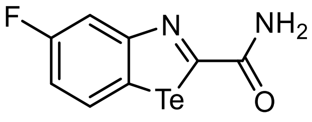

8b

Synthesis of benzotellurazole 8b. To a solution of benzotellurazole **7b** (320 mg, 1.0 mmol) in ethanol (10 mL), ammonia solution (3 mL, 25% aq.) was added, and the mixture was subsequently allowed to heat to reflux for 5 hours. After cooling to room temperature, ethanol was removed under vacuo, the residue was repeatedly extracted with CH_2Cl_2 (2×30 mL). The organic layers were combined, dried with $MgSO_4$, and concentrated in vacuo to provide benzotellurazole **8b** as a yellow solid (185 mg, 0.6 mmol, 63%). M.p.: 178–180 °C. ^1H NMR (400 MHz, $CDCl_3$): δ = 7.95–7.89 (m, 2 H; $C_{ar}H$), 7.11–7.06 (m, 2 H; $C_{ar}H$, NH), 5.56 ppm (br.s, 1 H; NH). ^{13}C NMR (101 MHz, $CDCl_3$): δ = 174.8 (q; CO), 165.9 (q; C_{ar}), 163.9–161.2 (d, $^1J_{C,F}$ = 244.9 Hz, q; C_{ar}), 162.4 (d, $^3J_{C,F}$ = 10.4 Hz, q; C_{ar}), 132.8 (d, $^3J_{C,F}$ = 8.8 Hz, t; C_{ar}), 131.9 (d, $^4J_{C,F}$ = 2.2 Hz, q; C_{ar}), 114.9 (d, $^2J_{C,F}$ = 23.2 Hz, t; C_{ar}), 114.2 ppm (d, $^2J_{C,F}$ = 22.1 Hz, t; C_{ar}). ^{125}Te NMR (126 MHz, $CDCl_3$): δ = 947.5 ppm. ^{19}F NMR (151 MHz, $CDCl_3$): δ = -115.6 ppm (m). IR (ATR): ṽ = 3458, 3152, 1684, 1655, 1601, 1557, 1503, 1443, 1389, 1292, 1258, 1142, 1111, 1053, 1015, 959, 862, 800, 764, 677, 590 cm^{-1}. UV/Vis (CH_3CN): λ_{max} (log ε) = 371 (3.55), 288 (3.96), 240 nm (4.14). HRMS (ESI-TOF) *m*/*z*: $[C_8H_5FN_2O^{130}Te+H]^+$ calculated: 294.9521; found: 294.9520, $[C_8H_5FN_2O^{130}Te+Na]^+$ calculated: 316.9341; found: 316.9339.

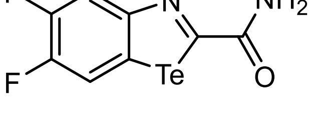

8c

Synthesis of benzotellurazole 8c. To a solution of benzotellurazole **7c** (1.25 g, 3.7 mmol) in ethanol (40 mL), ammonia solution (16 mL, 25% aq.) was added, and the mixture was subsequently allowed to heat to reflux for 5 hours. After cooling to room temperature, ethanol was removed under vacuo, the residue was repeatedly extracted with CH_2Cl_2 (2×30 mL). The organic layers were combined, dried with $MgSO_4$, and concentrated in vacuo to provide benzotellurazole **8c** as a yellow solid (730 mg, 2.4 mmol, 64%). M.p.: 175–177 °C. ^{1}H NMR (400 MHz, $CDCl_3$): δ = 8.0 (dd, $^3J_{H,F}$ = 10.9, $^4J_{H,F}$ = 7.2 Hz, 1 H; C_{ar}H), 7.69 (dd, $^3J_{H,F}$ = 9.0, $^4J_{H,F}$ = 8.0 Hz, 1 H; C_{ar}H), 7.06 (br.s, 1 H; NH), 5.57 ppm (br.s, 1 H; NH). ^{13}C NMR (101 MHz, $CDCl_3$): δ =173.8 (q; C_{ar}), 165.8 (q; CO), 157.8 (m, q; C_{ar}), 152.1–149.5 (dd, $^1J_{C,F}$ = 247.9 Hz, $^2J_{C,F}$ = 14.4 Hz, q; C_{ar}), 150.3–147.6 (dd, $^1J_{C,F}$ = 254.7 Hz, $^2J_{C,F}$ = 13.7 Hz, q; C_{ar}), 131.9 (m, q; C_{ar}), 119.1 (d, $^2J_{C,F}$ = 19.9 Hz, t; C_{ar}), 115.5 ppm (d, $^2J_{C,F}$ = 18.1 Hz, t; C_{ar}). ^{125}Te NMR (126 MHz, $CDCl_3$): δ = 989.1 ppm. ^{19}F NMR (151 MHz, $CDCl_3$): δ = -136.7 (m), -137.5 ppm (m). IR (ATR): ṽ = 3458, 3115, 1665, 1582, 1562, 1499, 1456, 1406, 1283, 1194, 1136, 1105, 1045, 986, 868, 808, 758, 673, 611, 575 cm^{-1}. UV/Vis (CH_3CN): λ_{max} (log ε) = 360 (3.42), 291 (3.87), 247 nm (4.01). HRMS (ESI-TOF) *m*/*z*: $[C_8H_4F_2N_2O^{130}Te+H]^+$ calculated: 312.9427; found: 312.9422, $[C_8H_4F_2N_2O^{130}Te+Na]^+$ calculated: 334.9246; found: 334.9242.

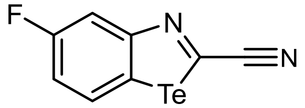

1b

Synthesis of benzotellurazole 1b. Benzotellurazole **8b** (185 mg, 0.6 mmol) was dissolved in anhydrous pyridine (7 mL) and stirred under argon at 0 °C, followed by dropwise addition of $POCl_3$ (1.4 mL). After 30 minutes, the ice bath was removed and the reaction was stirred overnight at room temperature. The reaction was then quenched by adding cold water dropwise, after which the solution was extracted with CH_2Cl_2. The aqueous layer was saturated with NaCl and repeatedly re-extracted with CH_2Cl_2 (2×20 mL). The organic layers were combined, dried over $MgSO_4$ and concentrated under vacuum. The residue was then purified using flash column chromatography with silica gel (CH_2Cl_2) to yield benzotellurazole **1b** as a yellow solid (95 mg, 0.4 mmol, 55%). M.p.: 238–239 °C. ^{1}H NMR (400 MHz, $CDCl_3$): δ = 8.15 (dd, $^3J_{H,F}$ = 9.4, $^4J_{H,H}$ = 2.6 Hz, 1 H; C_{ar}H), 7.93 (dd, $^3J_{H,H}$ = 8.8 Hz, $^4J_{H,F}$ = 5.4 Hz, 1 H; C_{ar}H), 7.23 ppm (td, $^3J_{H,F}$ = 8.5, $^4J_{H,H}$ = 2.6 Hz, 1 H; C_{ar}H). ^{13}C NMR (101 MHz, $CDCl_3$): δ = 164.2–162.6 (d, $^1J_{C,F}$ = 247.2 Hz, q; C_{ar}), 161.2 (d,

$^{3}J_{C,F}$ = 10.3 Hz, q; C_{ar}), 139.4 (q; C_{ar}), 132.3 (m, q; C_{ar}), 132.2 (m, t; C_{ar}), 119.8 (q; CN), 116.9 (d, $^{2}J_{C,F}$ = 23.7 Hz, t; C_{ar}), 115.3 ppm (d, $^{2}J_{C,F}$ = 22.5 Hz, t; C_{ar}). ^{125}Te NMR (126 MHz, $CDCl_3$): δ = 1157.6 ppm. ^{19}F NMR (151 MHz, $CDCl_3$): δ = -113.9 ppm (m). IR (ATR): ṽ = 3073, 2920, 2222, 1902, 1717, 1605, 1587, 1555, 1462, 1431, 1302, 1258, 1150, 1119, 1103, 1067, 962, 858, 810, 770, 656, 617, 571 cm^{-1}. UV/Vis (CH_3CN): λ_{max} (log ε) = 378 (3.51), 294 (3.89), 240 nm (3.93). HRMS (ESI-TOF) *m*/*z*: $[C_8H_3FN_2{}^{130}Te+H]^+$ calculated: 276.9415; found: 276.9412.

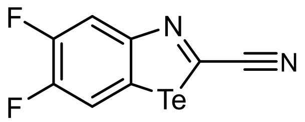

1c

Synthesis of benzotellurazole 1c. Benzotellurazole **8c** (310 mg, 1.0 mmol) was dissolved in anhydrous pyridine (10 mL) and stirred under argon at 0 °C, followed by dropwise addition of $POCl_3$ (2.3 mL). After 30 minutes, the ice bath was removed and the reaction was stirred overnight at room temperature. The reaction was then quenched by adding cold water dropwise, after which the solution was extracted with CH_2Cl_2. The aqueous layer was saturated with NaCl and repeatedly re-extracted with CH_2Cl_2 (2×20 mL). The organic layers were combined, dried over $MgSO_4$ and concentrated under vacuum. The residue was then purified using flash column chromatography with silica gel (CH_2Cl_2) to yield benzotellurazole **1c** as a yellow solid (218 mg, 0.6 mmol, 60%). M.p.: 246–248 °C. ^{1}H NMR (400 MHz, $CDCl_3$): δ = 8.22 (dd, $^{3}J_{H,F}$ = 10.5, $^{4}J_{H,F}$ = 7.2 Hz, 1 H; C_{ar}H) 7.71 ppm (dd, $^{3}J_{H,F}$ = 8.6, $^{4}J_{H,F}$ = 7.9 Hz, 1 H; C_{ar}H). ^{13}C NMR (101 MHz, $CDCl_3$): δ = 156.5 (m, q; C_{ar}), 152.4–150.7 (dd, $^{1}J_{C,F}$ = 250.4 Hz, $^{2}J_{C,F}$ = 13.9 Hz, q; C_{ar}), 151.2–149.4 (dd, $^{1}J_{C,F}$ = 258.3 Hz, $^{2}J_{C,F}$ = 13.8 Hz, q; C_{ar}), 138.2 (d, $^{4}J_{C,F}$ = 3.5 Hz, q; C_{ar}), 132.5 (m, q; C_{ar}), 119.5 (q; CN) 118.6 (d, $^{2}J_{C,F}$ = 20.3 Hz, t; C_{ar}), 116.5 ppm (d, $^{2}J_{C,F}$ = 18.2 Hz, t; C_{ar}). ^{125}Te NMR (126 MHz, $CDCl_3$): δ = 1189.6 ppm. ^{19}F NMR (151 MHz, $CDCl_3$): δ = -133.1 (m), -135.3 ppm (m). IR (ATR): ṽ = 3103, 3044, 2224, 1558, 1476, 1437, 1288, 1200, 1153, 1099, 1059, 876, 818, 764, 644, 596 cm^{-1}. UV/Vis (CH_3CN): λ_{max} (log ε) = 365 (3.52), 297 (3.94), 245 nm (3.99). HRMS (ESI-TOF) *m*/*z*: $[C_8H_2F_2N_2{}^{130}Te+H]^+$ calculated: 294.9321; found: 294.9319.

3. Computational Details

All calculation concerning the electrostatic potential, the formation of the dimers I and II and the isodesmic reactions were performed using the programs Gaussian 16[70]. The geometrical parameters were optimized by means of B3LYP[71-73] with additional dispersion correction with Becke-Johnson damping[74]. The basis sets applied were def2-TZVP[68] for the light elements (H, C, O, S and Se) and aug-cc-pVTZ-PP[75] for tellurium. This method is abbreviated in the following as B3LYP-D3BJ/def2-TZVP, aug-cc-pVTZ-PP. For all stationary points no symmetry restriction was applied. Frequency calculations were carried out at each of the structures to verify the nature of the stationary point. It turned out that all structures have none imaginary frequency.

The calculation regarding the binding energies of tetramethylammonium fluoride (TMAF) with the benzotellurazoles **1a–c** and **2** were performed using the program Orca 6.0[76]. The geometrical parameters were optimized using B97-D3[67] and the basis set def2-TZVP[68]. To take solvent effects into account, the solvent model CPCM[69] (chloroform as solvent) was used. For all structures C_1 symmetry was applied. Frequency calculations were carried out at each of the structures to verify the nature of the stationary point. It turned out that all structures have none imaginary frequency.

For the quantum theory of atoms in molecules (QTAIM)[64] and the interacting quantum atoms (IQA)[65] analyses the program Amsterdam Modeling Suite (AMS; Amsterdam Density Functional (ADF))[77-78] was employed. A benzotellurazole recognition unit together with all neighbors to which it is connected via ChBs was defined as a model structure. This system was subsequently calculated, with the geometric parameters taken from the crystal structure data in order to represent the energy of the interacting atoms in solid state as accurately as possible. As density functional B3LYP[71-73] was employed. To consider the dispersion interaction in an appropriate way, the additional dispersion correction with Becke-Johnson damping (D3BJ)[74] was employed. As basis set TZ2P[79] was applied.

Table S6. Absolute energies [au] calculated by means of B3LYP-D3BJ/def2-TZVP,aug-cc-pVTZ-PP.

Compound	*E*	*G*
pyridine	-248.396173	-248.334895
**2**	-632.101471	-632.008918
**1a**	-685.036167	-684.972901
**1b**	-784.316194	-784.262442
1c	-883.590295	-883.545929
pyridineH^+	-248.766318	-248.690856
**2**H^+	-632.476330	-632.371885
**1a**H^+	-685.388912	-685.312575
**1b**H^+	-784.664036	-784.597280
**1c**H^+	-883.933936	-883.876547
**2**•pyridine	-880.508199	-880.340899
**1a**•pyridine	-933.447409	-933.307075
**1b**•pyridine	-1032.728279	-1032.597248
**1c**•pyridine	-1132.003377	-1131.881519
**1a•1a** (dimer **I**)	-1370.090553	-1369.946211
**1b•1b** (dimer **I**)	-1568.651448	-1568.526066
**1c•1c** (dimer **I**)	-1767.200705	-1767.093759
**1a•1a** (dimer **II**)	-1370.085887	-1369.942763
**1b•1b** (dimer **II**)	-1568.645756	-1568.521845
**1c•1c** (dimer **II**)	-1767.194136	-1767.089128

Table S7. Absolute energies [au] calculated by means of B97-D3(CPCM,$CHCl_3$)/def2-TZVP.

Compound	*E*	*G*
TMAF	-314.176713	-314.046893
**2**•TMAF	-946.418741	-946.178050
**1a**•TMAF	-999.333426	-999.120113
**1b**•TMAF	-1098.567894	-1098.363910
**1c**•TMAF	-1197.796428	-1197.601831

Cartesian coordinates of the optimized geometry for pyridine at B3LYP-D3BJ/def2-TZVP,aug-cc-pVTZ-PP level of theory (number of imaginary frequencies = 0):

N	-0.00013600	-1.41167700	0.00000400
C	-1.13971600	-0.71924800	-0.00000700
C	1.13957700	-0.71945500	0.00000600
C	-1.19335900	0.66963700	0.00000000
H	-2.05400400	-1.30321200	0.00000500
C	1.19348900	0.66941900	-0.00000100
H	2.05374800	-1.30359900	-0.00002100
C	0.00013400	1.37839100	0.00000900
H	-2.14828700	1.17782200	-0.00002400
H	2.14853500	1.17738500	-0.00003900
H	0.00020700	2.46088200	0.00000700

Cartesian coordinates of the optimized geometry for **2** at B3LYP-D3BJ/def2-TZVP,aug-cc-pVTZ-PP level of theory (number of imaginary frequencies = 0):

Te	1.17222700	-0.83109400	0.00001900
N	0.00908100	1.83456200	-0.00006000
C	1.17116100	1.31594000	0.00005000
C	-0.87571500	-0.41789500	-0.00000500
C	-1.08459800	0.97972100	0.00002900
C	-2.39147000	1.47224500	0.00005000
H	-2.54057500	2.54361800	0.00009300
C	-3.46154900	0.59361800	0.00004400
H	-4.47268600	0.97874400	0.00013600

C	-3.24438800	-0.78500300	-0.00004400
H	-4.08668900	-1.46466900	-0.00018200
C	-1.95331200	-1.29558600	-0.00007900
H	-1.79643900	-2.36596300	-0.00016500
C	2.43860900	2.10113800	-0.00006100
H	2.20430100	3.16520700	0.00070900
H	3.03968300	1.86703300	-0.88105600
H	3.04060300	1.86591900	0.88001300

Cartesian coordinates of the optimized geometry for **1a** at B3LYP-D3BJ/def2-TZVP,aug-cc-pVTZ-PP level of theory (number of imaginary frequencies = 0):

N	0.12567700	1.75323700	0.00006300
C	1.19360200	1.04047300	-0.00001800
Te	0.89916300	-1.06245900	0.00002500
C	-1.06844000	1.06494000	0.00004900
C	-1.06064500	-0.35154200	-0.00003400
C	-2.29418000	1.74280900	0.00006100
C	-2.25421000	-1.06594700	-0.00009400
C	-3.47436000	1.02587900	0.00000400
H	-2.28586300	2.82415100	0.00011300
C	-3.45481000	-0.37253100	-0.00007100
H	-2.25514300	-2.14747500	-0.00013500
H	-4.42128300	1.54876400	0.00002100
H	-4.38645200	-0.92311200	-0.00011000
C	2.50268000	1.58520900	-0.00006400
N	3.59638800	1.95102100	-0.00009000

Cartesian coordinates of the optimized geometry for **1b** at B3LYP-D3BJ/def2-TZVP,aug-cc-pVTZ-PP level of theory (number of imaginary frequencies = 0):

N	-0.18582800	1.69055200	-0.00014200
C	-1.34252100	1.13163800	-0.00033300
Te	-1.34566300	-0.98972800	0.00024300
C	0.89927900	0.84186300	-0.00006400
C	0.69312500	-0.55990500	-0.00007300
C	2.20454800	1.35048700	-0.00011000
C	1.77736600	-1.43196900	-0.00012900
C	3.25072400	0.45871500	-0.00016800
H	2.37457600	2.41722300	-0.00012400
C	3.06528400	-0.92158100	-0.00018200
H	1.63079900	-2.50321600	-0.00008400
H	3.92806800	-1.57280000	-0.00020600
C	-2.56295600	1.85358000	-0.00027700
N	-3.59575100	2.36647800	-0.00018800
F	4.51144400	0.93205200	-0.00021200

Cartesian coordinates of the optimized geometry for **1c** at B3LYP-D3BJ/def2-TZVP,aug-cc-pVTZ-PP level of theory (number of imaginary frequencies = 0):

N	0.61074800	1.74101700	0.00012400
C	1.70637000	1.07092100	0.00025400
Te	1.49502800	-1.04204200	-0.00016300
C	-0.55273800	1.00621900	0.00006200
C	-0.48918400	-0.40864300	0.00003900
C	-1.80186500	1.64247100	0.00008900
C	-1.65243100	-1.17210300	0.00005200
C	-2.94101200	0.87671000	0.00010600
H	-1.86421000	2.72124900	0.00011500
C	-2.86740300	-0.52024100	0.00009200
H	-1.64313600	-2.25285500	0.00000600
C	2.99319300	1.66501100	0.00015800
N	4.07254000	2.07104000	0.00004900

F	-4.14885600	1.45387700	0.00013700
F	-4.00522200	-1.22373000	0.00009000

Cartesian coordinates of the optimized geometry for pyridineH^{+} at B3LYP-D3BJ/def2-TZVP,aug-cc-pVTZ-PP level of theory (number of imaginary frequencies = 0):

N	-1.30351400	0.00000200	-0.00000300
C	-0.66359000	-1.18430800	-0.00000500
C	-0.66358600	1.18431000	0.00000200
C	0.71341100	-1.20592100	-0.00000200
H	-1.27954700	-2.07136100	-0.00000900
C	0.71341500	1.20591900	0.00000500
H	-1.27954000	2.07136500	0.00000400
C	1.40891000	-0.00000200	0.00000300
H	1.22962600	-2.15474400	-0.00000300
H	1.22963200	2.15474100	0.00001000
H	2.49072100	-0.00000400	0.00000500
H	-2.31765600	0.00000400	-0.00000600

Cartesian coordinates of the optimized geometry for **2**H^{+} at B3LYP-D3BJ/def2-TZVP,aug-cc-pVTZ-PP level of theory (number of imaginary frequencies = 0):

Te	-1.16469600	-0.84516800	0.00000800
N	-0.03891100	1.74534100	-0.00004900
C	-1.24635100	1.21987700	-0.00003200
C	0.88779200	-0.43451100	0.00001200
C	1.11092100	0.94732900	-0.00002500
C	2.40174600	1.47130300	-0.00004700
H	2.56556700	2.54135400	-0.00007600
C	3.46597500	0.58859200	-0.00002800
H	4.47642000	0.97246100	-0.00004100
C	3.24880000	-0.79232700	0.00000900
H	4.09437300	-1.46637300	0.00002200
C	1.96402900	-1.31401800	0.00002700
H	1.81168100	-2.38426300	0.00005500
C	-2.46732100	2.06217900	0.00004400
H	-2.21782800	3.12391000	-0.00076800
H	-3.07738100	1.84993400	0.88006700
H	-3.07834800	1.84880000	-0.87901800
H	0.06855600	2.75499700	-0.00007700

Cartesian coordinates of the optimized geometry for **1a**H^{+} at B3LYP-D3BJ/def2-TZVP,aug-cc-pVTZ-PP level of theory (number of imaginary frequencies = 0):

N	-0.15294600	1.66250300	-0.00006900
C	-1.26330400	0.93465500	-0.00003300
Te	-0.88522100	-1.08731400	0.00005300
C	1.08851800	1.04550300	-0.00005100
C	1.07211200	-0.36119400	0.00000800
C	2.29313000	1.75396200	-0.00008900
C	2.26775500	-1.07225300	0.00002900
C	3.47062500	1.03635900	-0.00006700
H	2.29694500	2.83631000	-0.00013400
C	3.45851800	-0.36532500	-0.00000900
H	2.27633300	-2.15319600	0.00007400
H	4.41498900	1.56228900	-0.00009500
H	4.39506200	-0.90599800	0.00000800
C	-2.53587600	1.52943500	-0.00005100
N	-3.59681100	1.98285400	-0.00006400
H	-0.21238700	2.67660700	-0.00011200

Cartesian coordinates of the optimized geometry for **1b**H+ at B3LYP-D3BJ/def2-TZVP,aug-cc-pVTZ-PP level of theory (number of imaginary frequencies = 0):

N	0.22050900	1.60551800	0.00003800
C	1.42301500	1.03865600	0.00000500
Te	1.33222600	-1.01380700	-0.00003200
C	-0.92132200	0.81955500	0.00003100
C	-0.70486000	-0.57065100	-0.00001000
C	-2.20930800	1.35723000	0.00005800
C	-1.79155900	-1.44344000	-0.00002600
C	-3.25742300	0.46387300	0.00004100
H	-2.39524100	2.42271100	0.00008900
C	-3.07082500	-0.92275600	0.00000000
H	-1.64887800	-2.51484700	-0.00005600
H	-3.93827000	-1.56824300	-0.00001000
C	2.59954800	1.80554700	0.00000800
N	3.58653400	2.40320900	0.00001000
F	-4.49832400	0.93457500	0.00006600
H	0.13863500	2.61800800	0.00006200

Cartesian coordinates of the optimized geometry for **1c**H+ at B3LYP-D3BJ/def2-TZVP,aug-cc-pVTZ-PP level of theory (number of imaginary frequencies = 0):

N	0.63444000	1.65157800	0.00004500
C	1.77468700	0.96910200	0.00001300
Te	1.47847300	-1.06670200	-0.00004200
C	-0.57871900	0.98446000	0.00003500
C	-0.50472400	-0.42035000	-0.00000900
C	-1.80923600	1.64858800	0.00006600
C	-1.66779600	-1.18406400	-0.00002400
C	-2.94842000	0.88191300	0.00005100
H	-1.88754700	2.72764800	0.00010000
C	-2.87890100	-0.52532700	0.00000600
H	-1.66100200	-2.26502900	-0.00005800
C	3.02164300	1.61395600	0.00002100
N	4.06394500	2.10910200	0.00002700
F	-4.13900600	1.45306600	0.00007800
F	-4.00836000	-1.20815400	-0.00000600
H	0.65435600	2.66727000	0.00007500

Cartesian coordinates of the optimized geometry for **2**•pyridine at B3LYP-D3BJ/def2-TZVP,aug-cc-pVTZ-PP level of theory (number of imaginary frequencies = 0):

N	-3.23180300	-0.19334600	0.10388600
C	-2.60243200	-1.30526400	0.08712600
Te	-0.47083200	-1.13142700	-0.04707700
C	-2.46774200	0.96569400	0.02825700
C	-1.06090700	0.87601700	-0.06061200
C	-3.06872700	2.22691700	0.03622600
C	-0.28139900	2.02271200	-0.14362600
C	-2.28800100	3.36779500	-0.04389500
H	-4.14691100	2.28387300	0.10486200
C	-0.89893400	3.26661400	-0.13441500
H	0.79330300	1.94155000	-0.21781000
N	2.40525700	0.03152100	-0.08576000
C	3.19941200	-0.12409400	-1.14439900
C	2.97279400	0.23832500	1.10241000
C	4.58549400	-0.07797000	-1.06422100
H	2.70295600	-0.29295800	-2.09359100
C	4.34831400	0.30101000	1.28347800
H	2.29488500	0.35772200	1.94042800
C	5.17027600	0.13915400	0.17610300
H	5.18709500	-0.20963800	-1.95337000
H	4.76063900	0.47166600	2.26870900
H	6.24700600	0.18099600	0.27810200

C	-3.29389000	-2.62640000	0.16020800
H	-4.37037400	-2.47230100	0.23194900
H	-3.07688200	-3.22728200	-0.72589100
H	-2.95500200	-3.19499400	1.02914300
H	-0.29489400	4.16274000	-0.19942300
H	-2.75827400	4.34254800	-0.03781500

Cartesian coordinates of the optimized geometry for **1a**•pyridine at B3LYP-D3BJ/def2-TZVP,aug-cc-pVTZ-PP level of theory (number of imaginary frequencies = 0):

N	3.11023300	0.26696900	0.00112200
C	2.58416300	-0.90916600	0.00102900
Te	0.45614700	-1.00091700	-0.00070300
C	2.21089800	1.31621300	0.00015900
C	0.81943400	1.05748600	-0.00088900
C	2.66211600	2.64225100	0.00023500
C	-0.09124300	2.10841800	-0.00195900
C	1.75068200	3.68004300	-0.00072900
H	3.72862000	2.82206000	0.00105800
C	0.37821400	3.41339300	-0.00185100
H	-1.15232100	1.90836000	-0.00300400
C	3.36722300	-2.09416700	0.00172200
N	3.92126400	-3.10622300	0.00219000
N	-2.39339900	-0.25979500	-0.00089000
C	-3.07401300	-0.23587600	1.14529900
C	-3.07691600	-0.24211900	-1.14545200
C	-4.46060900	-0.18990100	1.19698500
H	-2.48233000	-0.25361900	2.05364400
C	-4.46364900	-0.19642400	-1.19386600
H	-2.48755000	-0.26482400	-2.05519100
C	-5.16808100	-0.16986600	0.00239000
H	-4.96886600	-0.17144100	2.15131100
H	-4.97432900	-0.18316800	-2.14698400
H	-6.24973100	-0.13494000	0.00367000
H	-0.32909200	4.23284500	-0.00268800
H	2.09997100	4.70404600	-0.00065600

Cartesian coordinates of the optimized geometry for **1b**•pyridine at B3LYP-D3BJ/def2-TZVP,aug-cc-pVTZ-PP level of theory (number of imaginary frequencies = 0):

N	2.96609100	-0.48326100	0.00109200
C	2.24296000	-1.55065800	0.00087400
Te	0.13236800	-1.27593300	-0.00058100
C	2.26034300	0.70396300	0.00027400
C	0.84496200	0.68800400	-0.00074700
C	2.94043700	1.92892700	0.00041900
C	0.13010000	1.88093200	-0.00176500
C	2.20012500	3.08701200	-0.00047300
H	4.02057100	1.95089000	0.00119600
C	0.80844400	3.08962100	-0.00161400
H	-0.94931300	1.86793300	-0.00280000
C	2.81026000	-2.85290900	0.00145200
N	3.18054000	-3.94554000	0.00172000
N	-2.53516400	-0.05091600	-0.00065200
C	-3.20356900	0.08181000	1.14558500
C	-3.20675100	0.07663900	-1.14560700
C	-4.56460800	0.35029900	1.19673500
H	-2.62310300	-0.03162700	2.05422800
C	-4.56793600	0.34490500	-1.19418100
H	-2.62883200	-0.04089700	-2.05535100
C	-5.25920600	0.48422300	0.00193000
H	-5.06355300	0.44995700	2.15091000
H	-5.06953400	0.44026500	-2.14740500
H	-6.32112200	0.69267900	0.00293800

F	2.84215300	4.27428900	-0.00039000
H	0.27871100	4.03215000	-0.00240900

Cartesian coordinates of the optimized geometry for **1c**•pyridine at B3LYP-D3BJ/def2-TZVP,aug-cc-pVTZ-PP level of theory (number of imaginary frequencies = 0):

N	2.95964900	-0.66494300	0.00059000
C	2.24584100	-1.73815600	0.00043900
Te	0.13092300	-1.47791600	-0.00027900
C	2.24514100	0.51578400	0.00017300
C	0.83056700	0.48927400	-0.00036300
C	2.91486000	1.74730500	0.00025800
C	0.10200100	1.67371000	-0.00086200
C	2.18159100	2.90811000	-0.00019500
H	3.99506000	1.77795000	0.00066900
C	0.78476800	2.87086400	-0.00076300
H	-0.97728300	1.67906600	-0.00140400
C	2.82102400	-3.03630900	0.00080800
N	3.19737300	-4.12691400	0.00105500
N	-2.52564500	-0.24887100	-0.00034600
C	-3.19389600	-0.11305900	1.14588900
C	-3.19611100	-0.11751500	-1.14579700
C	-4.55336400	0.16251200	1.19632300
H	-2.61449500	-0.22918800	2.05483000
C	-4.55568200	0.15786600	-1.19466500
H	-2.61847900	-0.23718200	-2.05540400
C	-5.24659300	0.30056700	0.00122400
H	-5.05199900	0.26536100	2.15026600
H	-5.05616300	0.25701100	-2.14803400
H	-6.30717400	0.51551600	0.00183500
F	0.10853900	4.02977500	-0.00123400
F	2.79390300	4.10161300	-0.00011600

Cartesian coordinates of the optimized geometry for **1a**•**1a** (dimer **I**) at B3LYP-D3BJ/def2-TZVP,aug-cc-pVTZ-PP level of theory (number of imaginary frequencies = 0):

Te	-2.98398400	1.20754800	-0.00023100
N	-3.49694000	-1.67614000	-0.00039300
N	-0.05261000	-1.63134600	-0.00062100
C	-2.50201700	-0.85774200	-0.00041300
C	-4.86834900	0.30574400	-0.00016500
C	-4.75027200	-1.10505300	-0.00024300
C	-5.90548600	-1.89895600	-0.00013500
H	-5.79496600	-2.97479200	-0.00019000
C	-7.14731500	-1.29706500	0.00003400
H	-8.04149200	-1.90594300	0.00011000
C	-7.25813400	0.09844300	0.00011700
H	-8.23782600	0.55851500	0.00025400
C	-6.12819900	0.90043600	0.00002500
H	-6.23249500	1.97738700	0.00009200
C	-1.16025000	-1.31436400	-0.00050600
Te	2.98396600	-1.20754800	0.00049700
N	3.49696200	1.67613100	0.00041200
N	0.05266800	1.63153500	0.00159300
C	2.50202300	0.85775000	0.00073900
C	4.86834300	-0.30577000	-0.00012800
C	4.75028600	1.10502700	-0.00006600
C	5.90551000	1.89891600	-0.00052400
H	5.79500500	2.97475300	-0.00047500
C	7.14733100	1.29700800	-0.00101800
H	8.04151700	1.90587400	-0.00136400
C	7.25813100	-0.09850200	-0.00108300
H	8.23781700	-0.55858700	-0.00147700
C	6.12818500	-0.90047900	-0.00064800

H	6.23246700	-1.97743200	-0.00070700
C	1.16027500	1.31443900	0.00118200

Cartesian coordinates of the optimized geometry for **1b•1b** (dimer **I**) at B3LYP-D3BJ/def2-TZVP,aug-cc-pVTZ-PP level of theory (number of imaginary frequencies = 0):

Te	-2.90762100	1.36938200	-0.00030600
F	-8.33638500	-1.57988800	0.00091200
N	-3.57860500	-1.48213200	-0.00034900
N	-0.13732200	-1.62262900	-0.00129600
C	-2.54107200	-0.71700600	-0.00057100
C	-4.83807600	0.57203000	-0.00003200
C	-4.79767100	-0.84312200	-0.00003200
C	-5.99052100	-1.58027700	0.00027100
H	-5.96699900	-2.66032200	0.00024300
C	-7.17781100	-0.89000100	0.00059700
C	-7.24069100	0.50227200	0.00056500
H	-8.20631000	0.98857300	0.00078500
C	-6.06474900	1.23330400	0.00024600
H	-6.11298200	2.31383000	0.00023600
C	-1.22622100	-1.24675900	-0.00097900
Te	2.90763600	-1.36937300	0.00003600
F	8.33637600	1.57994200	0.00011900
N	3.57859700	1.48215100	0.00020700
N	0.13723100	1.62235000	0.00038100
C	2.54107700	0.71700900	0.00017100
C	4.83808600	-0.57200400	0.00022000
C	4.79766700	0.84314800	0.00020000
C	5.99051200	1.58031300	0.00018200
H	5.96698200	2.66035700	0.00019400
C	7.17780800	0.89004500	0.00015400
C	7.24069900	-0.50222700	0.00021300
H	8.20632200	-0.98851900	0.00023600
C	6.06476300	-1.23326900	0.00024900
H	6.11300500	-2.31379400	0.00028300
C	1.22619400	1.24666700	0.00024400

Cartesian coordinates of the optimized geometry for **1c•1c** (dimer **I**) at B3LYP-D3BJ/def2-TZVP,aug-cc-pVTZ-PP level of theory (number of imaginary frequencies = 0):

Te	1.57393500	2.10604900	1.83287700
F	7.06651400	4.65596600	0.57284700
F	5.88575400	5.37873100	2.86744700
N	3.39533000	1.78700200	-0.43971900
N	0.83932800	-0.33689200	-1.34873600
C	2.25411200	1.35134100	-0.02869000
C	3.40963300	3.07393600	1.59601800
C	4.02845900	2.68853700	0.38289300
C	5.27590600	3.22526300	0.02934800
H	5.75413300	2.93543100	-0.89531800
C	5.87561900	4.12188100	0.87618700
C	5.25931700	4.49993100	2.07509100
C	4.03504500	3.98642400	2.44326400
H	3.59816200	4.30812400	3.37850500
C	1.49691000	0.41934800	-0.78060100
Te	-1.57393500	-2.10604900	-1.83287700
F	-7.06651400	-4.65596600	-0.57284700
F	-5.88575400	-5.37873100	-2.86744700
N	-3.39533000	-1.78700200	0.43971900
N	-0.83932800	0.33689200	1.34873600
C	-2.25411200	-1.35134100	0.02869000
C	-3.40963300	-3.07393600	-1.59601800
C	-4.02845900	-2.68853700	-0.38289300
C	-5.27590600	-3.22526300	-0.02934800

H	-5.75413300	-2.93543100	0.89531800
C	-5.87561900	-4.12188100	-0.87618700
C	-5.25931700	-4.49993100	-2.07509100
C	-4.03504500	-3.98642400	-2.44326400
H	-3.59816200	-4.30812400	-3.37850500
C	-1.49691000	-0.41934800	0.78060100

Cartesian coordinates of the optimized geometry for **1a•1a** (dimer **II**) at B3LYP-D3BJ/def2-TZVP,aug-cc-pVTZ-PP level of theory (number of imaginary frequencies = 0):

Te	-1.82691700	-0.94985800	-0.39495900
N	-4.52867600	0.16907300	-0.20469800
N	-5.00447100	-2.50723400	-2.36341900
C	-3.91041200	-0.80233100	-0.78012300
C	-2.36944900	0.71233400	0.74389400
C	-3.75326000	0.97722400	0.60170700
C	-4.31724200	2.06022600	1.28817400
H	-5.37518000	2.25051700	1.16923100
C	-3.52342400	2.85148300	2.09525500
H	-3.95972600	3.68679000	2.62658000
C	-2.15958700	2.57557800	2.23466400
H	-1.54774000	3.19712100	2.87559400
C	-1.57894800	1.50892900	1.56548000
H	-0.52672000	1.29505900	1.68211000
C	-4.55744100	-1.72167400	-1.64673200
Te	3.92574300	-0.67090100	0.32146700
N	1.08148700	-0.04430100	0.27648200
N	1.14971400	-2.82033400	2.35290900
C	1.88135400	-0.90443000	0.80578600
C	3.05576300	0.90440800	-0.73283800
C	1.65450200	0.91597700	-0.53185500
C	0.87788600	1.90098500	-1.15615600
H	-0.19114700	1.90393300	-0.99759300
C	1.48959700	2.84420900	-1.95728900
H	0.89198300	3.60488200	-2.44087300
C	2.87481200	2.82367700	-2.15006100
H	3.34028100	3.56886400	-2.78160000
C	3.66300300	1.85888100	-1.54208300
H	4.73271800	1.85551500	-1.70054800
C	1.44122000	-1.94942100	1.65547700

Cartesian coordinates of the optimized geometry for **1b•1b** (dimer **II**) at B3LYP-D3BJ/def2-TZVP,aug-cc-pVTZ-PP level of theory (number of imaginary frequencies = 0):

Te	1.59753900	-1.31115200	0.46996800
F	4.36733700	3.35998800	-2.45721900
N	4.37379000	-0.37434500	0.49263900
N	4.50131400	-3.08544700	2.65727900
C	3.64904900	-1.30355200	1.01171200
C	2.33336300	0.31326300	-0.61160200
C	3.71721200	0.48513300	-0.36328800
C	4.40978700	1.52715700	-0.99341200
H	5.46505800	1.66844200	-0.81036500
C	3.71367600	2.35257400	-1.84426000
C	2.35589600	2.19169400	-2.10608000
H	1.86330600	2.86752000	-2.79122000
C	1.66441800	1.16289600	-1.48675100
H	0.61260800	1.02159600	-1.68654300
C	4.16494800	-2.26779600	1.91663300
Te	-4.05484300	-0.65400200	-0.51886800
F	-0.75754700	3.59999000	2.48107900
N	-1.18408300	-0.17785300	-0.38308000
N	-1.33236300	-2.93304000	-2.47939600
C	-2.01436500	-0.99056700	-0.94266300

C	-3.13648400	0.86570000	0.57294100
C	-1.73086700	0.80522300	0.41480400
C	-0.91602000	1.73980300	1.06730200
H	0.15805700	1.70869800	0.95870100
C	-1.52310800	2.69468800	1.84785300
C	-2.90459400	2.76786300	2.01429400
H	-3.32216800	3.54215100	2.64254500
C	-3.71527000	1.84706100	1.37213000
H	-4.78737800	1.90153200	1.49994300
C	-1.60237300	-2.05229800	-1.78579800

Cartesian coordinates of the optimized geometry for **1c•1c** (dimer **II**) at B3LYP-D3BJ/def2-TZVP,aug-cc-pVTZ-PP level of theory (number of imaginary frequencies = 0):

Te	1.75746100	-1.42124200	0.63058700
F	4.16938600	3.41360600	-2.36177800
F	1.58996700	2.84582200	-2.84400400
N	4.45176400	-0.26808900	0.65634800
N	4.77244400	-2.92546400	2.86936500
C	3.79991300	-1.24260800	1.18761600
C	2.37349000	0.23715100	-0.47274400
C	3.73678600	0.52163200	-0.21785200
C	4.34923300	1.60588000	-0.86160600
H	5.38877100	1.83332800	-0.67367100
C	3.61232300	2.37025700	-1.73220400
C	2.26887700	2.07576700	-1.98283500
C	1.64253900	1.01524500	-1.36433900
H	0.60664700	0.80887100	-1.58729600
C	4.38104300	-2.14831500	2.11229800
Te	-3.90043800	-1.06398700	-0.76774800
F	-1.06134600	3.33501600	2.49452000
F	-3.74002300	3.37587300	2.47573600
N	-1.07538600	-0.51600900	-0.31963100
N	-0.92137200	-3.31310100	-2.35996000
C	-1.81694400	-1.36085300	-0.95157400
C	-3.14480200	0.49741300	0.38643600
C	-1.72985400	0.46690000	0.38948800
C	-1.01512900	1.43523300	1.10940600
H	0.06508100	1.42672800	1.12439400
C	-1.71006600	2.39721700	1.79833400
C	-3.10993600	2.41932700	1.78924300
C	-3.83594500	1.47881100	1.09005000
H	-4.91508800	1.53407100	1.11083300
C	-1.28896500	-2.42660900	-1.72055500

Cartesian coordinates of the optimized geometry for TMAF at B97-D3(CPCM,$CHCl_3$)/def2-TZVP level of theory (number of imaginary frequencies = 0):

F	1.13230000	2.27140000	0.04020000
C	2.01560000	-0.56510000	-0.09060000
C	3.46360000	0.86380000	1.26760000
H	3.33180000	0.17660000	2.10500000
H	4.44750000	1.33360000	1.31180000
H	2.65420000	1.60230000	1.20790000
C	3.54110000	1.01340000	-1.17350000
H	4.52370000	1.48190000	-1.09740000
H	3.46580000	0.43290000	-2.09440000
H	2.72650000	1.74290000	-1.07670000
C	4.44650000	-0.98330000	-0.04270000
H	5.42010000	-0.49490000	0.01890000
H	4.30270000	-1.65110000	0.80790000
H	4.35820000	-1.53640000	-0.97870000
H	1.90090000	-1.23380000	0.76410000
H	1.30240000	0.26910000	-0.06320000

H	1.95690000	-1.12690000	-1.02440000
N	3.38170000	0.06750000	-0.00990000

Cartesian coordinates of the optimized geometry for **2** at B97-D3(CPCM,$CHCl_3$)/def2-TZVP level of theory (number of imaginary frequencies = 0):

N	0.63490000	1.73440000	0.00010000
C	1.74070000	1.08290000	-0.00010000
Te	1.49810000	-1.05560000	0.00010000
C	-0.54570000	1.00240000	0.00020000
C	-0.49290000	-0.41700000	0.00020000
C	-1.79790000	1.63650000	0.00020000
C	-1.66370000	-1.17580000	0.00010000
C	-2.96200000	0.87620000	0.00010000
H	-1.83480000	2.72240000	0.00030000
C	-2.89680000	-0.52390000	0.00000000
H	-1.62180000	-2.26120000	0.00000000
H	-3.81200000	-1.11030000	-0.00010000
H	-3.92890000	1.37210000	0.00010000
C	3.09380000	1.71240000	-0.00010000
H	2.99460000	2.80190000	-0.00240000
H	3.66400000	1.40370000	0.88360000
H	3.66580000	1.40010000	-0.88130000

Cartesian coordinates of the optimized geometry for **1a** at B97-D3(CPCM,$CHCl_3$)/def2-TZVP level of theory (number of imaginary frequencies = 0):

N	0.61670000	1.74930000	0.00010000
C	1.71770000	1.06240000	0.00020000
Te	1.49660000	-1.05200000	-0.00020000
C	-0.54720000	1.01480000	0.00000000
C	-0.48620000	-0.40900000	0.00000000
C	-1.80620000	1.64590000	0.00010000
C	-1.65340000	-1.17450000	0.00010000
C	-2.95990000	0.87850000	0.00010000
H	-1.84590000	2.73110000	0.00010000
C	-2.88490000	-0.52450000	0.00010000
H	-1.60810000	-2.25920000	0.00010000
C	3.00240000	1.65670000	0.00020000
N	4.08550000	2.08400000	0.00020000
H	-3.79790000	-1.11360000	0.00020000
H	-3.93100000	1.36500000	0.00020000

Cartesian coordinates of the optimized geometry for **1b** at B97-D3(CPCM,$CHCl_3$)/def2-TZVP level of theory (number of imaginary frequencies = 0):

N	0.61450000	1.75110000	0.00010000
C	1.71500000	1.06180000	0.00010000
Te	1.49450000	-1.05000000	0.00010000
C	-0.54770000	1.01580000	0.00010000
C	-0.48620000	-0.40790000	0.00010000
C	-1.80250000	1.65520000	0.00020000
C	-1.65460000	-1.17240000	0.00010000
C	-2.93320000	0.86550000	0.00010000
H	-1.86760000	2.73790000	0.00020000
C	-2.88970000	-0.53280000	0.00000000
H	-1.61290000	-2.25680000	0.00000000
C	2.99990000	1.65620000	0.00000000
N	4.08300000	2.08300000	0.00000000
F	-4.15040000	1.46780000	0.00010000
H	-3.81510000	-1.09990000	0.00000000

Cartesian coordinates of the optimized geometry for **1c** at B97-D3(CPCM,$CHCl_3$)/def2-TZVP level of theory (number of imaginary frequencies = 0):

N	0.61140000	1.74940000	0.00030000
C	1.71320000	1.06290000	0.00030000
Te	1.49290000	-1.05150000	0.00030000
C	-0.54890000	1.01420000	0.00030000
C	-0.48670000	-0.40930000	0.00030000
C	-1.80490000	1.65260000	0.00030000
C	-1.65030000	-1.17970000	0.00010000
C	-2.94460000	0.88010000	0.00010000
H	-1.87140000	2.73540000	0.00030000
C	-2.86930000	-0.52410000	0.00000000
H	-1.63270000	-2.26440000	-0.00010000
C	2.99780000	1.65600000	-0.00010000
N	4.08210000	2.07990000	-0.00040000
F	-4.16220000	1.45960000	-0.00010000
F	-4.01480000	-1.23220000	-0.00040000

Cartesian coordinates of the optimized geometry for **2**•TMAF at B97-D3(CPCM,$CHCl_3$)/def2-TZVP level of theory (number of imaginary frequencies = 0):

N	-0.82390000	1.44580000	1.62790000
C	-1.84260000	0.65850000	1.70710000
Te	-1.77460000	-1.08400000	0.45780000
C	0.19730000	1.08070000	0.75150000
C	0.07420000	-0.12090000	0.01160000
C	1.33760000	1.88670000	0.60020000
C	1.09560000	-0.49500000	-0.86790000
C	2.34570000	1.50060000	-0.27750000
H	1.41620000	2.80560000	1.17660000
C	2.22610000	0.31060000	-1.01110000
H	1.01640000	-1.41560000	-1.44320000
F	-4.04760000	-1.62380000	1.28960000
H	3.01880000	0.01360000	-1.69420000
H	3.22950000	2.12290000	-0.39460000
C	-2.98890000	0.92500000	2.62770000
H	-3.93490000	0.95510000	2.08010000
H	-3.08770000	0.11910000	3.36280000
H	-2.82740000	1.87560000	3.14590000
C	-6.12430000	-1.73580000	-0.99980000
C	-6.84130000	-0.44230000	0.95630000
H	-7.09990000	0.56010000	1.30030000
H	-7.68810000	-1.11860000	1.08080000
H	-5.94570000	-0.82540000	1.45450000
C	-5.35740000	0.57260000	-0.71090000
H	-5.13050000	0.61850000	-1.77650000
H	-5.64300000	1.55680000	-0.33790000
H	-4.51600000	0.16600000	-0.14980000
C	-7.70950000	0.13120000	-1.27860000
H	-8.53510000	-0.56400000	-1.12170000
H	-7.97060000	1.12300000	-0.90740000
H	-7.44710000	0.17770000	-2.33620000
H	-5.27050000	-2.05130000	-0.39410000
H	-6.97690000	-2.40220000	-0.86270000
H	-5.86460000	-1.66060000	-2.05660000
N	-6.51870000	-0.36440000	-0.51420000

Cartesian coordinates of the optimized geometry for **1a**•TMAF at B97-D3(CPCM,$CHCl_3$)/def2-TZVP level of theory (number of imaginary frequencies = 0):

N	-0.91360000	-0.94730000	-1.00850000
C	-1.67660000	-0.89350000	0.05790000
Te	-0.86770000	-1.67720000	1.92910000

C	0.35360000	-1.44230000	-0.74810000
C	0.70200000	-1.87520000	0.55820000
C	1.30790000	-1.53460000	-1.78120000
C	1.98360000	-2.37010000	0.81920000
C	2.57540000	-2.02980000	-1.51380000
H	1.02720000	-1.20550000	-2.77840000
C	2.91470000	-2.44220000	-0.21340000
H	2.22870000	-2.69710000	1.82390000
C	-3.01550000	-0.43730000	-0.03650000
N	-4.12680000	-0.08140000	-0.00030000
F	0.44670000	-2.86770000	3.33540000
H	3.91180000	-2.82530000	-0.01020000
H	3.31110000	-2.09460000	-2.31110000
C	-0.30870000	-5.63710000	1.60460000
C	-0.91360000	-7.26930000	-0.12890000
H	-0.95740000	-7.43440000	-1.20580000
H	-0.23760000	-7.98430000	0.34040000
H	-1.90950000	-7.34140000	0.30890000
C	-1.30800000	-4.87950000	-0.52060000
H	-0.89620000	-3.88520000	-0.35810000
H	-1.36610000	-5.09990000	-1.58670000
H	-2.28960000	-4.96420000	-0.05490000
C	0.98680000	-5.74830000	-0.48000000
H	1.63880000	-6.48620000	-0.01170000
H	0.91250000	-5.92770000	-1.55280000
H	1.34780000	-4.73940000	-0.28220000
H	-1.31490000	-5.71380000	2.01690000
H	0.34100000	-6.39830000	2.03760000
H	0.09710000	-4.64230000	1.78870000
N	-0.38720000	-5.88360000	0.11970000

Cartesian coordinates of the optimized geometry for **1b**•TMAF at B97-D3(CPCM,$CHCl_3$)/def2-TZVP level of theory (number of imaginary frequencies = 0):

N	-0.91390000	-0.93660000	-1.00780000
C	-1.67380000	-0.88540000	0.06240000
Te	-0.86370000	-1.67290000	1.92880000
C	0.35200000	-1.43160000	-0.74970000
C	0.70230000	-1.86750000	0.55510000
C	1.29900000	-1.52080000	-1.79040000
C	1.98400000	-2.36230000	0.81330000
C	2.55020000	-2.02050000	-1.49500000
H	1.04230000	-1.19700000	-2.79400000
C	2.92040000	-2.43950000	-0.21330000
H	2.23220000	-2.69060000	1.81650000
C	-3.01320000	-0.42950000	-0.02870000
N	-4.12450000	-0.07460000	0.01030000
F	3.47420000	-2.11340000	-2.49510000
F	0.45680000	-2.86920000	3.31860000
H	3.92210000	-2.81950000	-0.03680000
C	-0.31710000	-5.64240000	1.60620000
C	-0.91530000	-7.27460000	-0.12970000
H	-0.95450000	-7.43980000	-1.20680000
H	-0.24330000	-7.99130000	0.34280000
H	-1.91340000	-7.34390000	0.30350000
C	-1.29960000	-4.88350000	-0.52600000
H	-0.88700000	-3.89060000	-0.35760000
H	-1.34810000	-5.10180000	-1.59300000
H	-2.28560000	-4.96700000	-0.06950000
C	0.99170000	-5.75960000	-0.47020000
H	1.64060000	-6.49460000	0.00700000
H	0.92440000	-5.94520000	-1.54230000
H	1.35080000	-4.74950000	-0.27670000
H	-1.32680000	-5.71280000	2.01100000
H	0.32450000	-6.40720000	2.04500000

H	0.09350000	-4.65000000	1.79300000
N	-0.38630000	-5.89020000	0.12110000

Cartesian coordinates of the optimized geometry for **1c**•TMAF at B97-D3(CPCM,$CHCl_3$)/def2-TZVP level of theory (number of imaginary frequencies = 0):

N	-0.90040000	-0.87800000	-1.01110000
C	-1.66870000	-0.84500000	0.05260000
Te	-0.87570000	-1.67610000	1.91040000
C	0.35840000	-1.39000000	-0.75510000
C	0.69440000	-1.85660000	0.54210000
C	1.31290000	-1.46590000	-1.78990000
C	1.96530000	-2.37480000	0.80620000
C	2.55970000	-1.98350000	-1.51450000
H	1.06860000	-1.11810000	-2.78870000
C	2.88570000	-2.43070000	-0.22350000
H	2.21850000	-2.73470000	1.79730000
C	-3.00290000	-0.37510000	-0.03670000
N	-4.11210000	-0.01360000	0.00260000
F	3.49640000	-2.07370000	-2.48930000
F	4.12360000	-2.93670000	-0.00610000
F	0.45040000	-2.89440000	3.27440000
C	-0.31300000	-5.66010000	1.61530000
C	-0.90860000	-7.30040000	-0.11430000
H	-0.94910000	-7.46950000	-1.19070000
H	-0.23300000	-8.01290000	0.35960000
H	-1.90560000	-7.37190000	0.32110000
C	-1.30230000	-4.91190000	-0.51750000
H	-0.89320000	-3.91640000	-0.35460000
H	-1.35380000	-5.13490000	-1.58350000
H	-2.28660000	-4.99670000	-0.05750000
C	0.99190000	-5.78000000	-0.46350000
H	1.64630000	-6.50750000	0.01740000
H	0.92390000	-5.97260000	-1.53430000
H	1.34420000	-4.76640000	-0.27740000
H	-1.32140000	-5.73660000	2.02240000
H	0.33460000	-6.41940000	2.05470000
H	0.09080000	-4.66420000	1.79900000
N	-0.38420000	-5.91310000	0.13100000

4. Crystal Structure Data

Crystal Structure Data of 2

Table S8. Crystal structure data and structure refinement for **2**.

Deposition Number	2483500
Identification code	sm_151m
Empirical formula	C_8H_7NTe
Formula weight	244.75
Density (calculated)	2.106 g·cm^{-3}
F(000)	912
Temperature	100(2) K
Crystal size	0.108 × 0.097 × 0.042 mm
Crystal color	colorless
Crystal description	plate
Wavelength	0.71073 Å
Crystal system	orthorhombic
Space group	*Pbca*
Unit cell dimensions	
a [Å]	5.8498(6)
b [Å]	11.2897(9)
c [Å]	23.372(3)
α [°]	90
β [°]	90
γ [°]	90
Volume	1543.6(3) Å^3
Z	8
Cell measurement reflections used	2797
Cell measurement θ min/max	3.49°/30.24°
Diffractometer control software	BRUKER APEX3(v2019.1-0)
Diffractometer measurement device	Bruker D8 KAPPA II (APEX II detector)
Diffractometer measurement method	Data collection strategy APEX 2/COSMO
θ range for data collection	3.487°- 30.501°
Completeness to θ = 25.242°	99.9%
Completeness to θ_{max} = 30.501°	99.9%
Index ranges	$-8 \leq h \leq 7$
	$-16 \leq k \leq 15$
	$-23 \leq l \leq 33$
Computing data reduction	BRUKER APEX3(v2019.1-0)
Absorption coefficient	3.771 mm^{-1}
Absorption correction	Semi-empirical from equivalents
Computation absorption correction	SADABS
Max./min. Transmission	0.75/0.50
R_{merg} before/after correction	0.0861/0.0674
Computing structure solution	BRUKER APEX3(v2019.1-0)
Computing structure refinement	SHELXL-2017/1 (Sheldrick, 2017)
Refinement method	Full-matrix least-squares on F^2
Reflections collected	17049
Independent reflections	2356
R_{int}	0.0883
Reflections with $I > 2\sigma(I)$	1647

Restraints	0
Parameter	92
GooF	1.062
Weighting details	$w = 1/[\sigma^2(F_{obs}^2) + (0.0341P)^2 + 4.6101P]$ where $P = (F_{obs}^2 + 2F_{calc}^2)/3$
R_1 [$I > 2\sigma(I)$]	0.0470
wR_2 [$I > 2\sigma(I)$]	0.0874
R_1 [all data]	0.0788
wR_2 [all data]	0.0968
Largest diff. peak and hole	0.906/-1.956

Comments

Treatment of hydrogen atoms

Riding model on idealized geometries with the 1.2 fold isotropic displacement parameters of the equivalent *Uij* of the corresponding carbon atom. The methyl groups are idealized with tetrahedral angles in a combined rotating and rigid group refinement with the 1.5 fold isotropic displacement parameters of the equivalent *Uij* of the corresponding carbon atom.

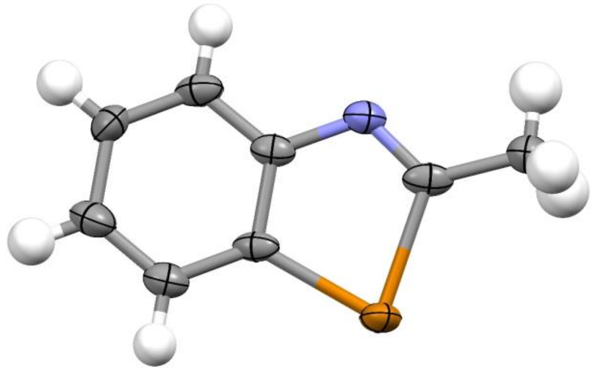

Figure S28. Molecular structure of **2** with thermal ellipsoids at 75% probability level.

Crystal Structure Data of 6

Table S9. Crystal structure data and structure refinement for **6**.

Deposition Number	2483501
Identification code	jas_117m
Empirical formula	$C_{14}H_{19}NTe$
Formula weight	328.90
Density (calculated)	1.632 g·cm^{-3}
F(000)	648
Temperature	100(2) K
Crystal size	0.274 × 0.239 × 0.038 mm
Crystal color	pale yellow
Crystal description	plate
Wavelength	1.54178 Å
Crystal system	monoclinic
Space group	$P2_1/c$
Unit cell dimensions	
a [Å]	22.3251(15)
b [Å]	5.3627(4)
c [Å]	11.2099(7)
α [°]	90
β [°]	93.9891(15)
γ [°]	90
Volume	1338.83(16) Å^3
Z	4
Cell measurement reflections used	9754
Cell measurement θ min/max	3.97°/78.91°
Diffractometer control software	Bruker APEX3(v2017.3-0)
Diffractometer measurement device	Bruker D8 Venture (Photon II detector)
Diffractometer measurement method	Data collection strategy APEX 3/Queen
θ range for data collection	3.970°- 79.243°
Completeness to θ = 67.679°	100.0%
Completeness to θ_{max} = 79.243°	99.8%
Index ranges	$-28 \leq h \leq 28$
	$-6 \leq k \leq 6$
	$-13 \leq l \leq 14$
Computing data reduction	Bruker APEX3(v2017.3-0)
Absorption coefficient	17.312 mm^{-1}
Absorption correction	Numerical
Computation absorption correction	SADABS
Max./min. Transmission	0.14/0.01
R_{merg} before/after correction	0.1310/0.0775
Computing structure solution	Bruker APEX3(v2017.3-0)
Computing structure refinement	SHELXL-2017/1 (Sheldrick, 2017)
Refinement method	Full-matrix least-squares on F^2
Reflections collected	53376
Independent reflections	2895
R_{int}	0.0584
Reflections with $I > 2\sigma(I)$	2839
Restraints	0
Parameter	146
GooF	1.112

Weighting details	$w = 1/[\sigma^2(F_{obs}^2) + (0.0333P)^2 + 4.4523P]$ where $P = (F_{obs}^2 + 2F_{calc}^2)/3$
R_1 [$I > 2\sigma(I)$]	0.0313
wR_2 [$I > 2\sigma(I)$]	0.0851
R_1 [all data]	0.0316
wR_2 [all data]	0.0852
Largest diff. peak and hole	2.192/-0.740

Comments

Treatment of hydrogen atoms

Riding model on idealized geometries with the 1.2 fold isotropic displacement parameters of the equivalent *Uij* of the corresponding carbon atom. The methyl groups are idealized with tetrahedral angles in a combined rotating and rigid group refinement with the 1.5 fold isotropic displacement parameters of the equivalent *Uij* of the corresponding carbon atom.

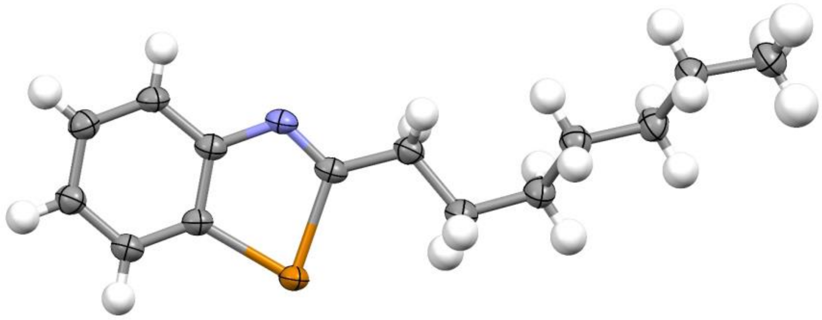

Figure S29. Molecular structure of **6** with thermal ellipsoids at 50% probability level.

Crystal Structure Data of 1a

Table S10. Crystal structure data and structure refinement for **1a**.

Deposition Number	2483497
Identification code	sm_410pym
Empirical formula	$C_8H_4N_2Te$
Formula weight	255.73
Density (calculated)	2.243 g·cm^{-3}
F(000)	944
Temperature	100(2) K
Crystal size	0.359 × 0.232 × 0.200 mm
Crystal color	pale brown
Crystal description	block
Wavelength	0.71073 Å
Crystal system	monoclinic
Space group	*C*2/*c*
Unit cell dimensions	
a [Å]	29.041(3)
b [Å]	4.8698(6)
c [Å]	11.1376(13)
α [°]	90
β [°]	105.953(3)
γ [°]	90
Volume	1514.4(3) Å^3
Z	8
Cell measurement reflections used	9917
Cell measurement θ min/max	2.92°/36.34°
Diffractometer control software	BRUKER APEX3(v2019.1-0)
Diffractometer measurement device	Bruker D8 KAPPA II (APEX II detector)
Diffractometer measurement method	Data collection strategy APEX 2/COSMO
θ range for data collection	2.918°- 36.352°
Completeness to θ = 25.242°	99.7%
Completeness to θ_{max} = 36.352°	99.7%
Index ranges	$-48 \leq h \leq 48$
	$-8 \leq k \leq 8$
	$-18 \leq l \leq 18$
Computing data reduction	BRUKER APEX3(v2019.1-0)
Absorption coefficient	3.853 mm^{-1}
Absorption correction	Semi-empirical from equivalents
Computation absorption correction	SADABS
Max./min. Transmission	0.75/0.51
R_{merg} before/after correction	0.0881/0.0417
Computing structure solution	BRUKER APEX3(v2019.1-0)
Computing structure refinement	SHELXL-2017/1 (Sheldrick, 2017)
Refinement method	Full-matrix least-squares on F^2
Reflections collected	34931
Independent reflections	3676
R_{int}	0.0239
Reflections with $I > 2\sigma(I)$	3618
Restraints	0
Parameter	100
GooF	1.357

Weighting details	$w = 1/[\sigma^2(F_{obs}^2) + (0.0140P)^2 + 3.7737P]$ where $P = (F_{obs}^2 + 2F_{calc}^2)/3$
R_1 [$I > 2\sigma(I)$]	0.0196
wR_2 [$I > 2\sigma(I)$]	0.0489
R_1 [all data]	0.0200
wR_2 [all data]	0.0490
Largest diff. peak and hole	1.723/-1.224

Comments

Treatment of hydrogen atoms

Riding model on idealized geometries with the 1.2 fold isotropic displacement parameters of the equivalent *Uij* of the corresponding carbon atom.

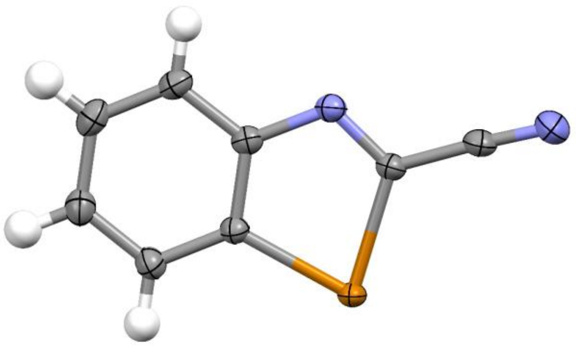

Figure S30. Molecular structure of **1a** with thermal ellipsoids at 75% probability level.

Crystal Structure Data of 1b

Table S11. Crystal structure data and structure refinement for **1b**.

Deposition Number	2483498
Identification code	jas_214m
Empirical formula	$C_8H_3FN_2Te$
Formula weight	273.72
Density (calculated)	2.390 g·cm^{-3}
F(000)	1008
Temperature	100(2) K
Crystal size	0.282 × 0.105 × 0.057 mm
Crystal color	pale yellow
Crystal description	tablet
Wavelength	0.71073 Å
Crystal system	monoclinic
Space group	*C*2/*c*
Unit cell dimensions	
a [Å]	28.453(2)
b [Å]	4.8332(4)
c [Å]	11.0718(9)
α [°]	90
β [°]	92.221(3)
γ [°]	90
Volume	1521.5(2) Å^3
Z	8
Cell measurement reflections used	9763
Cell measurement θ min/max	3.90°/40.52°
Diffractometer control software	BRUKER APEX3(v2019.1-0)
Diffractometer measurement device	Bruker D8 KAPPA II (APEX II detector)
Diffractometer measurement method	Data collection strategy APEX 3/Queen
θ range for data collection	2.866°- 40.593°
Completeness to θ = 25.242°	99.9%
Completeness to θ_{max} = 40.593°	99.6%
Index ranges	$-51 \leq h \leq 51$
	$-8 \leq k \leq 8$
	$-20 \leq l \leq 20$
Computing data reduction	BRUKER APEX3(v2019.1-0)
Absorption coefficient	3.862 mm^{-1}
Absorption correction	Semi-empirical from equivalents
Computation absorption correction	SADABS
Max./min. Transmission	0.75/0.56
R_{merg} before/after correction	0.0711/0.0407
Computing structure solution	BRUKER APEX3(v2019.1-0)
Computing structure refinement	SHELXL-2017/1 (Sheldrick, 2017)
Refinement method	Full-matrix least-squares on F^2
Reflections collected	82198
Independent reflections	4871
R_{int}	0.0277
Reflections with $I > 2\sigma(I)$	4599
Restraints	0
Parameter	109
GooF	1.076

Weighting details	$w = 1/[\sigma^2(F_{obs}^2) + (0.0146P)^2 + 1.1515P]$ where $P = (F_{obs}^2 + 2F_{calc}^2)/3$
R_1 [$I > 2\sigma(I)$]	0.0121
wR_2 [$I > 2\sigma(I)$]	0.0305
R_1 [all data]	0.0134
wR_2 [all data]	0.0310
Largest diff. peak and hole	0.681/-0.498

Comments

Treatment of hydrogen atoms

Riding model on idealized geometries with the 1.2 fold isotropic displacement parameters of the equivalent *Uij* of the corresponding carbon atom.

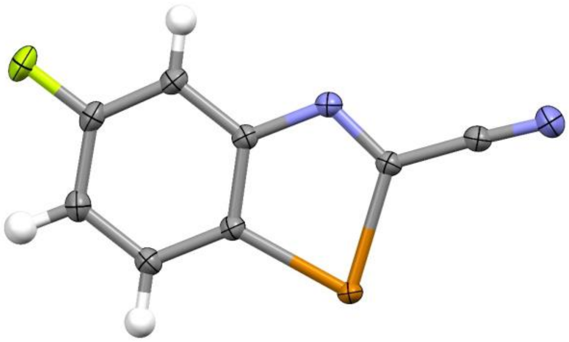

Figure S31. Molecular structure of **1b** with thermal ellipsoids at 75% probability level.

Crystal Structure Data of 1c

Table S12. Crystal structure data and structure refinement for **1c**.

Deposition Number	2483499
Identification code	jas_209m
Empirical formula	$C_8H_2F_2N_2Te$
Formula weight	291.72
Density (calculated)	2.482 g·cm^{-3}
F(000)	1072
Temperature	100(2) K
Crystal size	0.242 × 0.084 × 0.061 mm
Crystal color	pale yellow
Crystal description	tablet
Wavelength	0.71073 Å
Crystal system	monoclinic
Space group	*C*2/*c*
Unit cell dimensions	
a [Å]	30.6995(16)
b [Å]	4.7346(3)
c [Å]	11.1438(6)
α [°]	90
β [°]	105.443(2)
γ [°]	90
Volume	1561.27(15) Å^3
Z	8
Cell measurement reflections used	9986
Cell measurement θ min/max	2.75°/40.29°
Diffractometer control software	BRUKER APEX3(v2019.1-0)
Diffractometer measurement device	Bruker D8 KAPPA II (APEX II detector)
Diffractometer measurement method	Data collection strategy APEX 3/Queen
θ range for data collection	2.753°- 40.476°
Completeness to θ = 25.242°	99.9%
Completeness to θ_{max} = 40.476°	99.8%
Index ranges	$-56 \leq h \leq 55$
	$-8 \leq k \leq 8$
	$-20 \leq l \leq 19$
Computing data reduction	BRUKER APEX3(v2019.1-0)
Absorption coefficient	3.789 mm^{-1}
Absorption correction	Semi-empirical from equivalents
Computation absorption correction	SADABS
Max./min. Transmission	0.75/0.56
R_{merg} before/after correction	0.0817/0.0511
Computing structure solution	BRUKER APEX3(v2019.1-0)
Computing structure refinement	SHELXL-2017/1 (Sheldrick, 2017)
Refinement method	Full-matrix least-squares on F^2
Reflections collected	50471
Independent reflections	4976
R_{int}	0.0443
Reflections with $I > 2\sigma(I)$	4296
Restraints	0
Parameter	118
GooF	1.035

Weighting details	$w = 1/[\sigma^2(F_{obs}^2) + (0.0168P)^2 + 1.7653P]$ where $P = (F_{obs}^2 + 2F_{calc}^2)/3$
R_1 [$I > 2\sigma(I)$]	0.0196
wR_2 [$I > 2\sigma(I)$]	0.0411
R_1 [all data]	0.0268
wR_2 [all data]	0.0431
Largest diff. peak and hole	0.893/-0.847

Comments

Treatment of hydrogen atoms

Riding model on idealized geometries with the 1.2 fold isotropic displacement parameters of the equivalent *Uij* of the corresponding carbon atom.

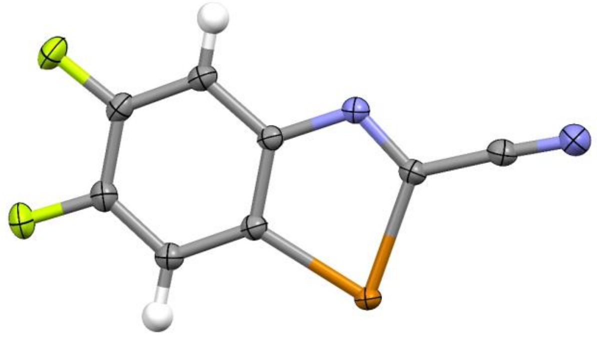

Figure S32. Molecular structure of **1c** with thermal ellipsoids at 75% probability level.

Crystal Structure Data of 7b

Table S13. Crystal structure data and structure refinement for **7b**.

Deposition Number	2483502
Identification code	jas_199_tw4
Empirical formula	$C_{10}H_8FNO_2Te$
Formula weight	320.77
Density (calculated)	2.063 g·cm^{-3}
F(000)	608
Temperature	100(2) K
Crystal size	0.195 × 0.086 × 0.048 mm
Crystal color	pale yellow
Crystal description	tablet
Wavelength	1.54178 Å
Crystal system	monoclinic
Space group	$P2_1/n$
Unit cell dimensions	
a [Å]	9.6880(6)
b [Å]	7.3158(5)
c [Å]	15.3467(10)
α [°]	90
β [°]	108.287(3)
γ [°]	90
Volume	1032.77(12) Å^3
Z	4
Cell measurement reflections used	9962
Cell measurement θ min/max	4.81°/78.97°
Diffractometer control software	Bruker APEX3(v2017.3-0)
Diffractometer measurement device	Bruker D8 Venture (Photon II detector)
Diffractometer measurement method	Data collection strategy APEX 3/Queen
θ range for data collection	4.813°- 79.599°
Completeness to θ = 67.679°	99.9%
Completeness to θ_{max} = 79.599°	99.4%
Index ranges	$-12 \leq h \leq 11$
	$0 \leq k \leq 9$
	$0 \leq l \leq 19$
Computing data reduction	Bruker APEX3(v2017.3-0)
Absorption coefficient	22.728 mm^{-1}
Absorption correction	Semi-empirical from equivalents
Computation absorption correction	TWINABS
Max./min. Transmission	0.18/0.03
R_{merg} before/after correction	0.1645/0.0878 and 0.1581/0.0908
Computing structure solution	Bruker APEX3(v2017.3-0)
Computing structure refinement	SHELXL-2017/1 (Sheldrick, 2017)
Refinement method	Full-matrix least-squares on F^2
Reflections collected	73037
Independent reflections	2244
R_{int}	0.0923
Reflections with $I > 2\sigma(I)$	2078
Restraints	0
Parameter	137
GooF	1.097

Weighting details	$w = 1/[\sigma^2(F_{obs}^2) + (0.0873P)^2 + 2.3337P]$ where $P = (F_{obs}^2 + 2F_{calc}^2)/3$
R_1 [$I > 2\sigma(I)$]	0.0513
wR_2 [$I > 2\sigma(I)$]	0.1305
R_1 [all data]	0.0536
wR_2 [all data]	0.1331
Largest diff. peak and hole	4.188/-0.754

Comments

Treatment of hydrogen atoms

Riding model on idealized geometries with the 1.2 fold isotropic displacement parameters of the equivalent *Uij* of the corresponding carbon atom. The methyl groups are idealized with tetrahedral angles in a combined rotating and rigid group refinement with the 1.5 fold isotropic displacement parameters of the equivalent *Uij* of the corresponding carbon atom.

Twinning

The crystal was a non-merohedral twin of several domains of which the two largest could be successfully integrated. The model was refined against de-twinned hklf4 data. Considering the domains that could not be integrated the quantitative results should be carefully assessed.

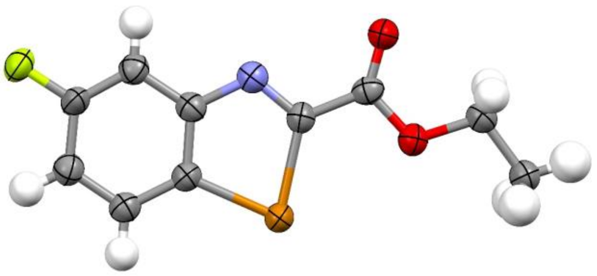

Figure S33. Molecular structure of **7b** with thermal ellipsoids at 50% probability level.

Crystal Structure Data of 7c

Table S14. Crystal structure data and structure refinement for **7c**.

Deposition Number	2483503
Identification code	jas_200m
Empirical formula	$C_{10}H_7F_2NO_2Te$
Formula weight	338.77
Density (calculated)	2.145 g·cm^{-3}
F(000)	640
Temperature	100(2) K
Crystal size	0.189 × 0.123 × 0.049 mm
Crystal color	pale yellow
Crystal description	plate
Wavelength	1.54178 Å
Crystal system	monoclinic
Space group	$P2_1/c$
Unit cell dimensions	
a [Å]	7.2043(7)
b [Å]	6.8196(6)
c [Å]	21.542(2)
α [°]	90
β [°]	97.624(2)
γ [°]	90
Volume	1048.99(17) Å^3
Z	4
Cell measurement reflections used	9686
Cell measurement θ min/max	4.14°/79.60°
Diffractometer control software	Bruker APEX3(v2017.3-0)
Diffractometer measurement device	Bruker D8 Venture (Photon II detector)
Diffractometer measurement method	Data collection strategy APEX 3/Queen
θ range for data collection	4.141°- 79.801°
Completeness to θ = 67.679°	100.0%
Completeness to θ_{max} = 79.801°	99.9%
Index ranges	$-9 \le h \le 9$
	$-8 \le k \le 7$
	$-27 \le l \le 27$
Computing data reduction	Bruker APEX3(v2017.3-0)
Absorption coefficient	22.567 mm^{-1}
Absorption correction	Semi-empirical from equivalents
Computation absorption correction	SADABS
Max./min. Transmission	0.75/0.30
R_{merg} before/after correction	0.1424/0.0779
Computing structure solution	Bruker APEX3(v2017.3-0)
Computing structure refinement	SHELXL-2017/1 (Sheldrick, 2017)
Refinement method	Full-matrix least-squares on F^2
Reflections collected	47410
Independent reflections	2291
R_{int}	0.0566
Reflections with $I > 2\sigma(I)$	2285
Restraints	0
Parameter	146
GooF	1.093

Weighting details	$w = 1/[\sigma^2(F_{obs}^2) + (0.0251P)^2 + 1.4574P]$ where $P = (F_{obs}^2 + 2F_{calc}^2)/3$
R_1 [$I > 2\sigma(I)$]	0.0192
wR_2 [$I > 2\sigma(I)$]	0.0502
R_1 [all data]	0.0193
wR_2 [all data]	0.0503
Largest diff. peak and hole	0.691/-0.511

Comments

Treatment of hydrogen atoms

Riding model on idealized geometries with the 1.2 fold isotropic displacement parameters of the equivalent *Uij* of the corresponding carbon atom. The methyl groups are idealized with tetrahedral angles in a combined rotating and rigid group refinement with the 1.5 fold isotropic displacement parameters of the equivalent *Uij* of the corresponding carbon atom.

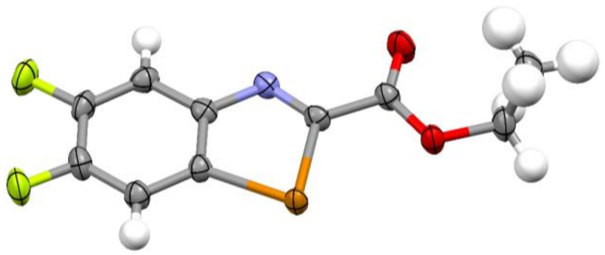

Figure S34. Molecular structure of **7c** with thermal ellipsoids at 75% probability level.

Crystal Structure Data of 8b

Table S15. Crystal structure data and structure refinement for **8b**.

Deposition Number	2483504
Identification code	jas_207m
Empirical formula	$C_8H_5FN_2OTe$
Formula weight	291.74
Density (calculated)	2.270 g·cm^{-3}
F(000)	544
Temperature	100(2) K
Crystal size	0.425 × 0.102 × 0.032 mm
Crystal color	pale yellow
Crystal description	plate
Wavelength	1.54178 Å
Crystal system	monoclinic
Space group	$P2_1/c$
Unit cell dimensions	
a [Å]	5.0904(6)
b [Å]	11.1361(14)
c [Å]	15.0966(18)
α [°]	90
β [°]	94.154(3)
γ [°]	90
Volume	853.54(18) Å^3
Z	4
Cell measurement reflections used	9087
Cell measurement θ min/max	4.94°/79.21°
Diffractometer control software	Bruker APEX3(v2017.3-0)
Diffractometer measurement device	Bruker D8 Venture (Photon II detector)
Diffractometer measurement method	Data collection strategy APEX 3/Queen
θ range for data collection	4.939°- 79.200°
Completeness to θ = 67.679°	99.9%
Completeness to θ_{max} = 79.200°	99.7%
Index ranges	$-5 \le h \le 6$
	$-14 \le k \le 14$
	$-19 \le l \le 19$
Computing data reduction	Bruker APEX3(v2017.3-0)
Absorption coefficient	27.355 mm^{-1}
Absorption correction	Semi-empirical from equivalents
Computation absorption correction	SADABS
Max./min. Transmission	0.75/0.24
R_{merg} before/after correction	0.1574/0.0902
Computing structure solution	Bruker APEX3(v2017.3-0)
Computing structure refinement	SHELXL-2017/1 (Sheldrick, 2017)
Refinement method	Full-matrix least-squares on F^2
Reflections collected	27916
Independent reflections	1848
R_{int}	0.0615
Reflections with $I > 2\sigma(I)$	1609
Restraints	0
Parameter	126
GooF	1.150

Weighting details	$w = 1/[\sigma^2(F_{obs}^2) + (0.0591P)^2 + 1.9350P]$ where $P = (F_{obs}^2 + 2F_{calc}^2)/3$
R_1 [$I > 2\sigma(I)$]	0.0373
wR_2 [$I > 2\sigma(I)$]	0.1025
R_1 [all data]	0.0410
wR_2 [all data]	0.1080
Largest diff. peak and hole	1.774/-0.950

Comments

Treatment of hydrogen atoms

Riding model on idealized geometries with the 1.2 fold isotropic displacement parameters of the equivalent *Uij* of the corresponding carbon atom. The NH hydrogen atoms were refined freely.

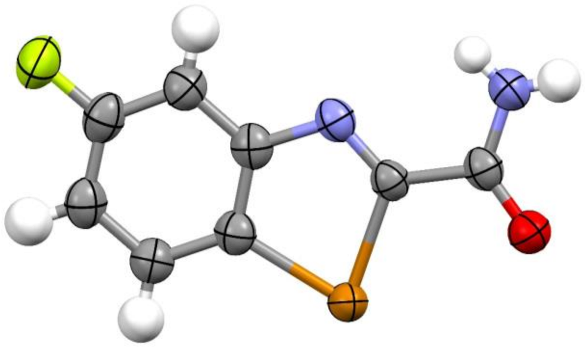

Figure S35. Molecular structure of **8b** with thermal ellipsoids at 75% probability level.

Crystal Structure Data of 8c

Table S16. Crystal structure data and structure refinement for **8c**.

Deposition Number	2483505
Identification code	jas_211m
Empirical formula	$C_8H_4F_2N_2OTe$
Formula weight	309.73
Density (calculated)	2.394 g·cm^{-3}
F(000)	1728
Temperature	100(2) K
Crystal size	0.301 × 0.174 × 0.158 mm
Crystal color	yellow
Crystal description	block
Wavelength	0.71073 Å
Crystal system	monoclinic
Space group	$P2_1/c$
Unit cell dimensions	
a [Å]	14.3667(5)
b [Å]	16.1520(6)
c [Å]	11.1475(4)
α [°]	90
β [°]	94.7333(15)
γ [°]	90
Volume	2577.97(16) Å^3
Z	12
Cell measurement reflections used	9860
Cell measurement *θ* min/max	2.85°/45.56°
Diffractometer control software	BRUKER APEX3(v2019.1-0)
Diffractometer measurement device	Bruker D8 KAPPA II (APEX II detector)
Diffractometer measurement method	Data collection strategy APEX 3/Queen
θ range for data collection	2.225°- 45.666°
Completeness to *θ* = 25.242°	99.9%
Completeness to θ_{max} = 45.666°	99.6%
Index ranges	$-28 \leq h \leq 28$
	$-32 \leq k \leq 32$
	$-22 \leq l \leq 22$
Computing data reduction	BRUKER APEX3(v2019.1-0)
Absorption coefficient	3.458 mm^{-1}
Absorption correction	Numerical
Computation absorption correction	SADABS
Max./min. Transmission	0.19/0.10
R_{merg} before/after correction	0.0610/0.0479
Computing structure solution	BRUKER APEX3(v2019.1-0)
Computing structure refinement	SHELXL-2017/1 (Sheldrick, 2017)
Refinement method	Full-matrix least-squares on F^2
Reflections collected	230925
Independent reflections	21949
R_{int}	0.0340
Reflections with $I > 2\sigma(I)$	18662
Restraints	0
Parameter	403
GooF	1.080

Weighting details	$w = 1/[\sigma^2(F_{obs}^2) + (0.0217P)^2 + 0.9163P]$ where $P = (F_{obs}^2 + 2F_{calc}^2)/3$
R_1 [$I > 2\sigma(I)$]	0.0207
wR_2 [$I > 2\sigma(I)$]	0.0483
R_1 [all data]	0.0287
wR_2 [all data]	0.0517
Largest diff. peak and hole	0.647/-1.889

Comments

Treatment of hydrogen atoms

Riding model on idealized geometries with the 1.2 fold isotropic displacement parameters of the equivalent *Uij* of the corresponding carbon atom. The NH hydrogen atoms were refined freely.

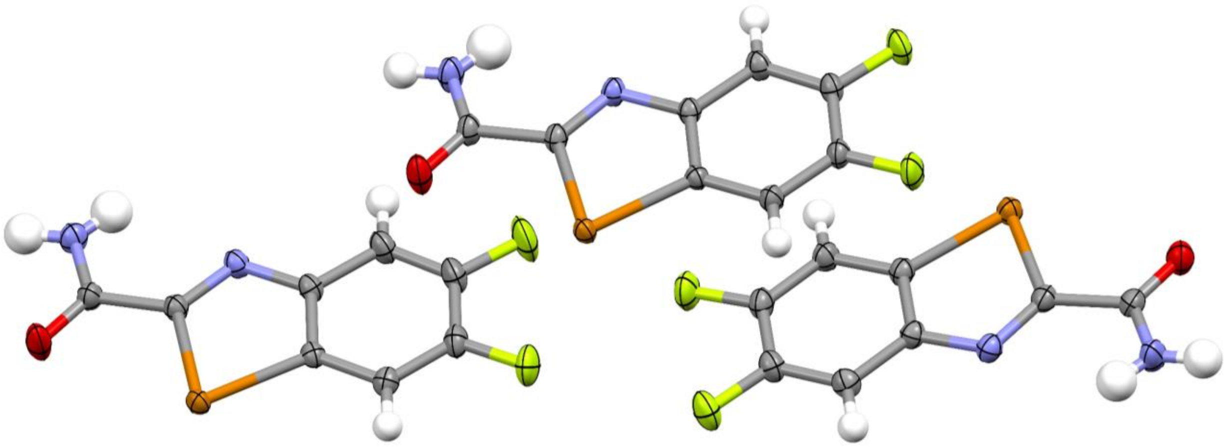

Figure S36. Molecular structure of **8c** with thermal ellipsoids at 75% probability level.

5. ^{1}H NMR, ^{13}C NMR and ^{125}Te NMR Spectra of the New Compounds

The field strengths and solvents of the following spectra are given in the Experimental Section.

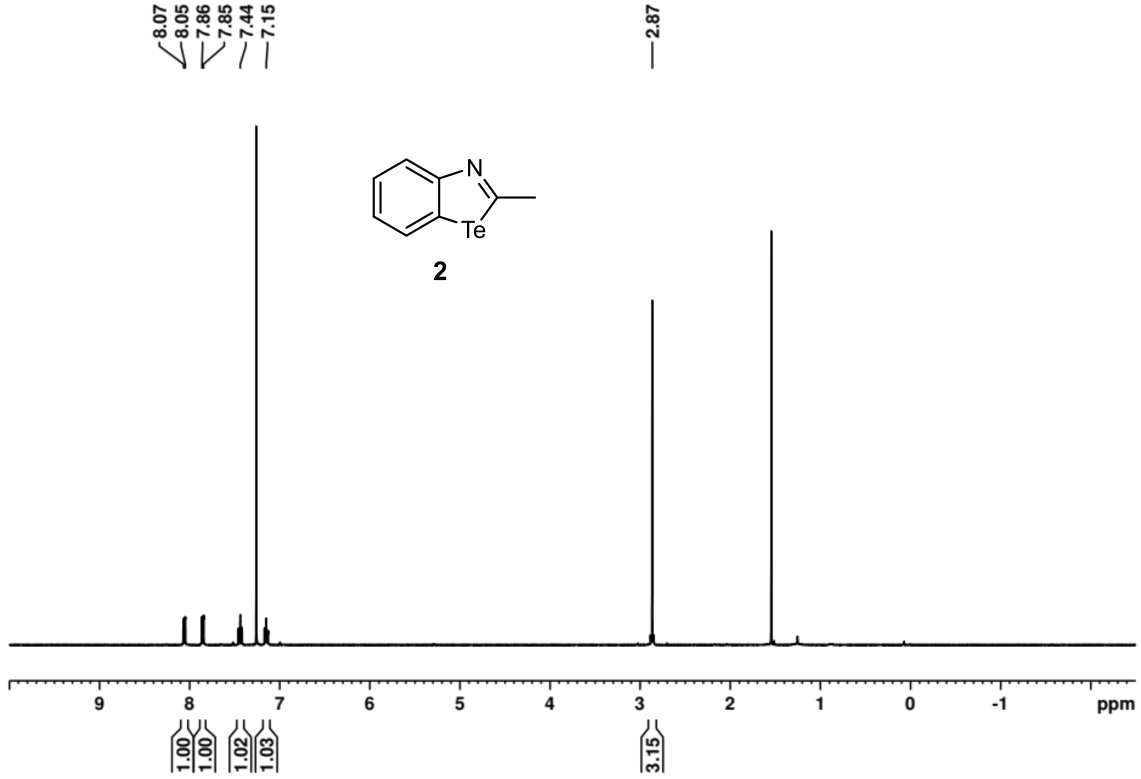

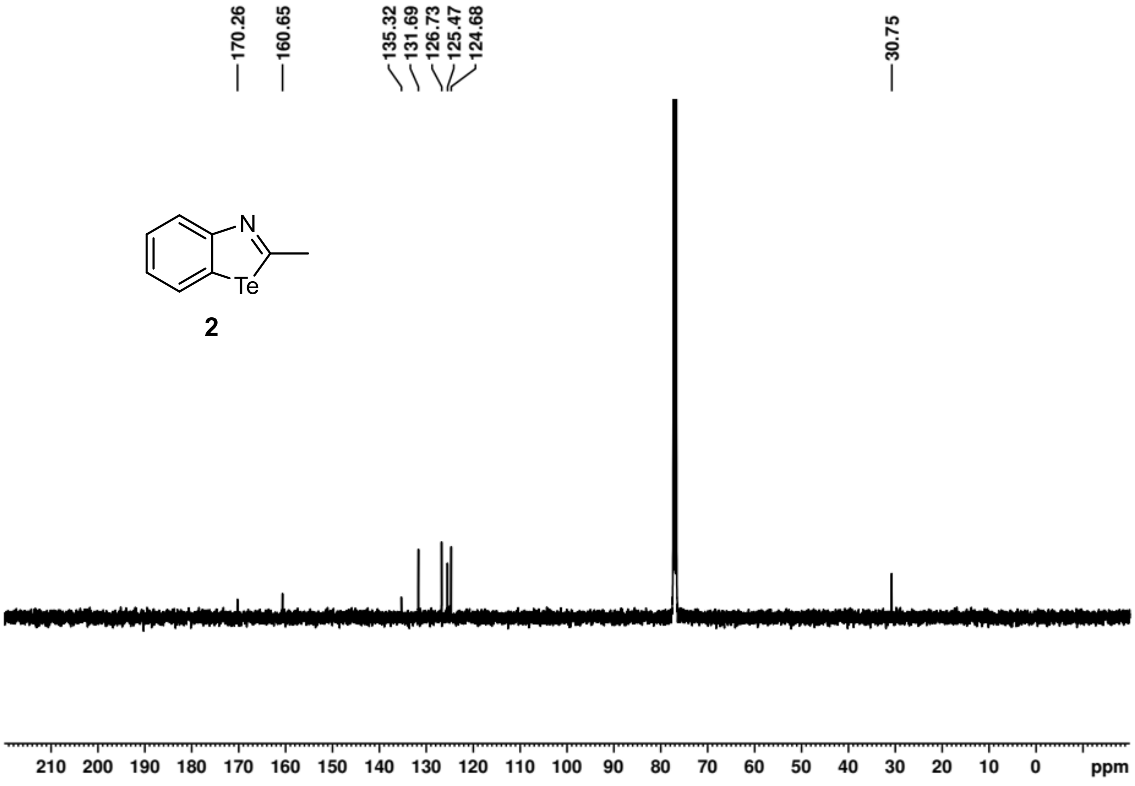

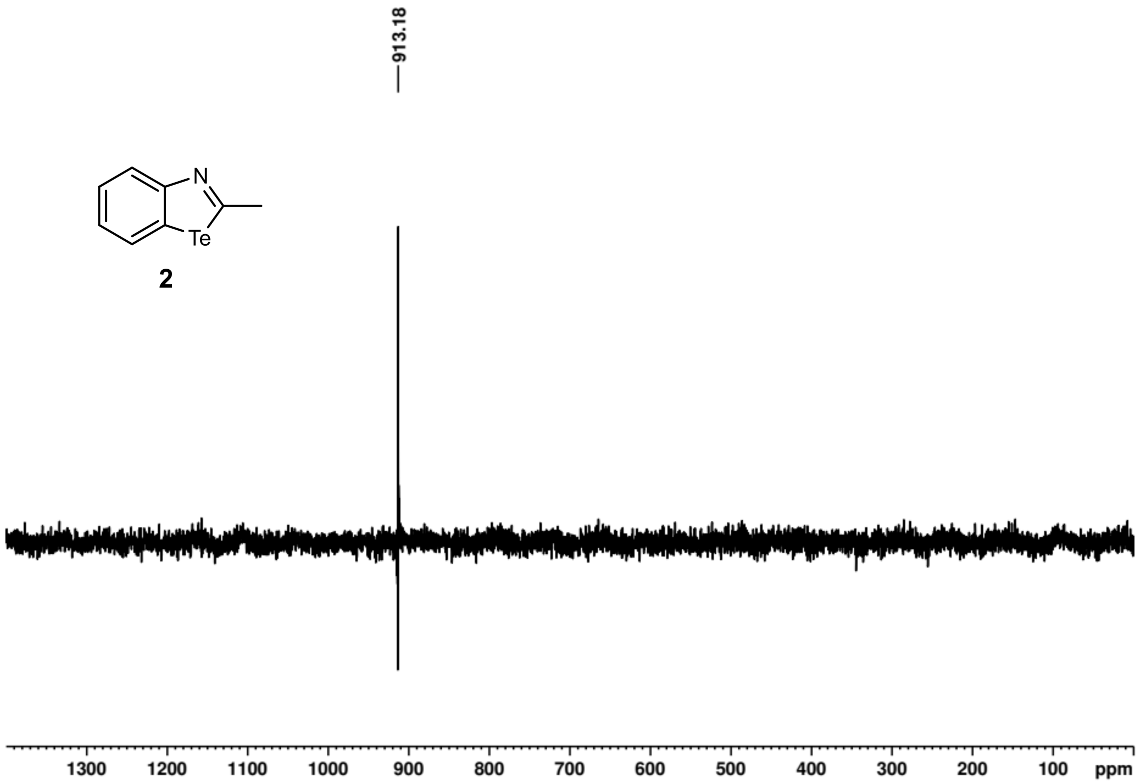
913.18
N
Te
2
1300
1200
1100
1000
900
800
700
600
500
400
300
200
100
ppm

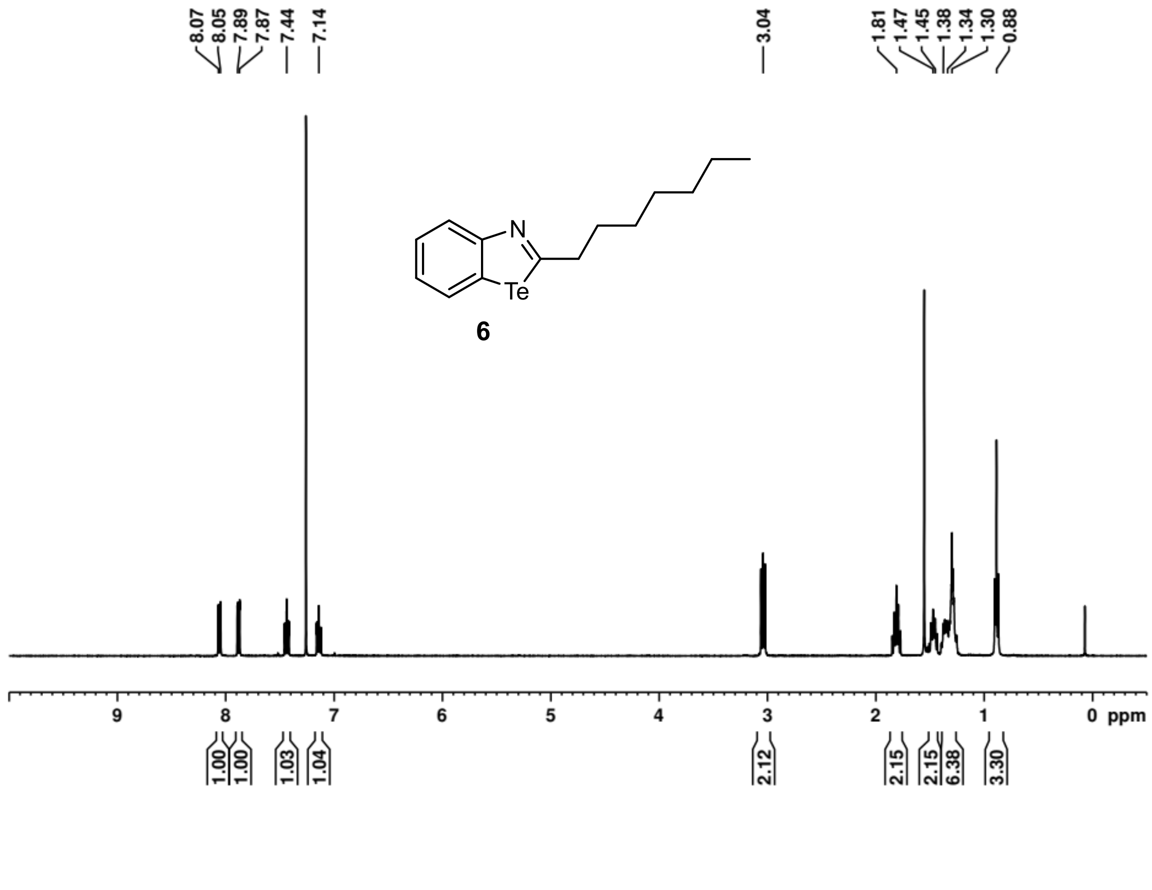
8.07
8.05
7.89
7.87
7.44
7.14
3.04
1.81
1.47
1.45
1.38
1.34
1.30
0.88
N
Te
6
9
8
7
6
5
4
3
2
1
0
ppm
1.00
1.00
1.03
1.04
2.12
2.15
2.15
6.38
3.30

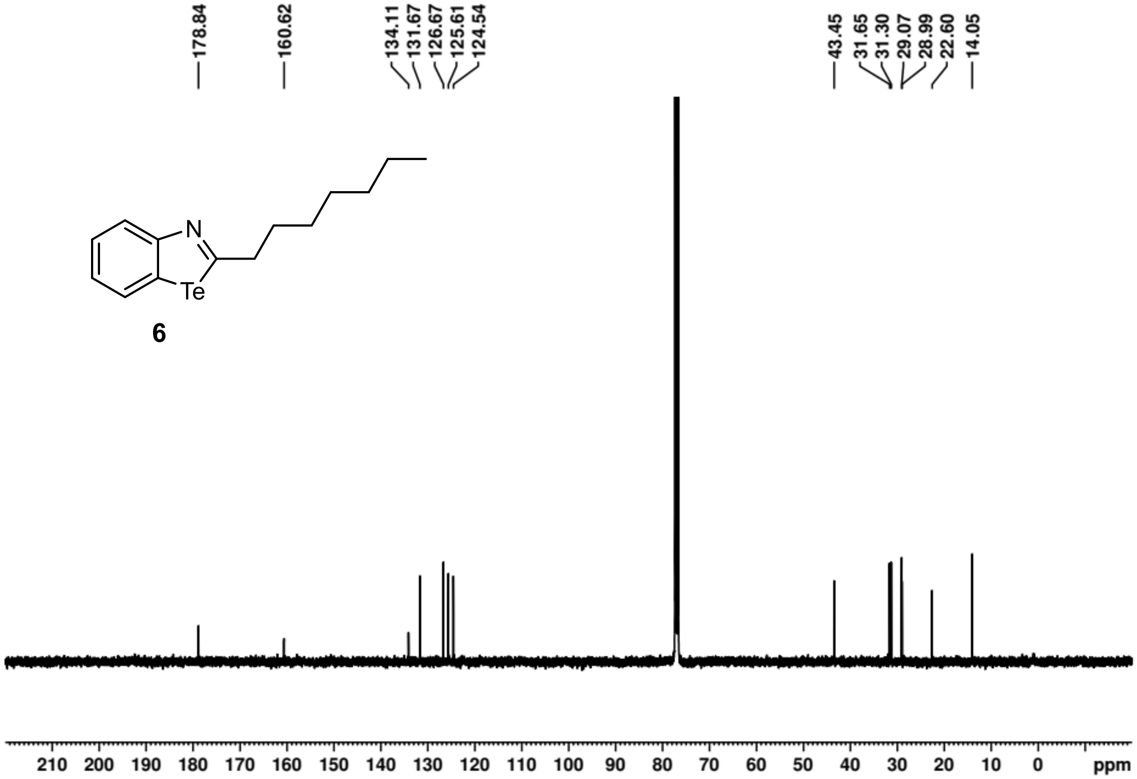
178.84
160.62
134.11
131.67
126.67
125.61
124.54
43.45
31.65
31.30
29.07
28.99
22.60
14.05
N
Te
6
210 200 190 180 170 160 150 140 130 120 110 100 90 80 70 60 50 40 30 20 10 0 ppm

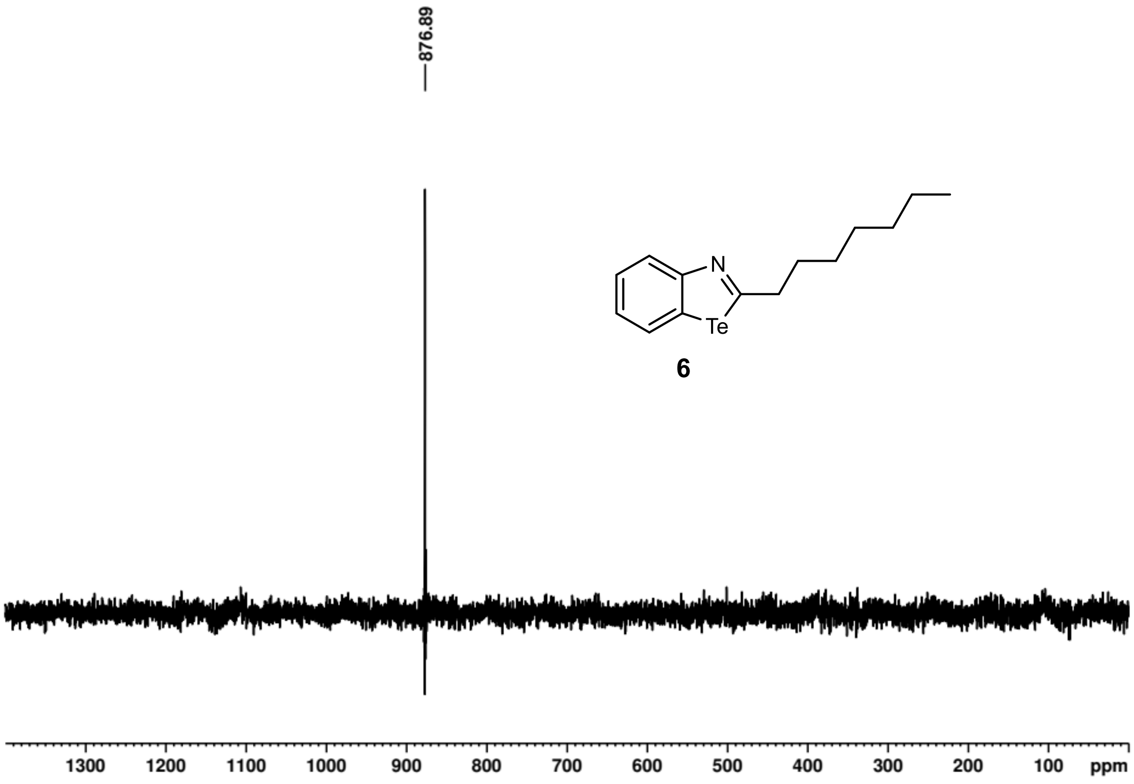
876.89
N
Te
6
1300 1200 1100 1000 900 800 700 600 500 400 300 200 100 ppm

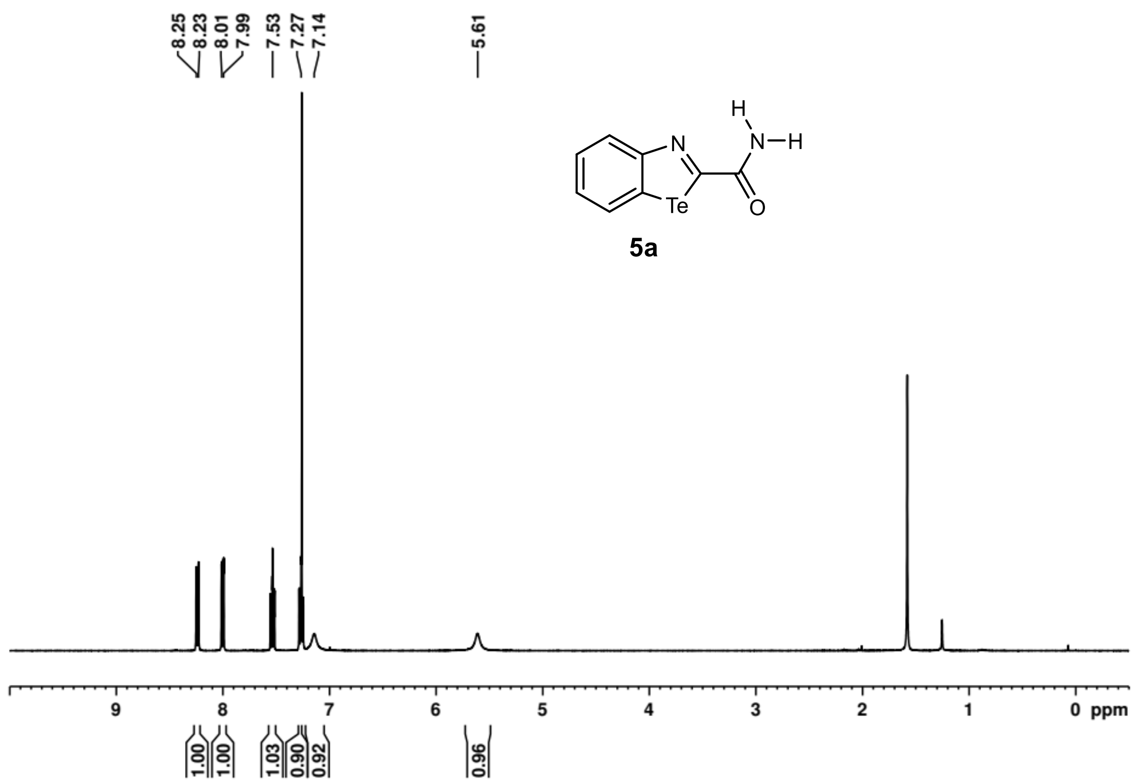
8.25
8.23
8.01
7.99
7.53
7.27
7.14
5.61
H
N
N–H
Te
O
5a
9
8
7
6
5
4
3
2
1
0 ppm
1.00
1.00
1.03
0.90
0.92
0.96

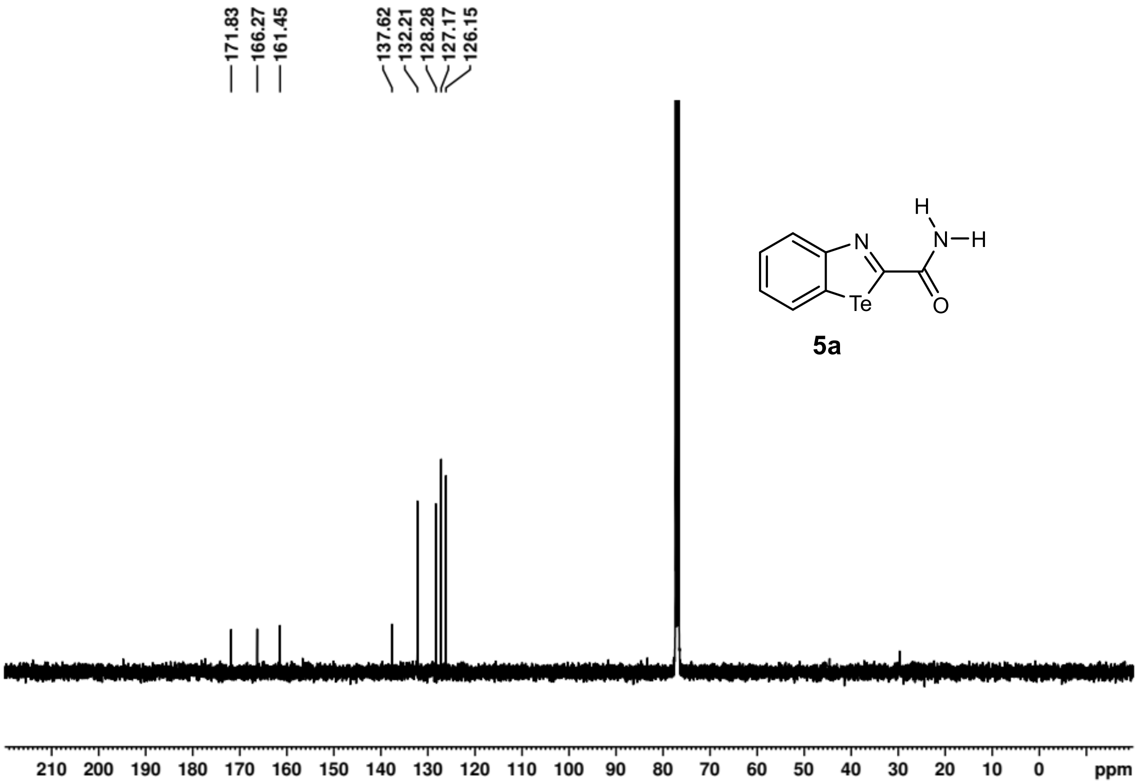
171.83
166.27
161.45
137.62
132.21
128.28
127.17
126.15
H
N
N–H
Te
O
5a
210 200 190 180 170 160 150 140 130 120 110 100 90 80 70 60 50 40 30 20 10 0
ppm

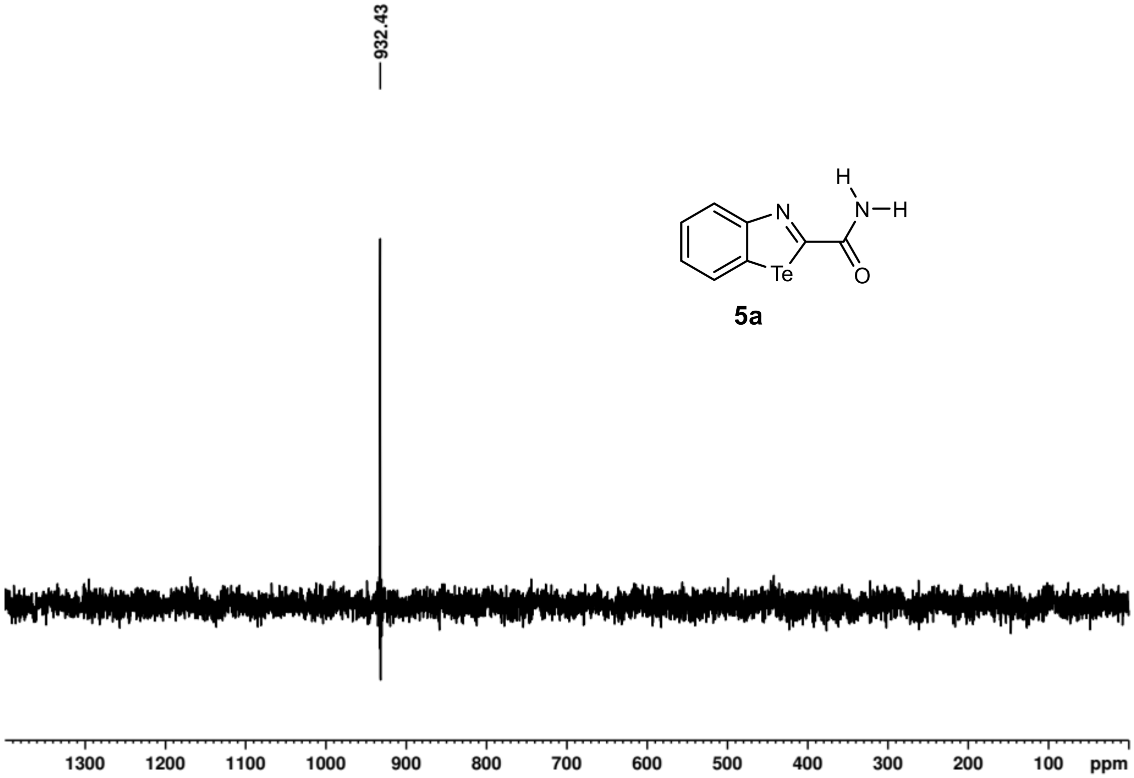

932.43
N
N
H
H
O
Te
5a
1300
1200
1100
1000
900
800
700
600
500
400
300
200
100
ppm

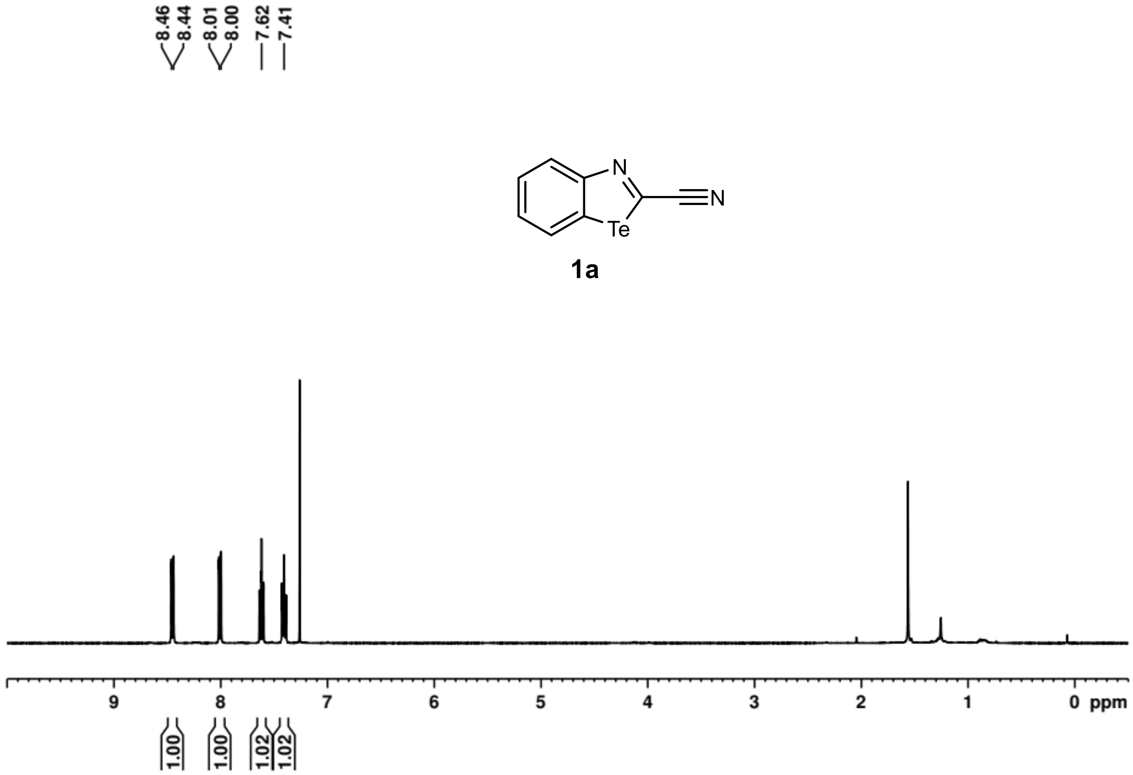

8.46
8.44
8.01
8.00
7.62
7.41
N
Te
N
1a
9
8
7
6
5
4
3
2
1
0
ppm
1.00
1.00
1.02
1.02

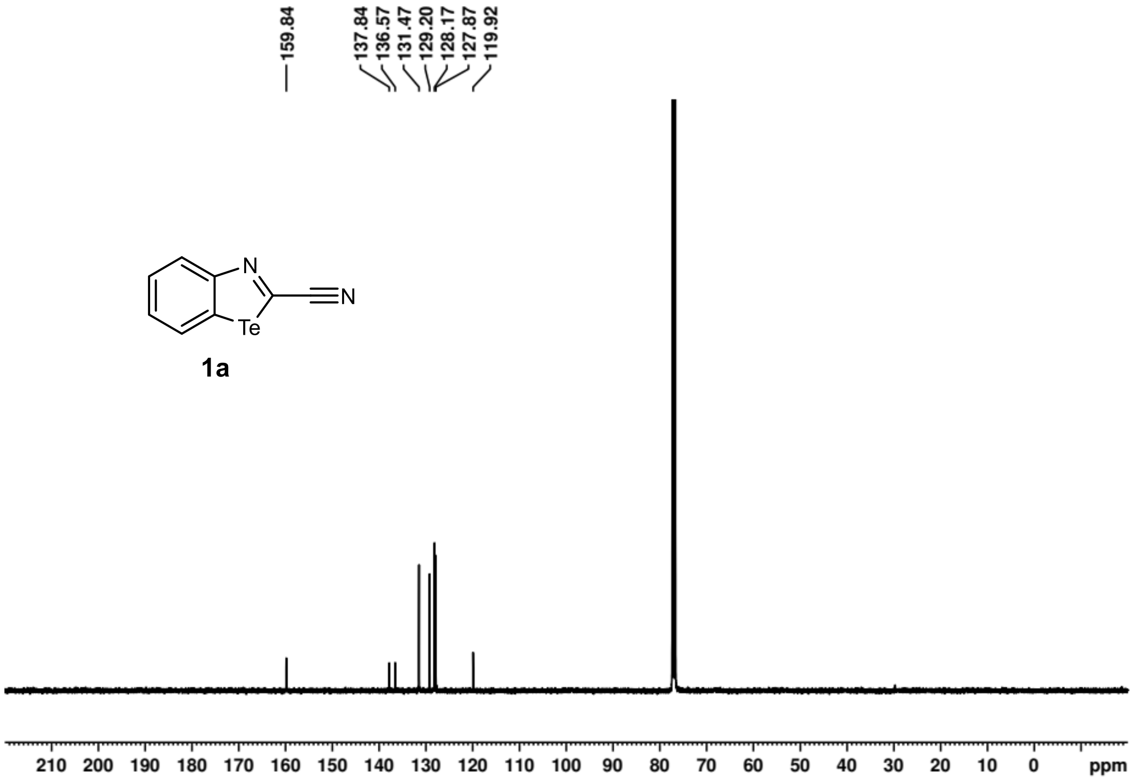
159.84
137.84
136.57
131.47
129.20
128.17
127.87
119.92
N
Te
N
1a
210 200 190 180 170 160 150 140 130 120 110 100 90 80 70 60 50 40 30 20 10 0 ppm

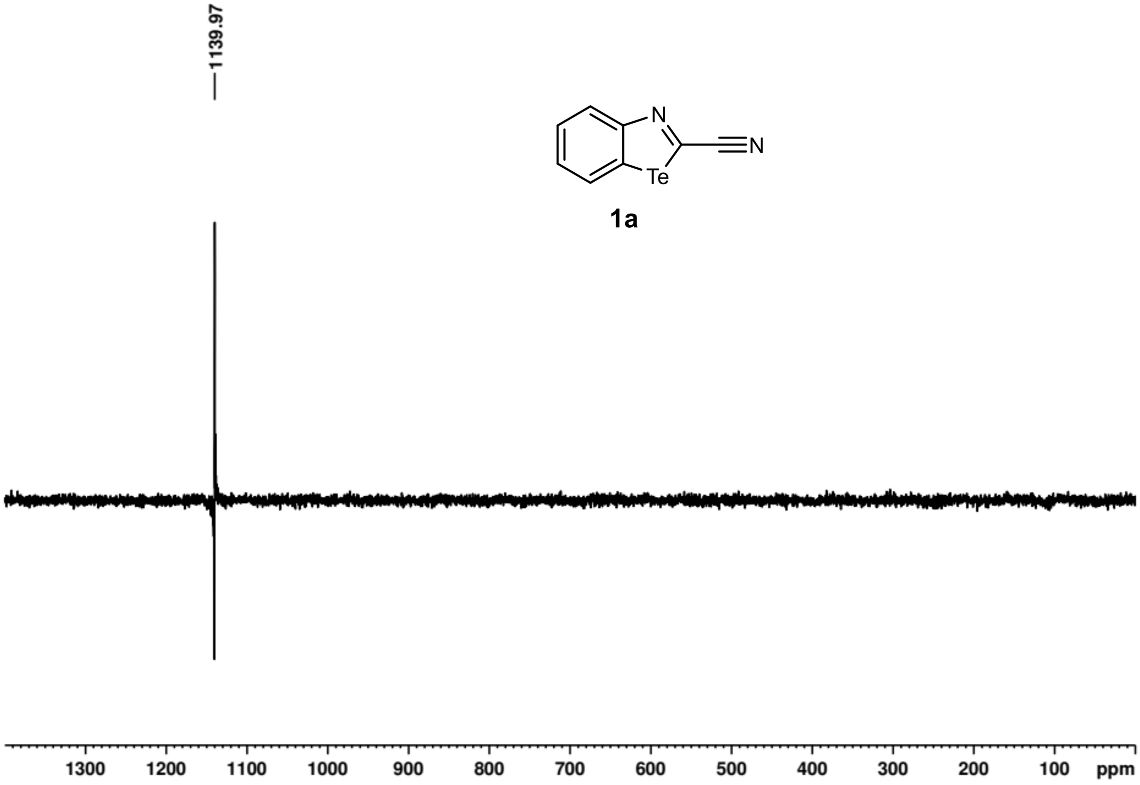
1139.97
N
Te
N
1a
1300 1200 1100 1000 900 800 700 600 500 400 300 200 100 ppm

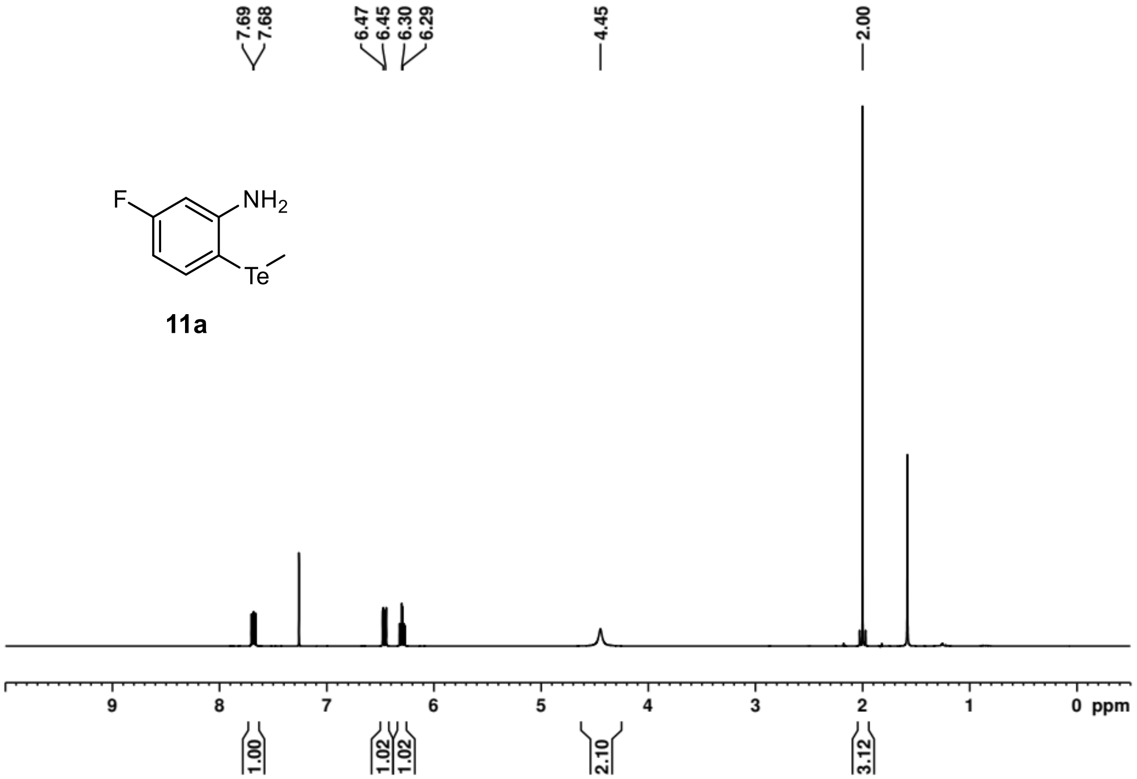

7.69
7.68
6.47
6.45
6.30
6.29
4.45
2.00
F
NH2
Te
11a
9
8
7
6
5
4
3
2
1
0 ppm
1.00
1.02
1.02
2.10
3.12

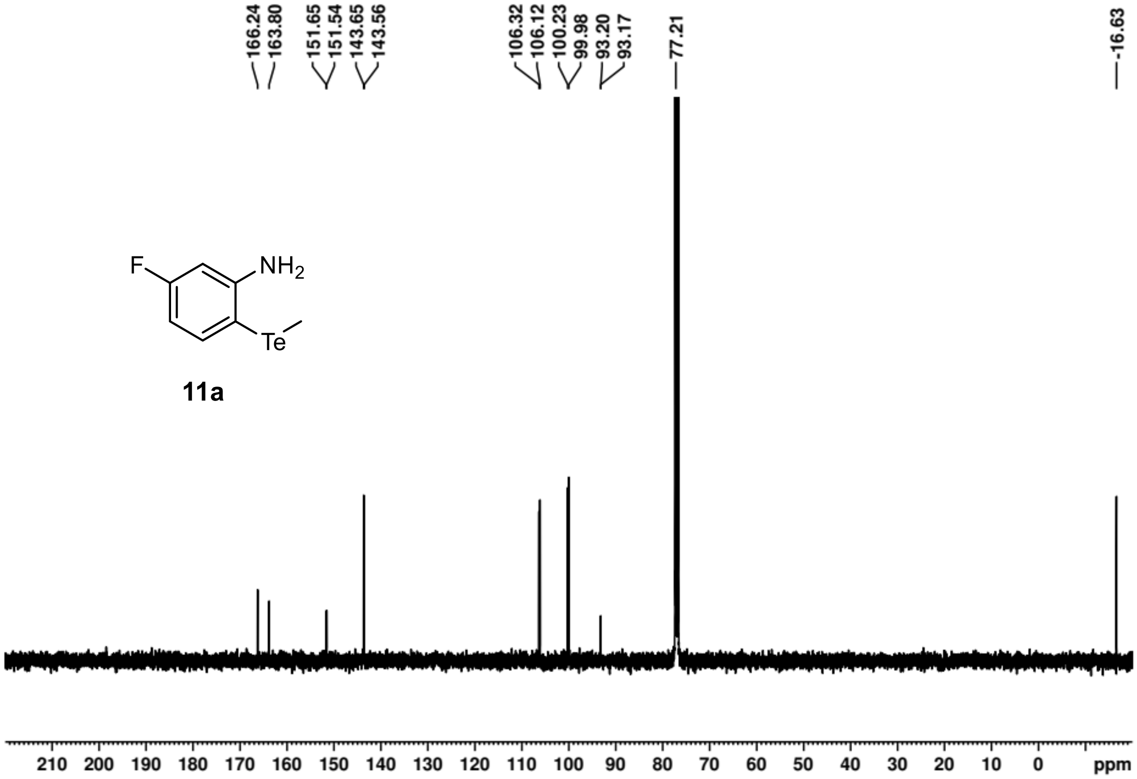

166.24
163.80
151.65
151.54
143.65
143.56
106.32
106.12
100.23
99.98
93.20
93.17
77.21
-16.63
F
NH2
Te
11a
210 200 190 180 170 160 150 140 130 120 110 100 90 80 70 60 50 40 30 20 10 0 ppm

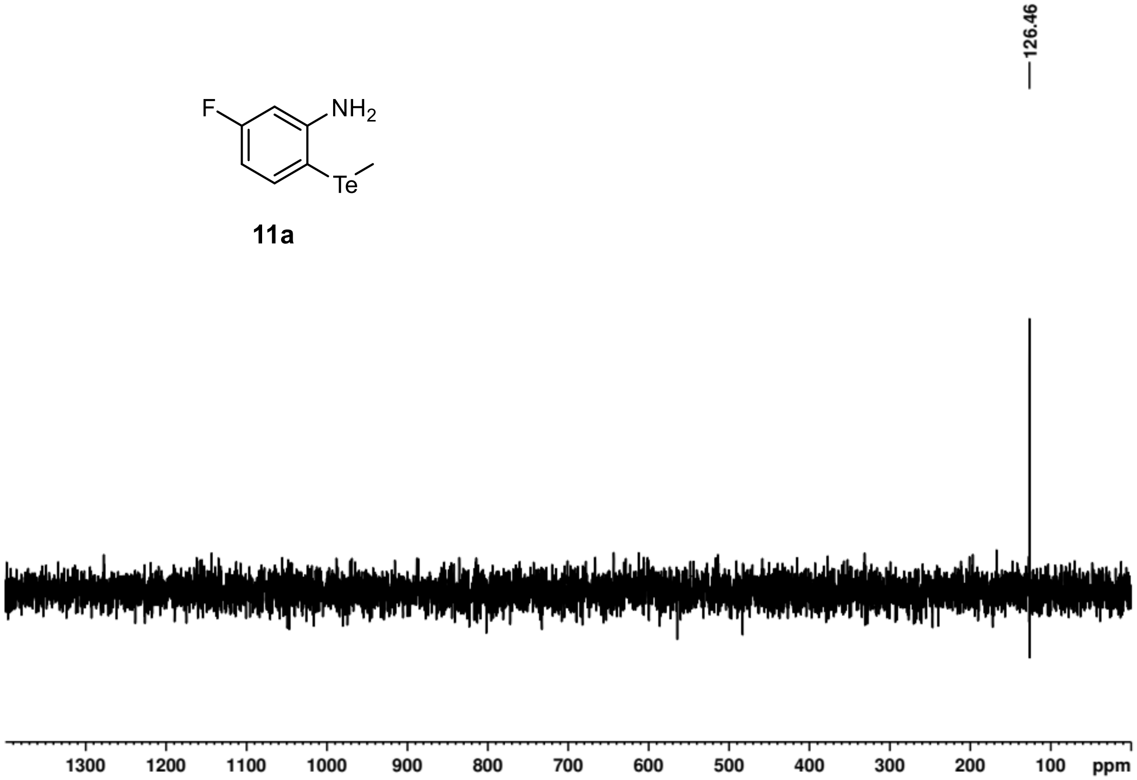
126.46
F
NH2
Te
1300 1200 1100 1000 900 800 700 600 500 400 300 200 100 ppm

11a

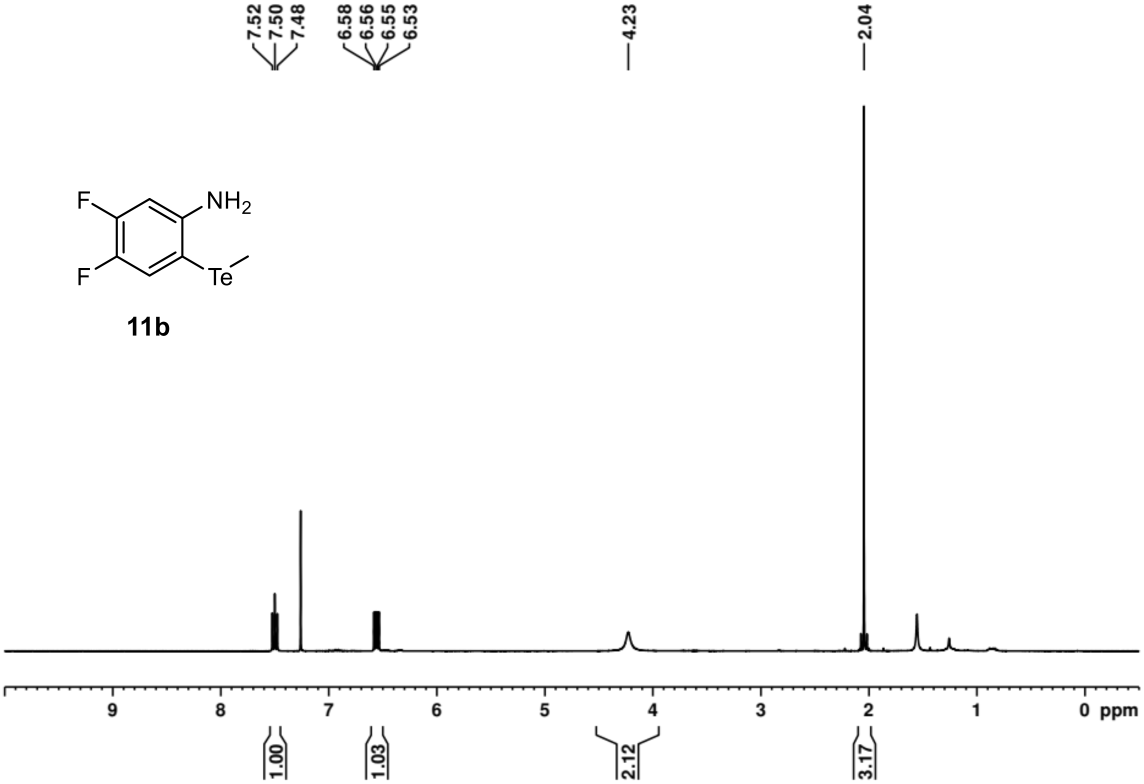
7.52
7.50
7.48
6.58
6.56
6.55
6.53
4.23
2.04
F
NH2
F
Te
9 8 7 6 5 4 3 2 1 0 ppm
1.00
1.03
2.12
3.17

11b

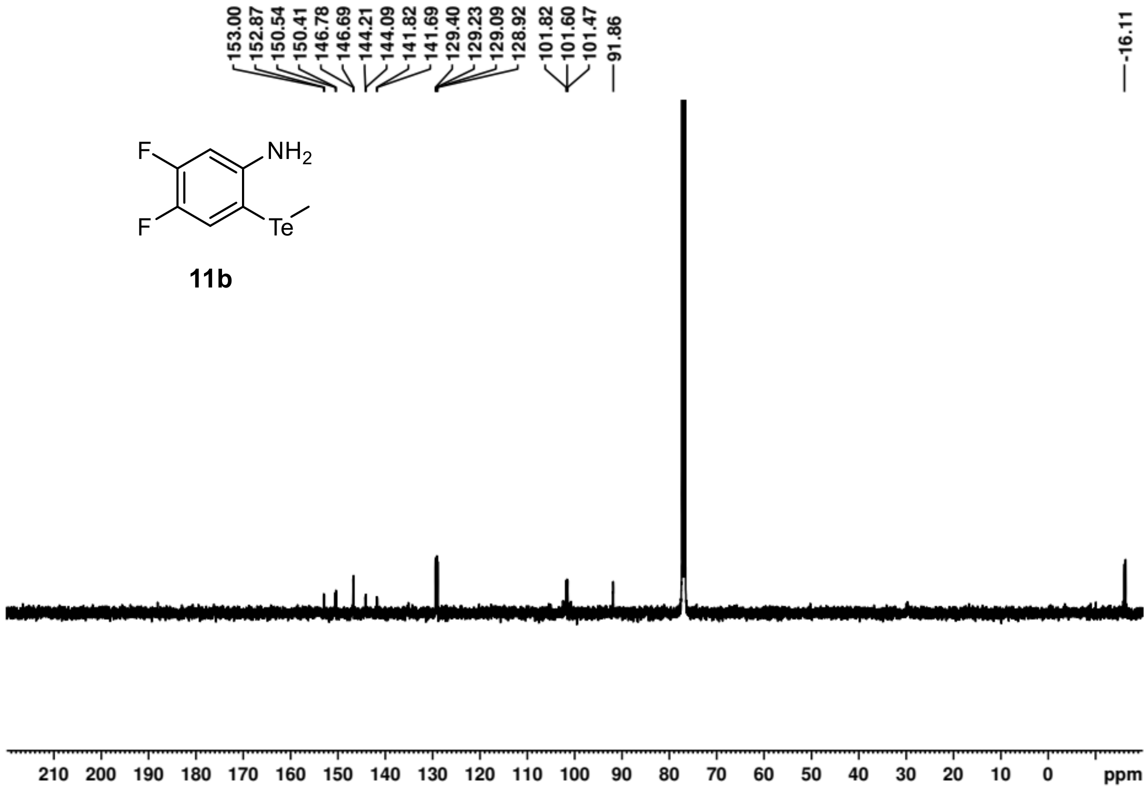
153.00
152.87
150.54
150.41
146.78
146.69
144.21
144.09
141.82
141.69
129.40
129.23
129.09
128.92
101.82
101.60
101.47
91.86
-16.11
F
F
NH2
Te
11b
210 200 190 180 170 160 150 140 130 120 110 100 90 80 70 60 50 40 30 20 10 0 ppm

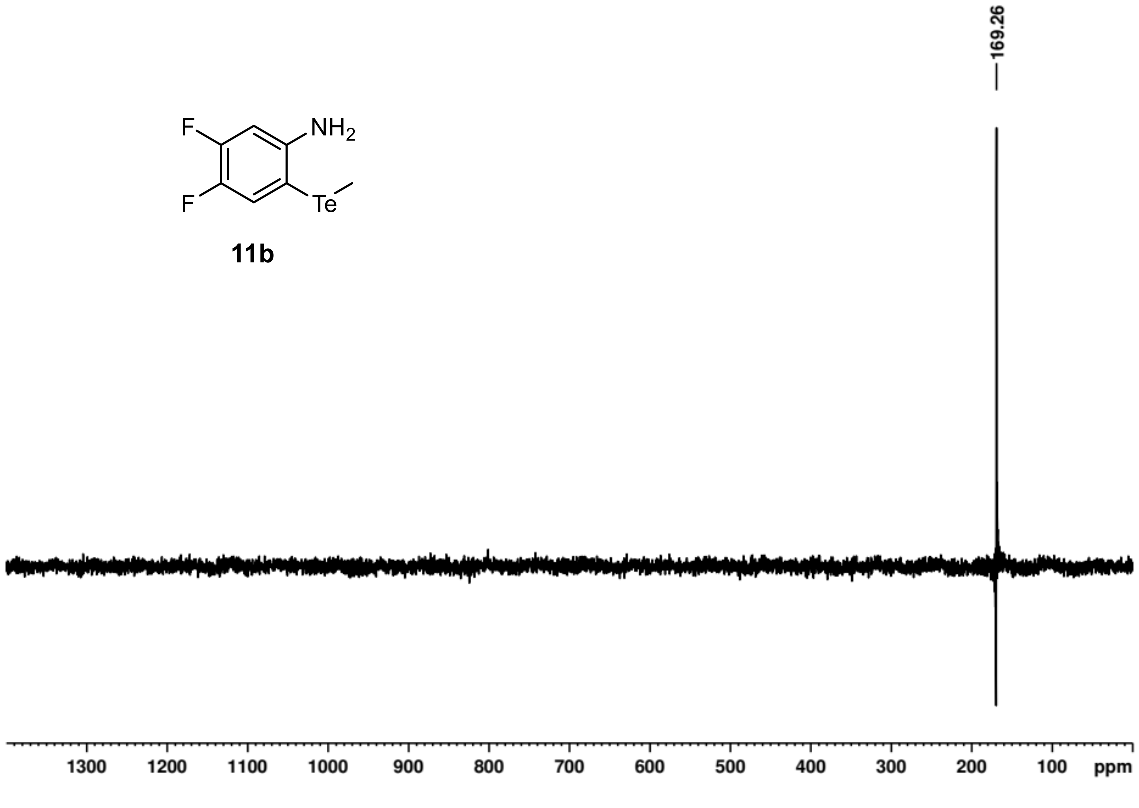
169.26
F
F
NH2
Te
11b
1300 1200 1100 1000 900 800 700 600 500 400 300 200 100 ppm

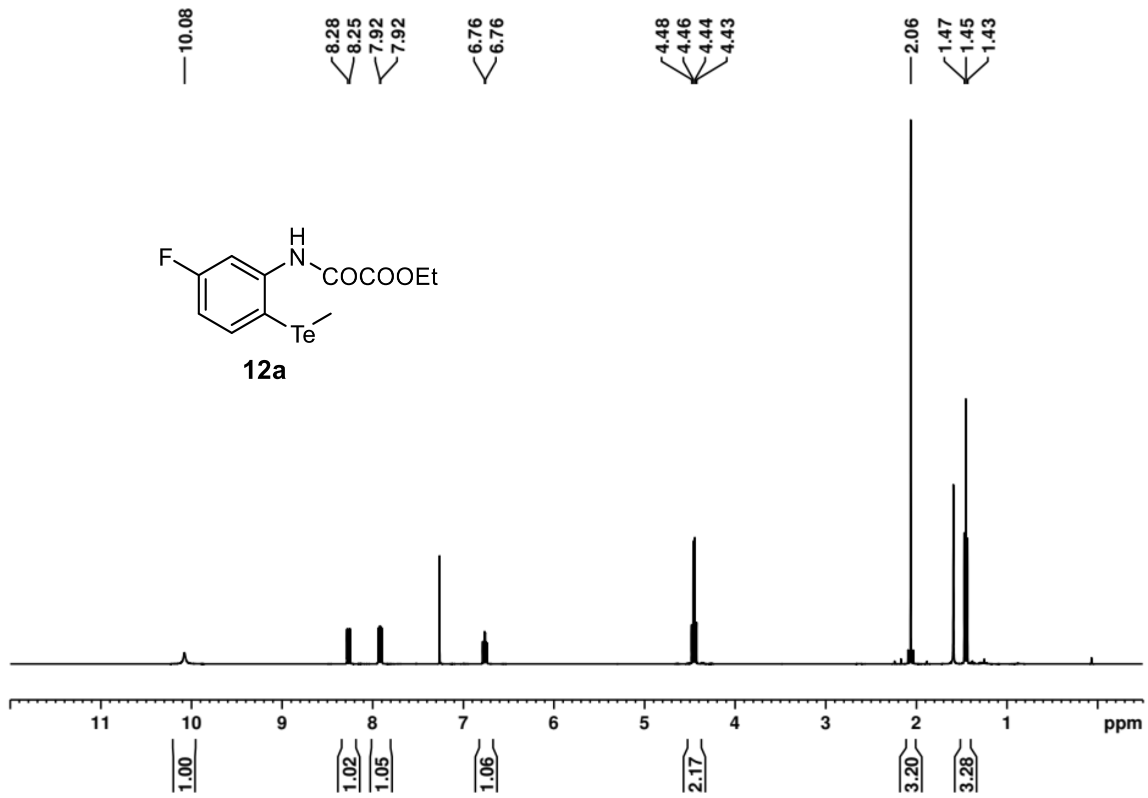
10.08
8.28
8.25
7.92
7.92
6.76
6.76
4.48
4.46
4.44
4.43
2.06
1.47
1.45
1.43
F
H
N
COCOOEt
Te
12a
11 10 9 8 7 6 5 4 3 2 1 ppm
1.00
1.02
1.05
1.06
2.17
3.20
3.28

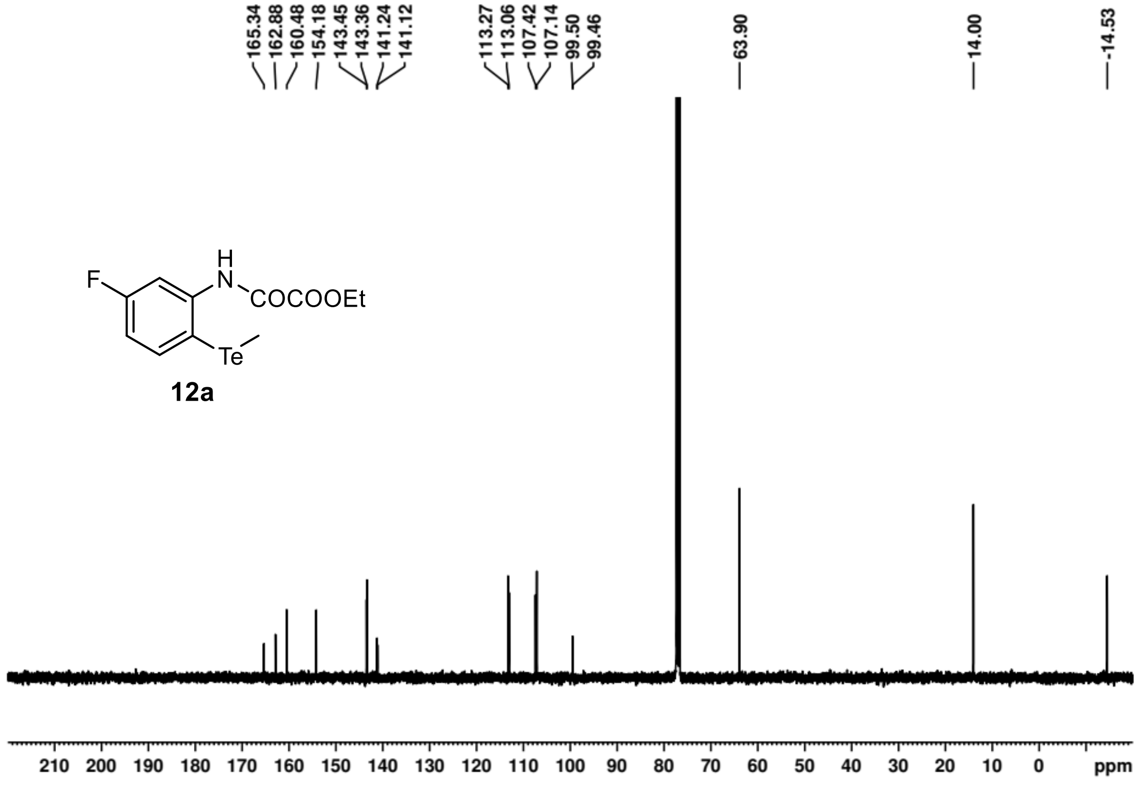
165.34
162.88
160.48
154.18
143.45
143.36
141.24
141.12
113.27
113.06
107.42
107.14
99.50
99.46
63.90
14.00
-14.53
F
H
N
COCOOEt
Te
12a
210 200 190 180 170 160 150 140 130 120 110 100 90 80 70 60 50 40 30 20 10 0 ppm

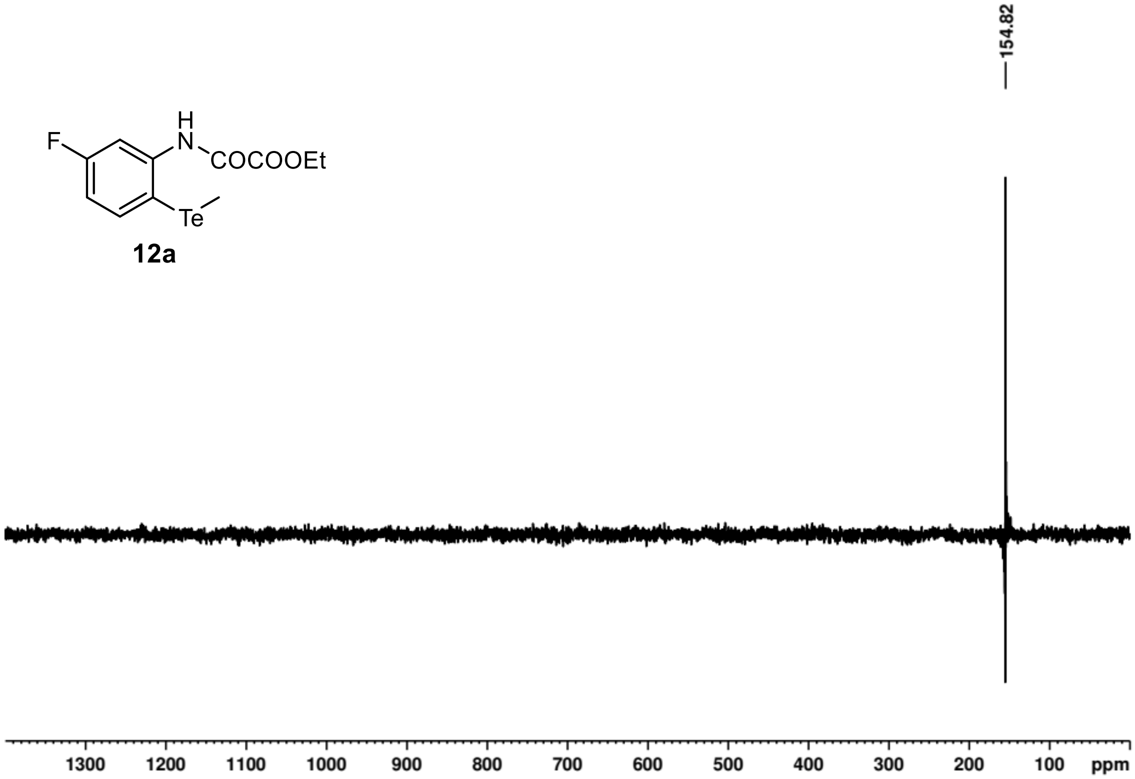

154.82
F
H
N
COCOOEt
Te
1300 1200 1100 1000 900 800 700 600 500 400 300 200 100 ppm

12a

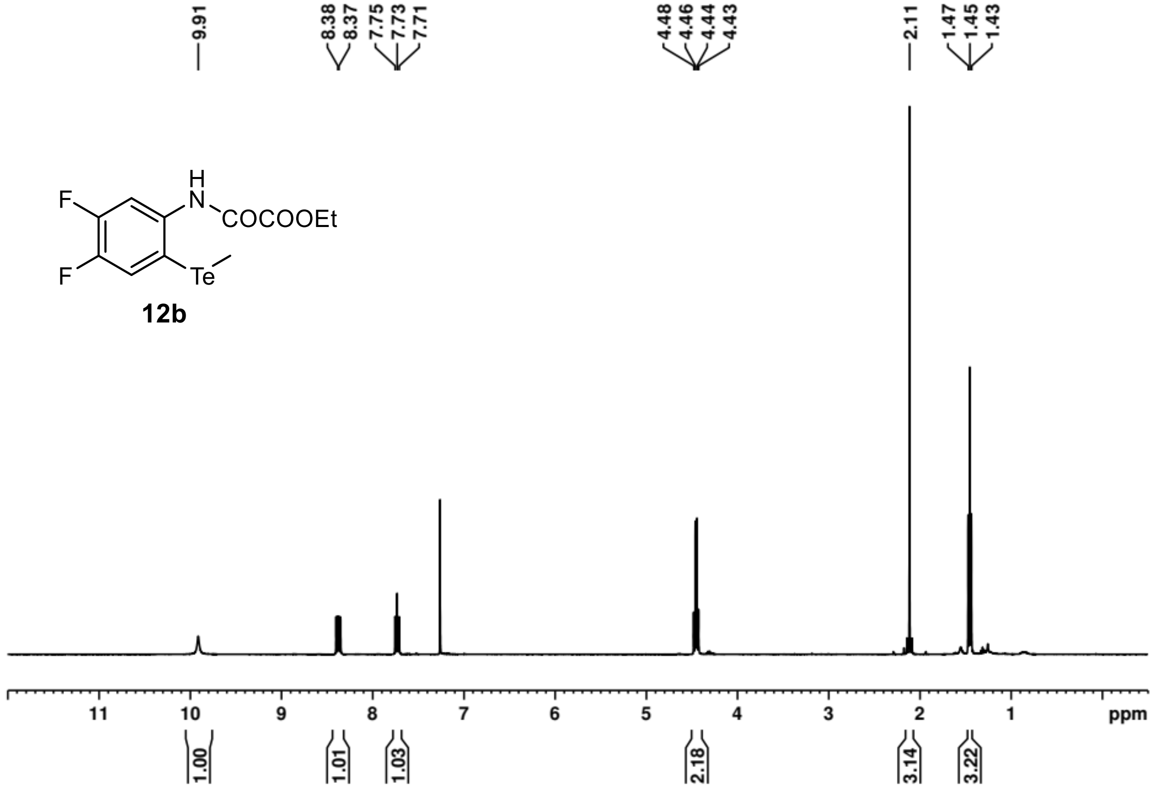

9.91
8.38
8.37
7.75
7.73
7.71
4.48
4.46
4.44
4.43
2.11
1.47
1.45
1.43
F
F
H
N
COCOOEt
Te
11 10 9 8 7 6 5 4 3 2 1 ppm
1.00
1.01
1.03
2.18
3.14
3.22

12b

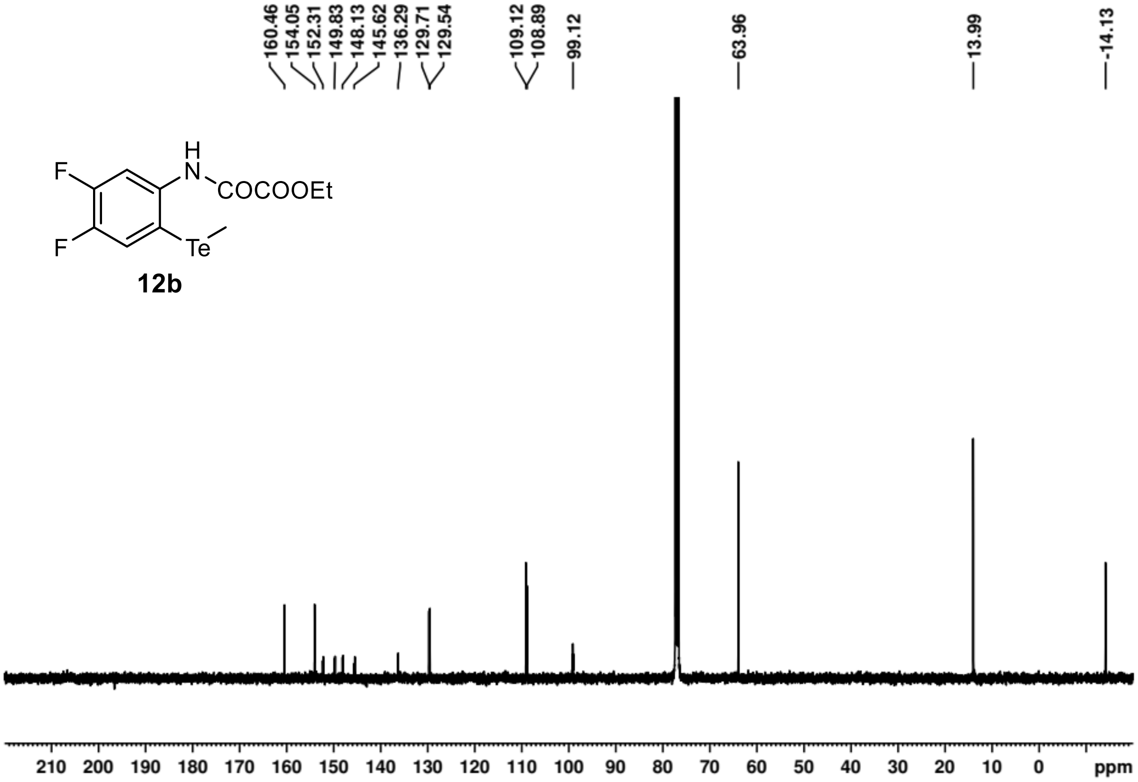
160.46
154.05
152.31
149.83
148.13
145.62
136.29
129.71
129.54
109.12
108.89
99.12
63.96
13.99
-14.13
F
F
H
N
COCOOEt
Te
12b
210 200 190 180 170 160 150 140 130 120 110 100 90 80 70 60 50 40 30 20 10 0 ppm

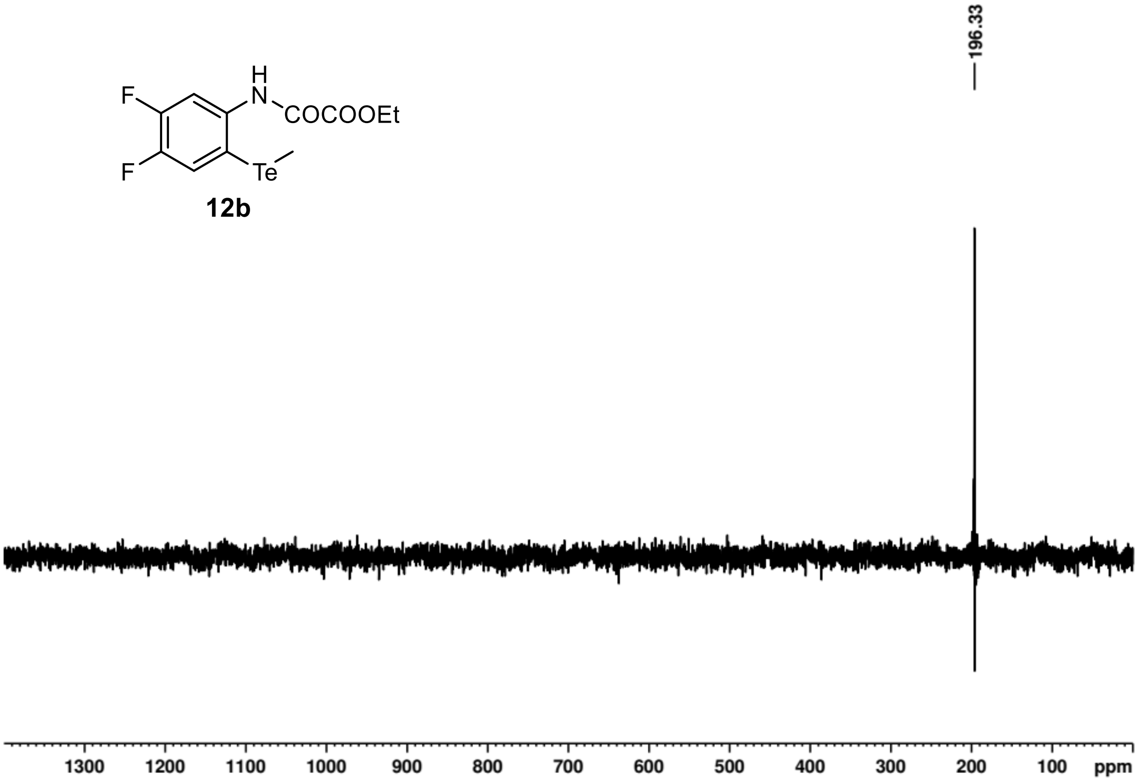
196.33
F
F
H
N
COCOOEt
Te
12b
1300 1200 1100 1000 900 800 700 600 500 400 300 200 100 ppm

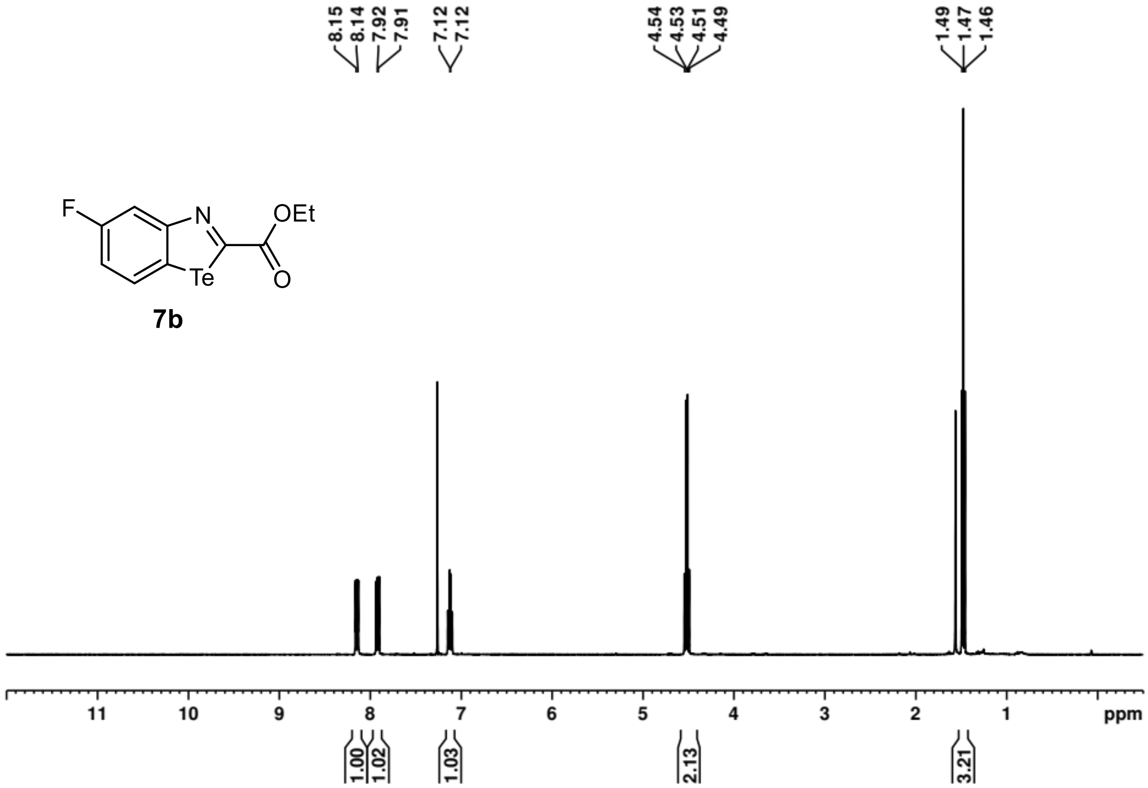
8.15
8.14
7.92
7.91
7.12
7.12
4.54
4.53
4.51
4.49
1.49
1.47
1.46
F
N
OEt
Te
O
7b
11
10
9
8
7
6
5
4
3
2
1
ppm
1.00
1.02
1.03
2.13
3.21

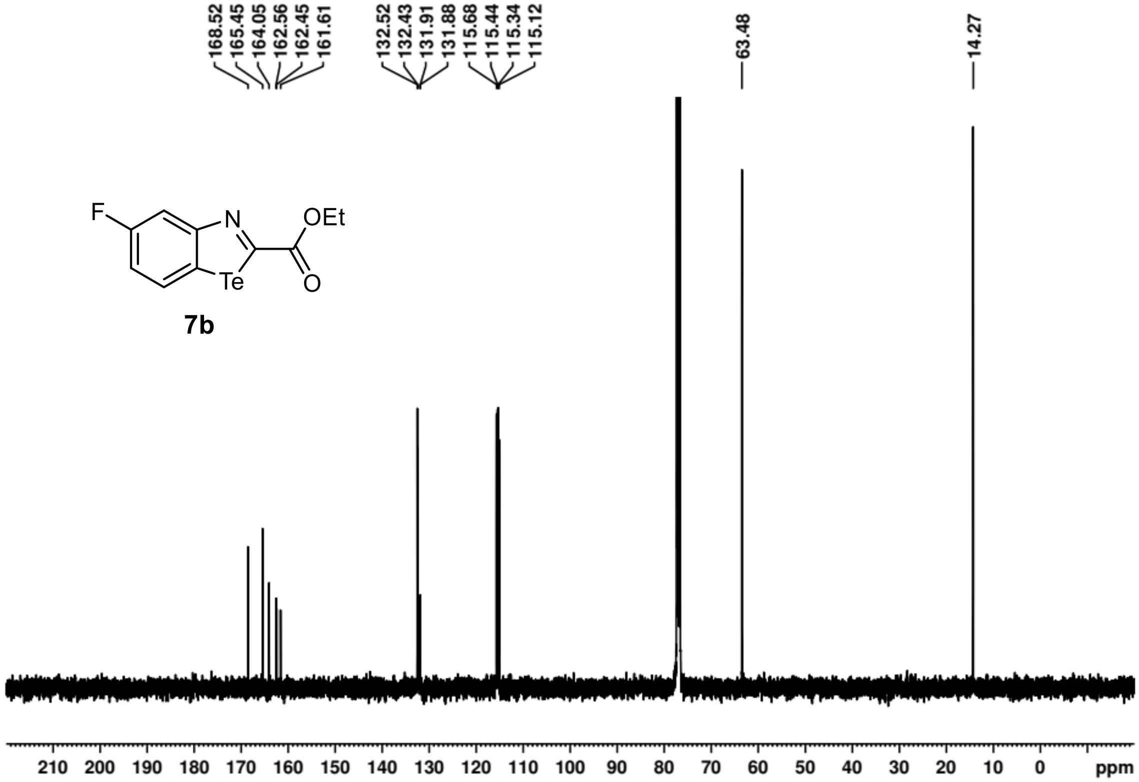
168.52
165.45
164.05
162.56
162.45
161.61
132.52
132.43
131.91
131.88
115.68
115.44
115.34
115.12
63.48
14.27
F
N
OEt
Te
O
7b
210
200
190
180
170
160
150
140
130
120
110
100
90
80
70
60
50
40
30
20
10
0
ppm

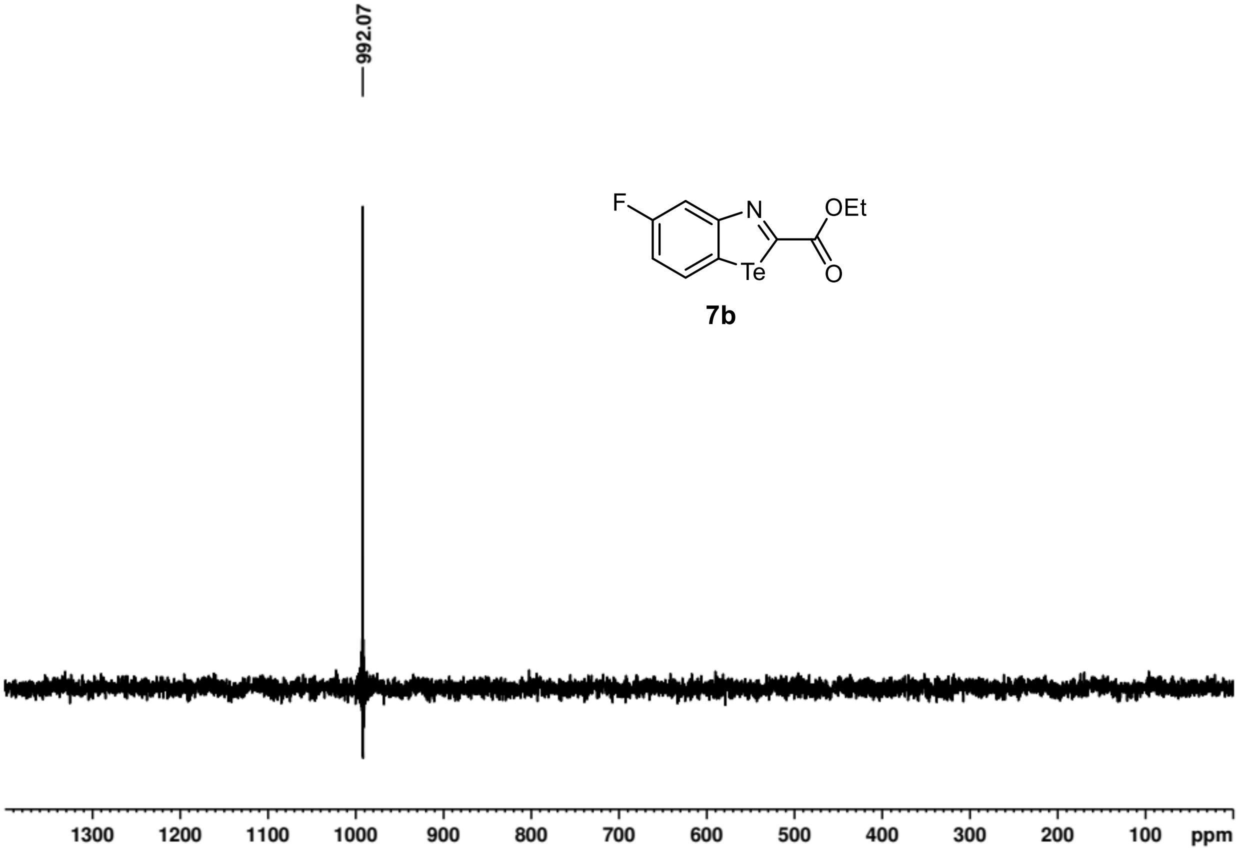

992.07
F
N
OEt
Te
O
7b
1300 1200 1100 1000 900 800 700 600 500 400 300 200 100 ppm

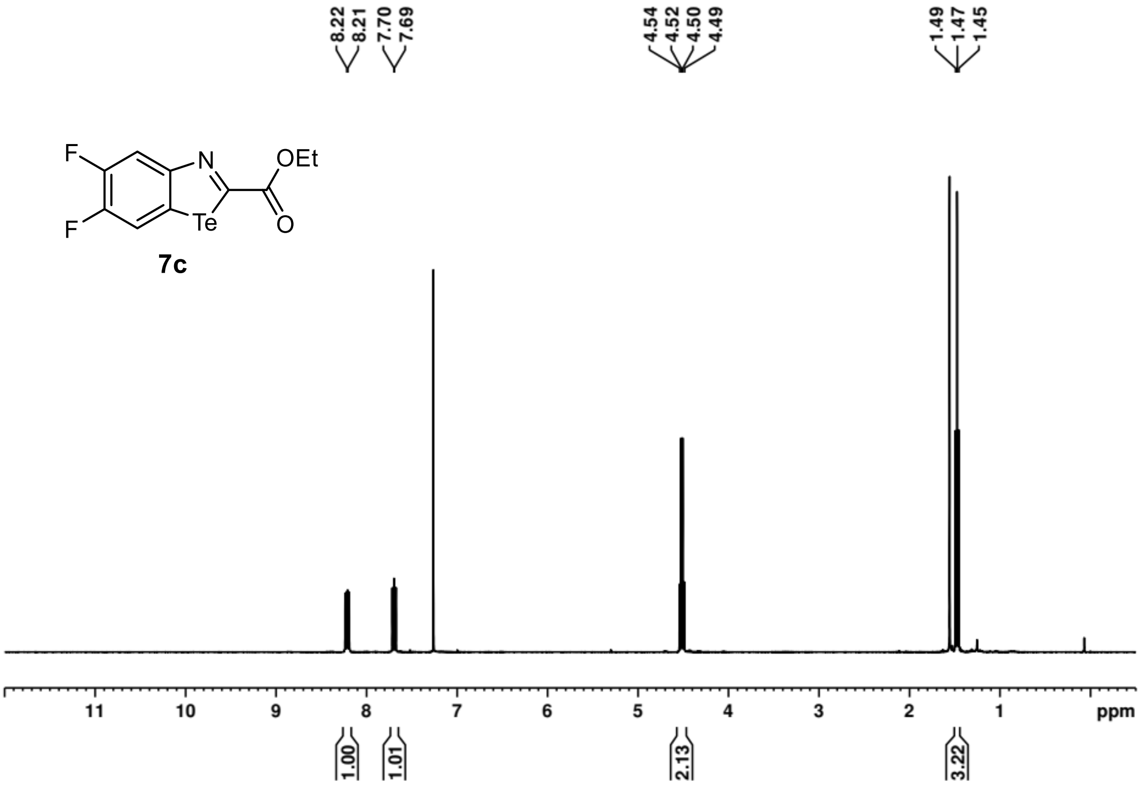

8.22
8.21
7.70
7.69
4.54
4.52
4.50
4.49
1.49
1.47
1.45
F
F
N
OEt
Te
O
7c
11 10 9 8 7 6 5 4 3 2 1 ppm
1.00
1.01
2.13
3.22

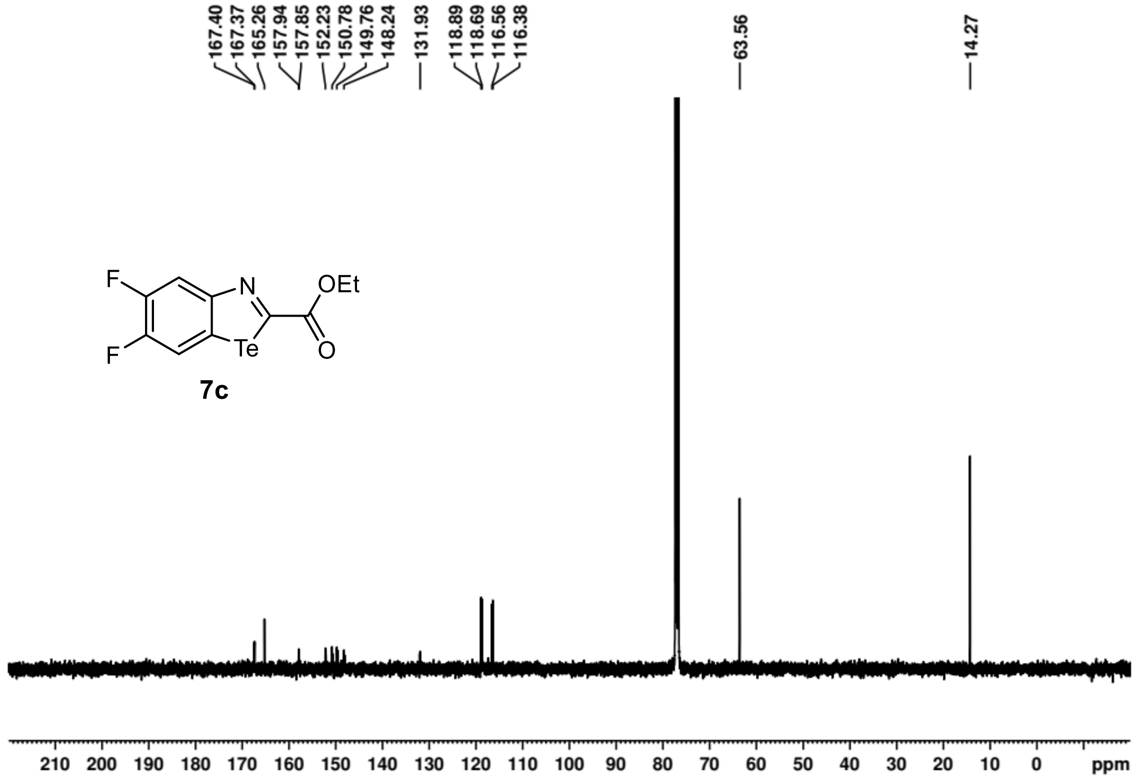
167.40
167.37
165.26
157.94
157.85
152.23
150.78
149.76
148.24
131.93
118.89
118.69
116.56
116.38
63.56
14.27
F
F
N
Te
OEt
O
7c
210 200 190 180 170 160 150 140 130 120 110 100 90 80 70 60 50 40 30 20 10 0 ppm

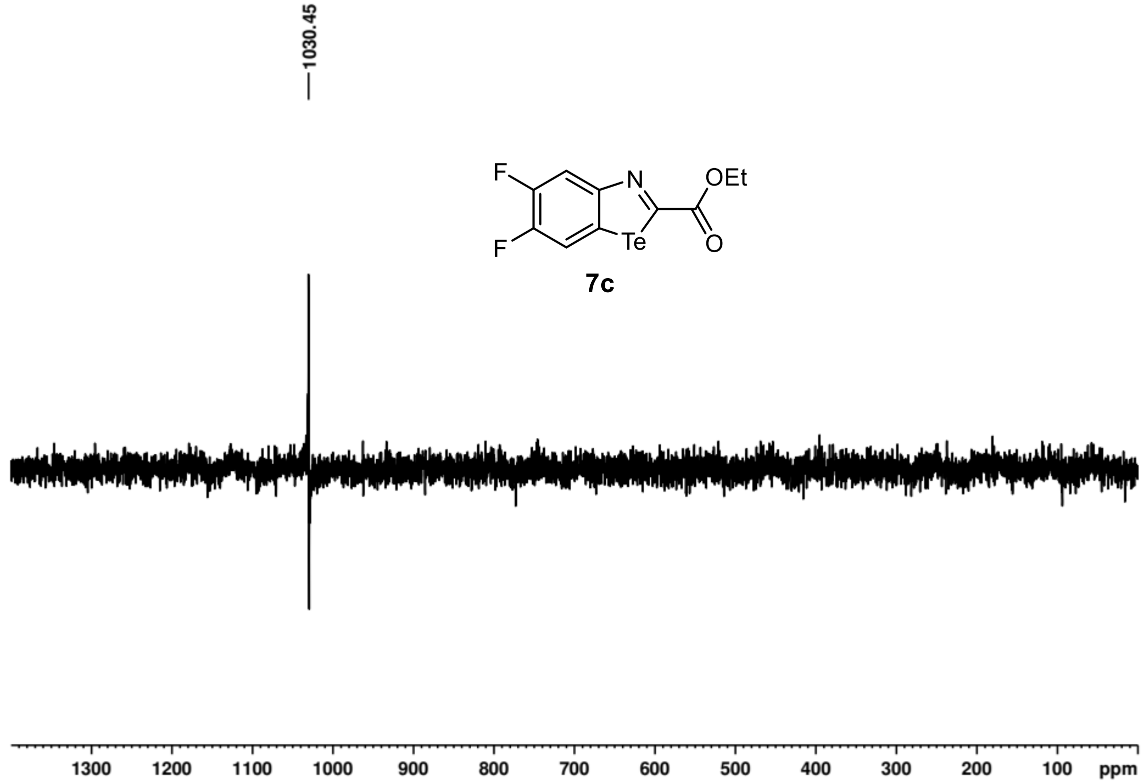
1030.45
F
F
N
Te
OEt
O
7c
1300 1200 1100 1000 900 800 700 600 500 400 300 200 100 ppm

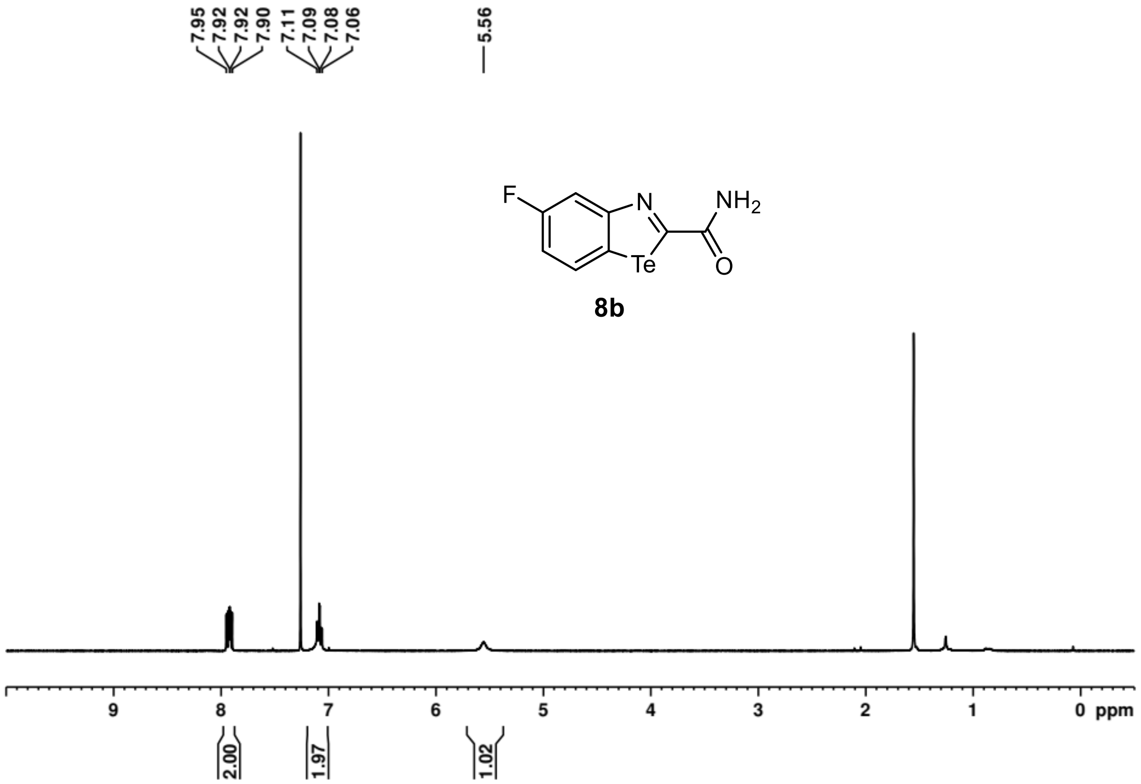
7.95
7.92
7.92
7.90
7.11
7.09
7.08
7.06
5.56
F
N
NH2
Te
O
8b
9
8
7
6
5
4
3
2
1
0 ppm
2.00
1.97
1.02

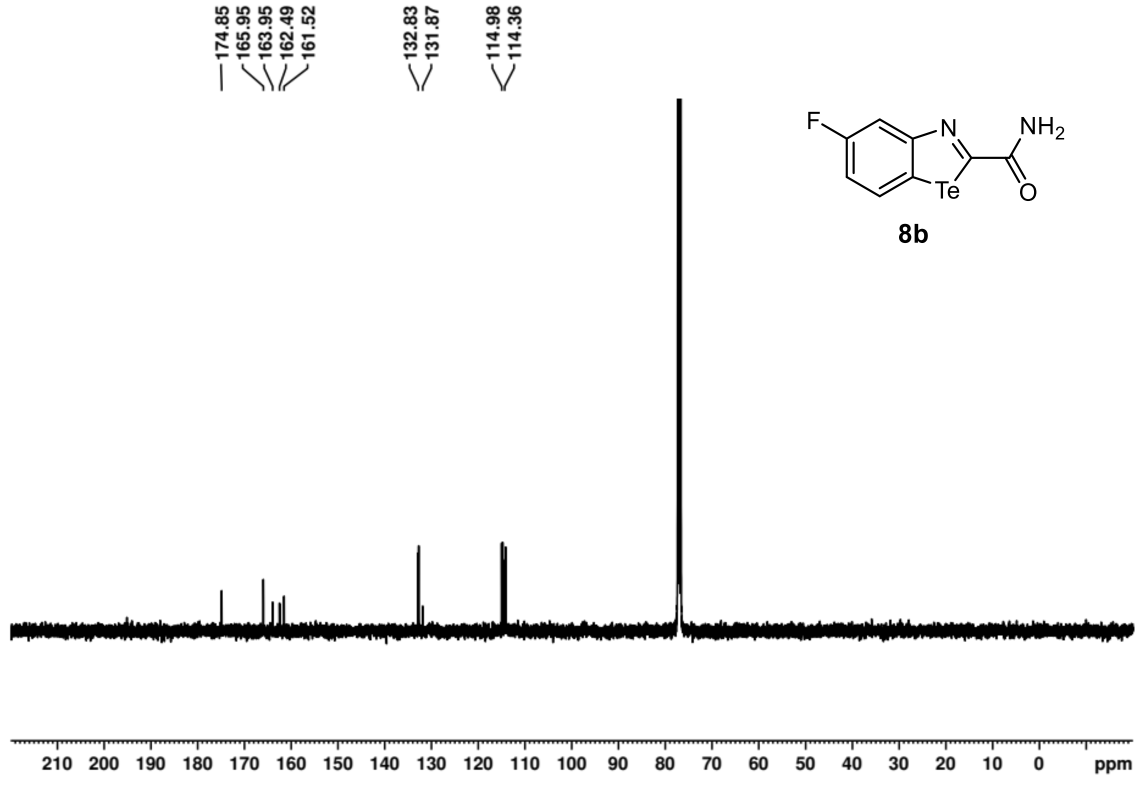
174.85
165.95
163.95
162.49
161.52
132.83
131.87
114.98
114.36
F
N
NH2
Te
O
8b
210
200
190
180
170
160
150
140
130
120
110
100
90
80
70
60
50
40
30
20
10
0
ppm

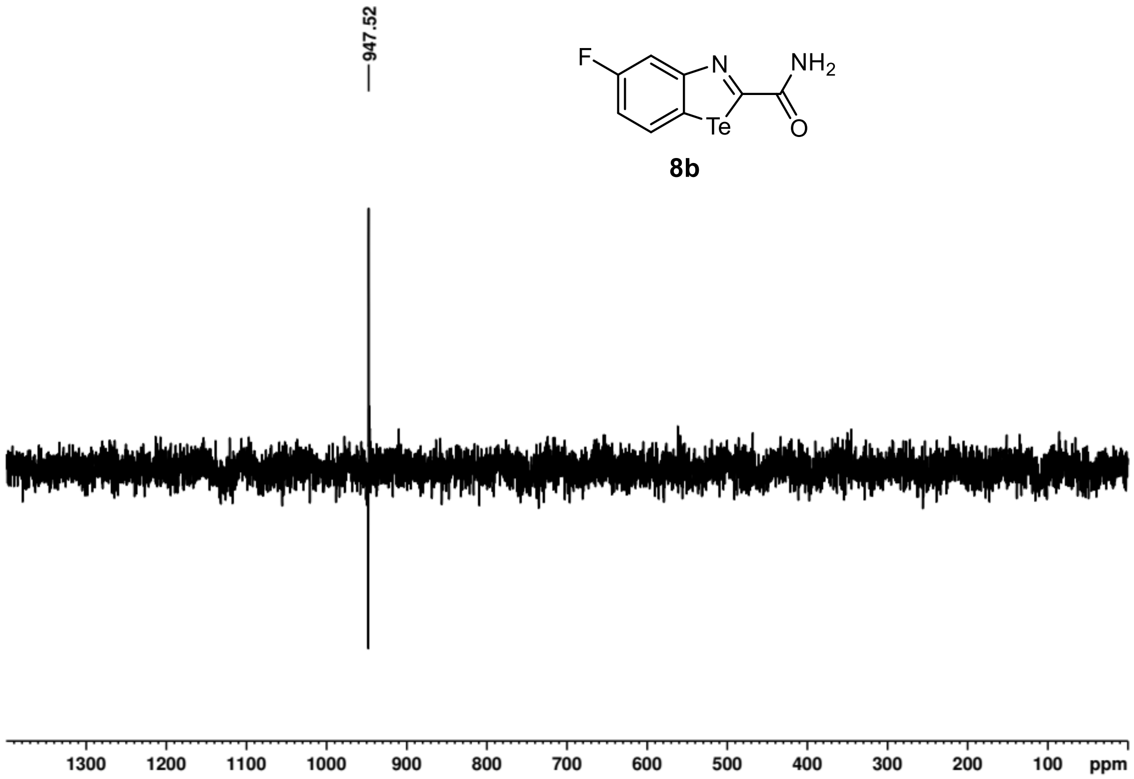
947.52
F
N
NH2
Te
O
8b
1300 1200 1100 1000 900 800 700 600 500 400 300 200 100 ppm

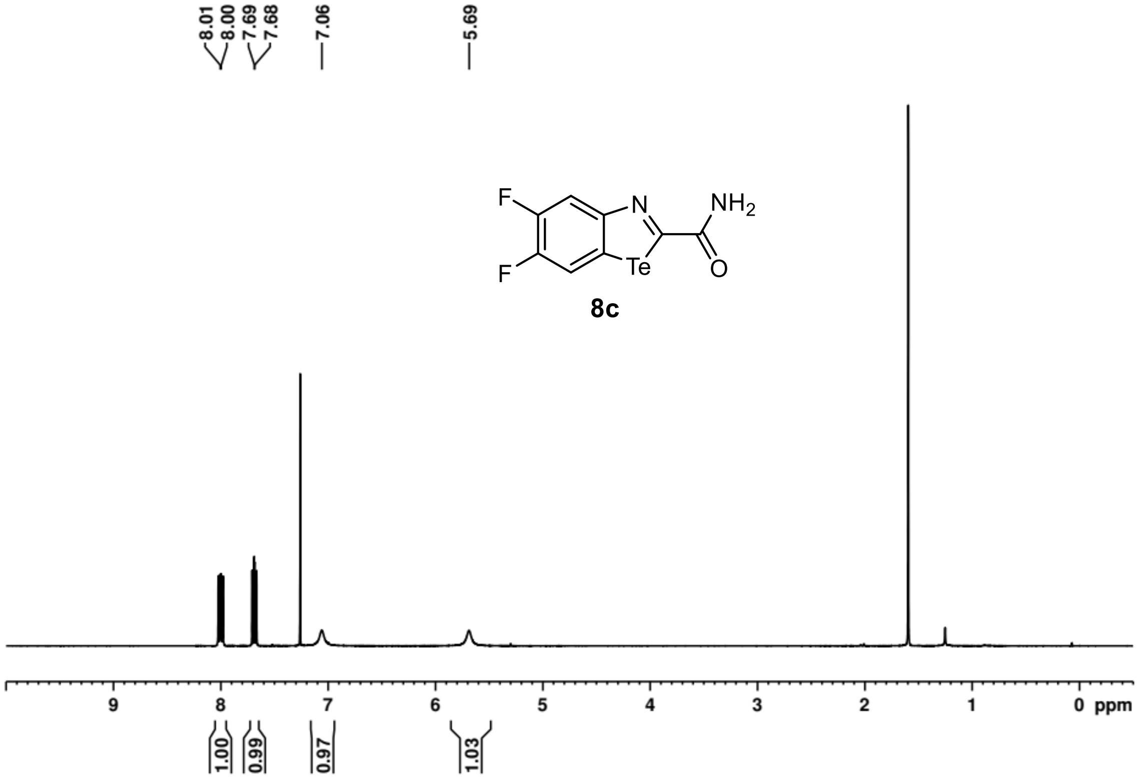
8.01
8.00
7.69
7.68
7.06
5.69
F
F
N
NH2
Te
O
8c
9 8 7 6 5 4 3 2 1 0 ppm
1.00
0.99
0.97
1.03

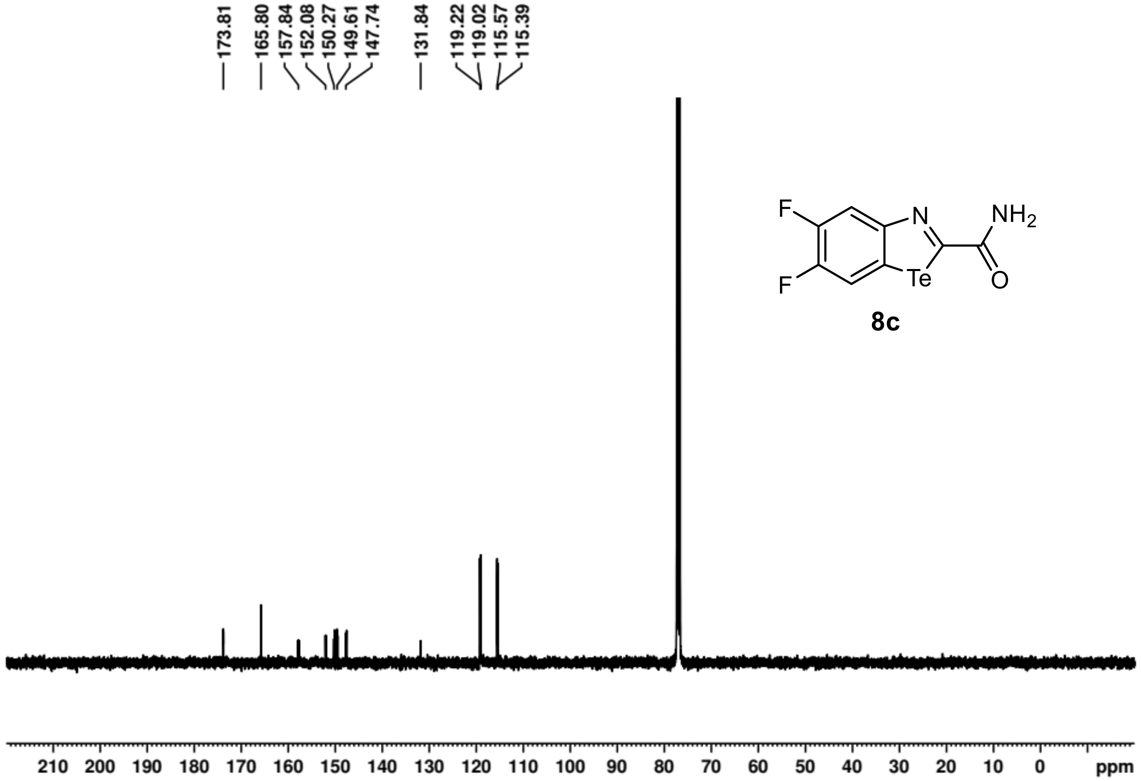
173.81
165.80
157.84
152.08
150.27
149.61
147.74
131.84
119.22
119.02
115.57
115.39
F
F
N
Te
NH2
O
8c
210 200 190 180 170 160 150 140 130 120 110 100 90 80 70 60 50 40 30 20 10 0 ppm

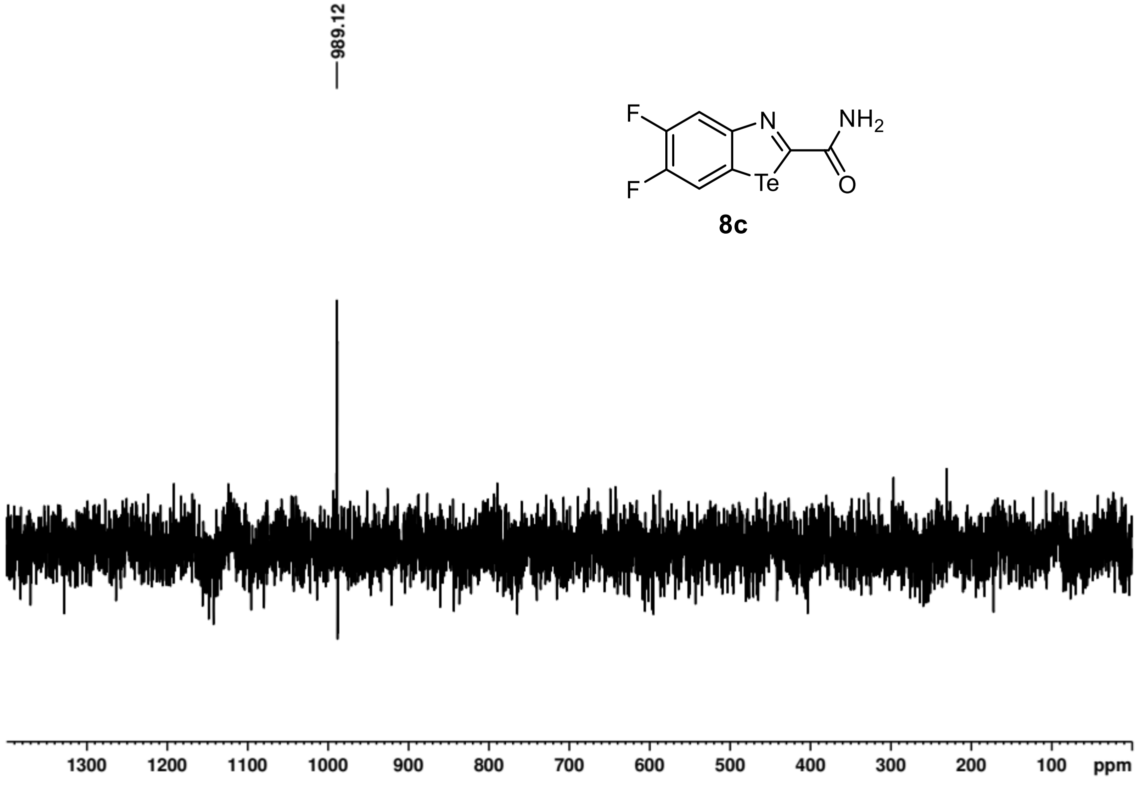
989.12
F
F
N
Te
NH2
O
8c
1300 1200 1100 1000 900 800 700 600 500 400 300 200 100 ppm

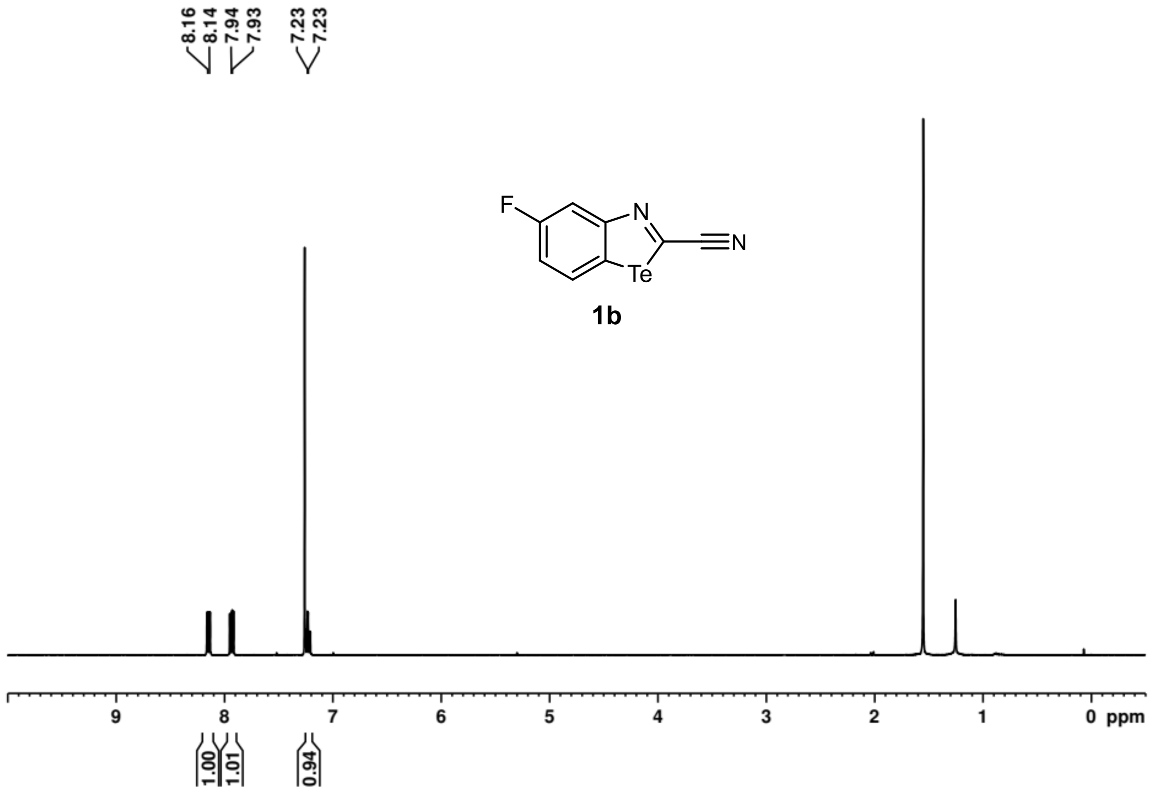
8.16
8.14
7.94
7.93
7.23
7.23
F
N
N
Te
1b
9
8
7
6
5
4
3
2
1
0 ppm
1.00
1.01
0.94

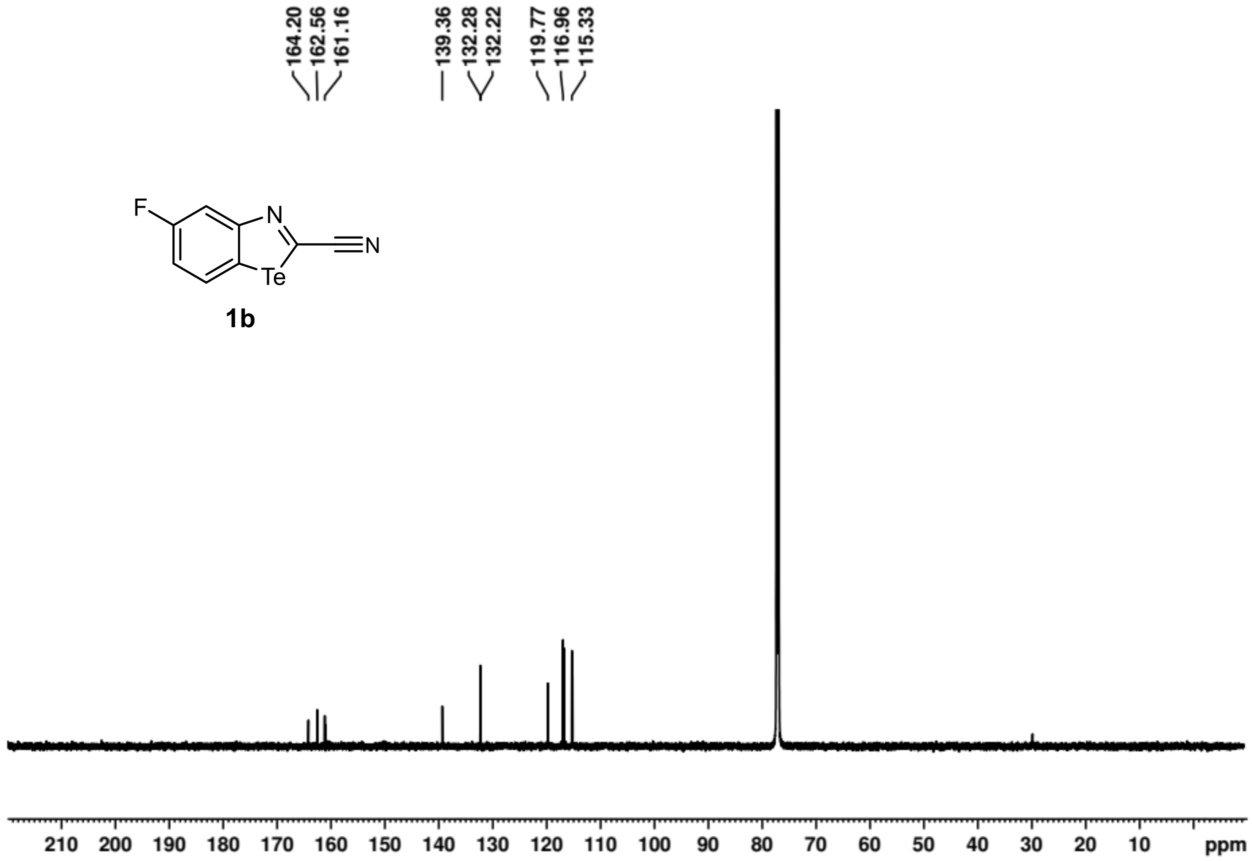
164.20
162.56
161.16
139.36
132.28
132.22
119.77
116.96
115.33
F
N
N
Te
1b
210
200
190
180
170
160
150
140
130
120
110
100
90
80
70
60
50
40
30
20
10
ppm

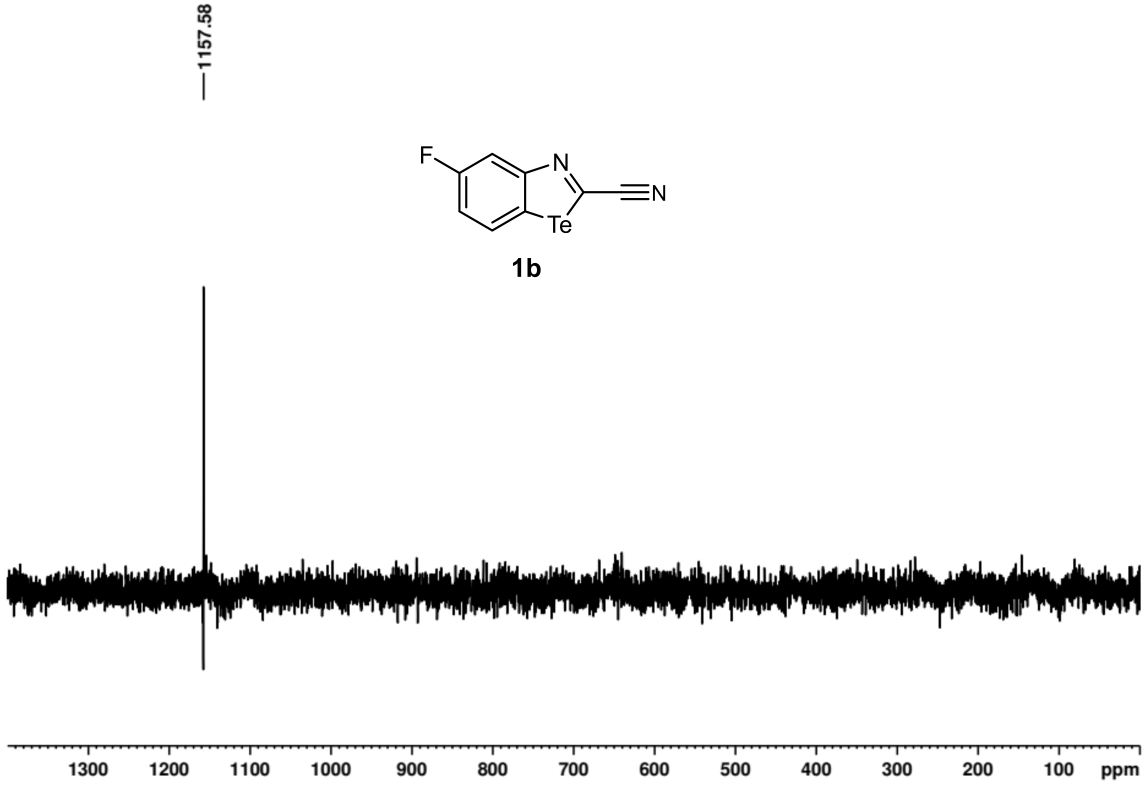
1157.58
F
N
N
Te
1b
1300
1200
1100
1000
900
800
700
600
500
400
300
200
100
ppm

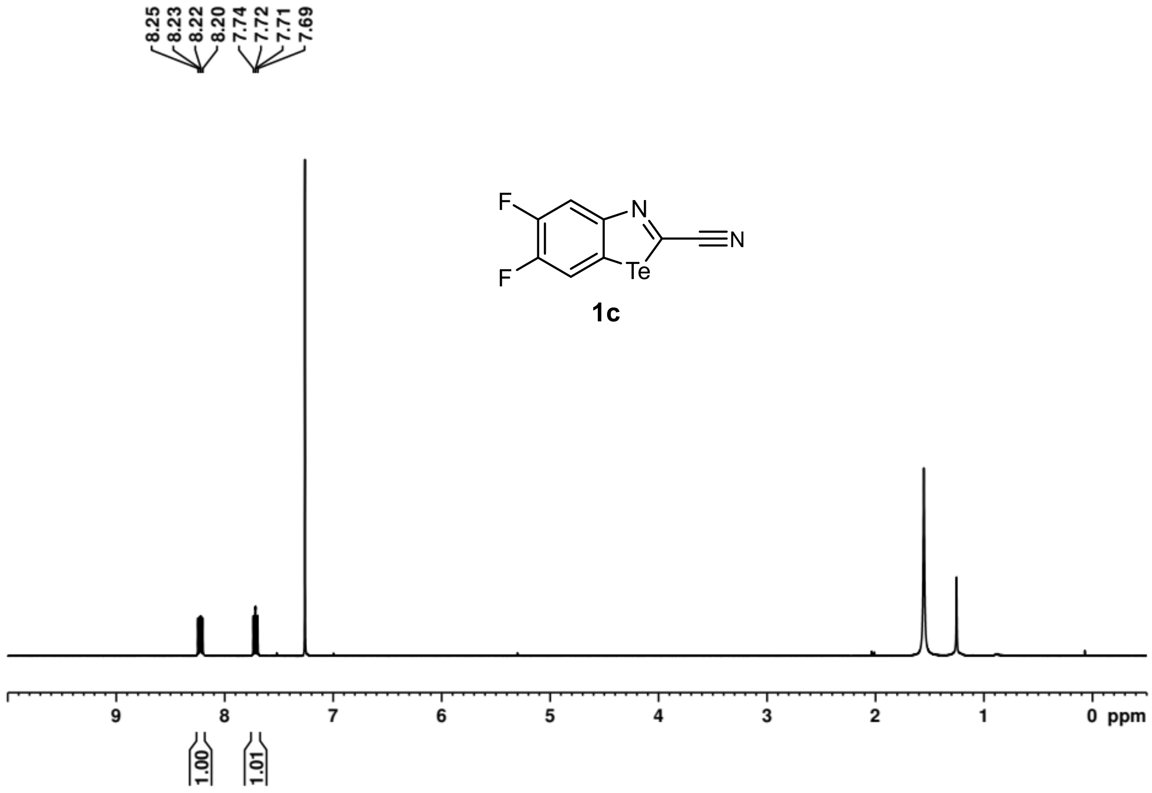
8.25
8.23
8.22
8.20
7.74
7.72
7.71
7.69
F
F
N
N
Te
1c
9
8
7
6
5
4
3
2
1
0 ppm
1.00
1.01

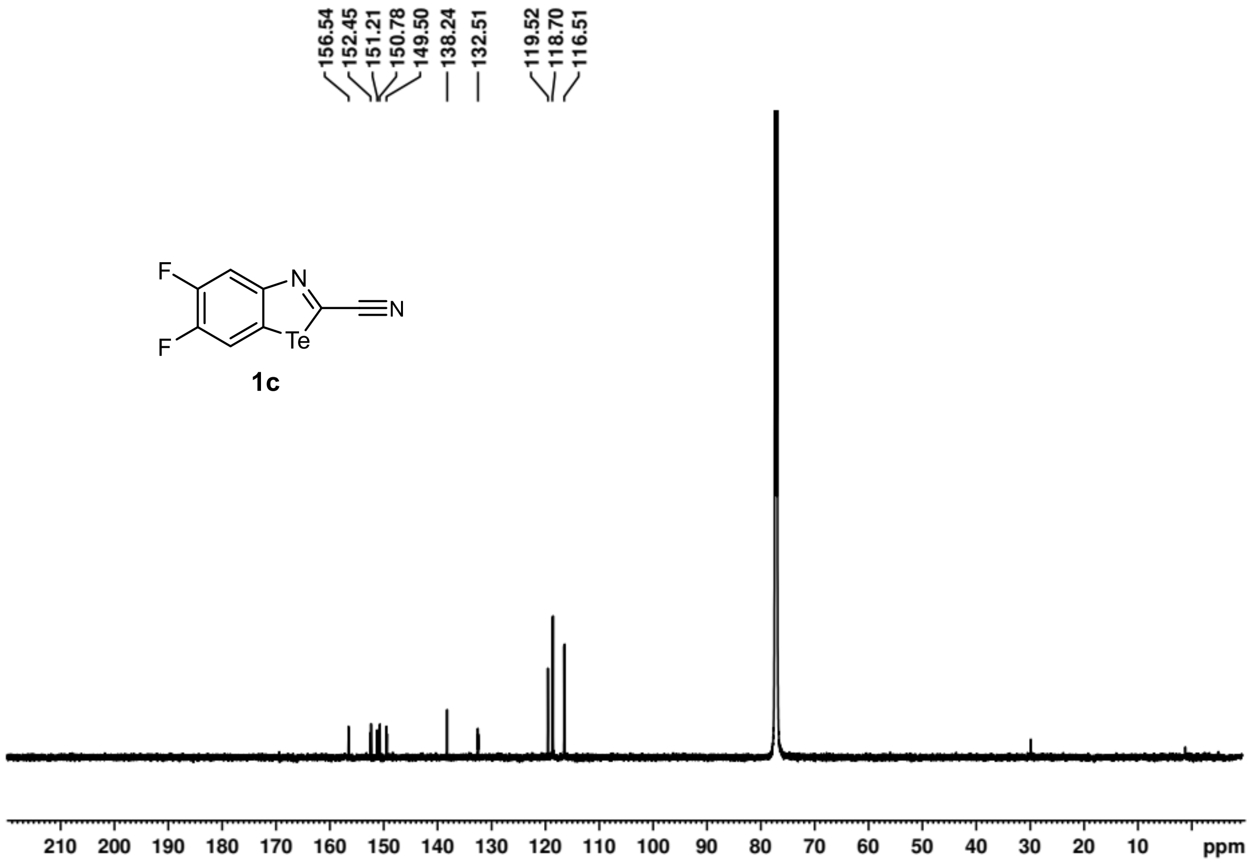
156.54
152.45
151.21
150.78
149.50
138.24
132.51
119.52
118.70
116.51
F
F
N
Te
N
1c
210 200 190 180 170 160 150 140 130 120 110 100 90 80 70 60 50 40 30 20 10 ppm

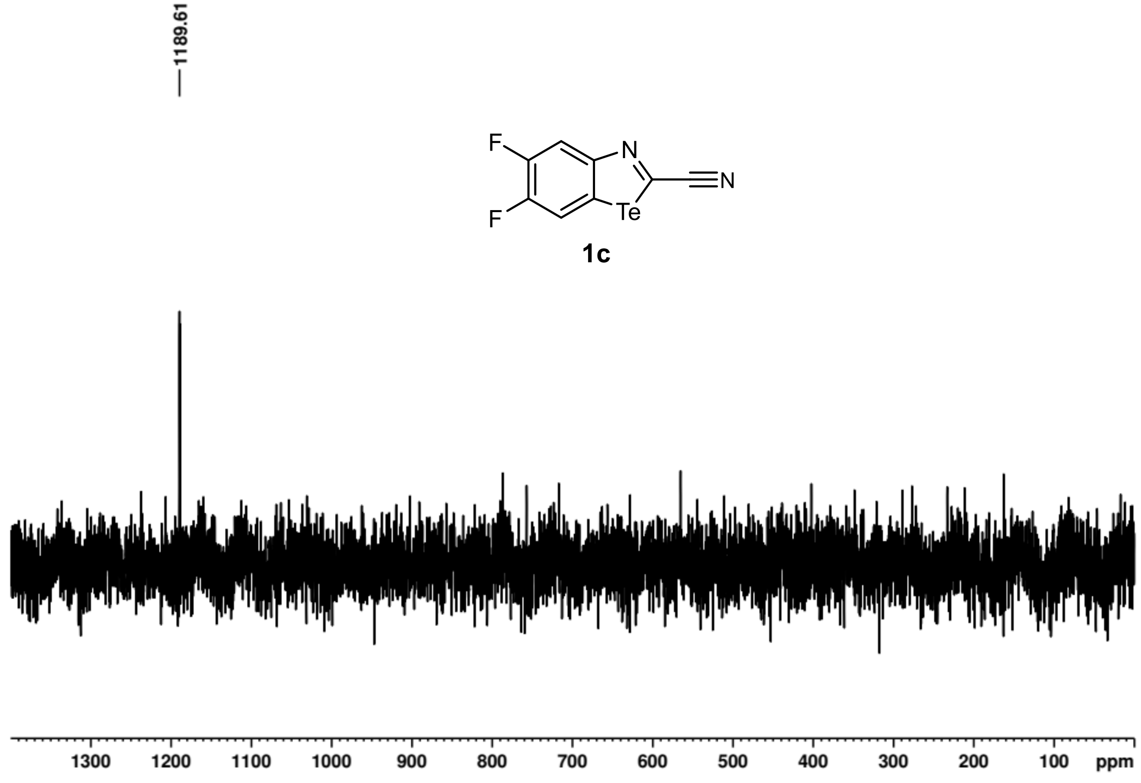
1189.61
F
F
N
Te
N
1c
1300 1200 1100 1000 900 800 700 600 500 400 300 200 100 ppm
