# Supplementary material for: Supramolecular 3D Polymers Created via Chalcogen Bonding Using Benzotellurazoles as Planar Recognition Units
Source: Chemistry. 2025 Oct 21;31(72):e02731. doi: 10.1002/chem.202502731 (PMC12731526; doi:10.1002/chem.202502731)

checkCIF/PLATON report

Structure factors have been supplied for datablock(s) jas_117m, jas_199_tw4, jas_200m, jas_207m, jas_209m, jas_211m, jas_214m, sm_151m, sm_410pym

THIS REPORT IS FOR GUIDANCE ONLY. IF USED AS PART OF A REVIEW PROCEDURE FOR PUBLICATION, IT SHOULD NOT REPLACE THE EXPERTISE OF AN EXPERIENCED CRYSTALLOGRAPHIC REFEREE.

No syntax errors found. CIF dictionary Interpreting this report

Datablock: sm_410pym

```
Bond precision:   C-C = 0.0023 A           Wavelength=0.71073

Cell:             a=29.041(3)    b=4.8698(6)         c=11.1376(13)
                  alpha=90       beta=105.953(3)     gamma=90
Temperature:      100 K

                 Calculated                    Reported
Volume           1514.5(3)                     1514.4(3)
Space group      C 2/c                         C 2/c
Hall group       -C 2yc                        -C 2yc
Moiety formula   C8 H4 N2 Te                   ?
Sum formula      C8 H4 N2 Te                   C8 H4 N2 Te
Mr               255.73                        255.73
Dx,g cm-3        2.243                         2.243
Z                8                             8
Mu (mm-1)        3.853                         3.853
F000             944.0                         944.0
F000'            940.01
h,k,lmax         48,8,18                       48,8,18
Nref             3686                          3676
Tmin,Tmax        0.356,0.463                   0.510,0.750
Tmin'            0.241

Correction method= # Reported T Limits: Tmin=0.510 Tmax=0.750
AbsCorr = MULTI-SCAN
```

```
Data completeness= 0.997          Theta(max)= 36.352

R(reflections)= 0.0196( 3618)                 wR2(reflections)= 0.0490( 3676)

S = 1.357               Npar= 100
```

```
The following ALERTS were generated. Each ALERT has the format
       test-name_ALERT_alert-type_alert-level.
Click on the hyperlinks for more details of the test.
```

Alert level C

```
PLAT767_ALERT_4_C INS Embedded LIST 6 Instruction Should be LIST 4     Please Check
PLAT906_ALERT_3_C Large K Value in the Analysis of Variance ......      2.252 Check
PLAT911_ALERT_3_C Missing FCF Refl Between Thmin & STh/L=    0.600          8 Report
                1  1  0,   5  1  0,  -6  0  2,   2  0  2,   3  1  2,   6  0  2,
               -4  0  4,  -2  0  4,
PLAT971_ALERT_2_C Check Calcd Resid. Dens.  0.67Ang From Te1             1.63 eA-3
PLAT971_ALERT_2_C Check Calcd Resid. Dens.  0.66Ang From Te1             1.59 eA-3
```

Alert level G

```
PLAT128_ALERT_4_G Alternate Setting for Input Space Group     C2/c       I2/a Note
PLAT432_ALERT_2_G Short Inter X...Y Contact  Te1      ..N2       .       3.13 Ang.
                                              1-x,-y,1-z  =      5_656 Check
PLAT432_ALERT_2_G Short Inter X...Y Contact  Te1      ..N1       .       3.14 Ang.
                                            x,1-y,-1/2+z  =      6_565 Check
PLAT910_ALERT_3_G Missing FCF Reflection(s) Below Theta(Min)[Deg]=       2.92 Note
                2  0  0,
PLAT912_ALERT_4_G Missing # of FCF Reflections Above STh/L=  0.600          1 Note
PLAT913_ALERT_3_G Missing # of Very Strong Reflections in FCF ....          3 Note
                5  1  0,   3  1  2,  -4  0  4,
PLAT969_ALERT_5_G The 'Henn et al.' R-Factor-gap value ..........      3.959 Note
              Predicted wR2: Based on SigI**2  1.24 or SHELX Weight  3.61
PLAT978_ALERT_2_G Number C-C Bonds with Positive Residual Density.          7 Info
```

```
   0 ALERT level A = Most likely a serious problem - resolve or explain
   0 ALERT level B = A potentially serious problem, consider carefully
   5 ALERT level C = Check. Ensure it is not caused by an omission or oversight
   8 ALERT level G = General information/check it is not something unexpected

   0 ALERT type 1 CIF construction/syntax error, inconsistent or missing data
   5 ALERT type 2 Indicator that the structure model may be wrong or deficient
   4 ALERT type 3 Indicator that the structure quality may be low
   3 ALERT type 4 Improvement, methodology, query or suggestion
   1 ALERT type 5 Informative message, check
```

Datablock: jas_214m

```
Bond precision:   C-C = 0.0011 A          Wavelength=0.71073
```

Cell:	a=28.453(2)	b=4.8332(4)	c=11.0718(9)
	alpha=90	beta=92.221(3)	gamma=90
Temperature:	100 K		

	Calculated	Reported
Volume	1521.4(2)	1521.5(2)
Space group	C 2/c	C 2/c
Hall group	-C 2yc	-C 2yc
Moiety formula	C8 H3 F N2 Te	?
Sum formula	C8 H3 F N2 Te	C8 H3 F N2 Te
Mr	273.72	273.72
Dx,g cm-3	2.390	2.390
Z	8	8
Mu (mm-1)	3.862	3.862
F000	1008.0	1008.0
F000'	1004.14	
h,k,lmax	52,8,20	51,8,20
Nref	4892	4871
Tmin,Tmax	0.621,0.802	0.560,0.750
Tmin'	0.333	

Correction method= # Reported T Limits: Tmin=0.560 Tmax=0.750
AbsCorr = MULTI-SCAN

Data completeness= 0.996 Theta(max)= 40.593

R(reflections)= 0.0121(4599) wR2(reflections)= 0.0310(4871)

S = 1.076 Npar= 109

The following ALERTS were generated. Each ALERT has the format
test-name_ALERT_alert-type_alert-level.
Click on the hyperlinks for more details of the test.

Alert level C

```
PLAT230_ALERT_2_C Hirshfeld Test Diff for    Te1      --C1       .        6.0 s.u.
```

Alert level G

```
PLAT230_ALERT_2_G Hirshfeld Test Diff for    C1       --C8       .        5.7 s.u.
PLAT432_ALERT_2_G Short Inter X...Y Contact  Te1      ..N2       .       3.12 Ang.
                                          -x,2-y,1-z  =      5_576 Check
PLAT432_ALERT_2_G Short Inter X...Y Contact  Te1      ..N1       .       3.15 Ang.
                                        x,1-y,-1/2+z  =      6_565 Check
PLAT910_ALERT_3_G Missing FCF Reflection(s) Below Theta(Min)[Deg]=       2.87 Note
                2  0  0,
```

```
PLAT912_ALERT_4_G Missing # of FCF Reflections Above STh/L=  0.600         18 Note
PLAT933_ALERT_2_G Number of HKL-OMIT Records in Embedded .res File          1 Note
                2  0  0,
PLAT969_ALERT_5_G The 'Henn et al.' R-Factor-gap value ..........      2.743 Note
              Predicted wR2: Based on SigI**2  1.13 or SHELX Weight  2.88
PLAT978_ALERT_2_G Number C-C Bonds with Positive Residual Density.          7 Info
```

---

0 **ALERT level A** = Most likely a serious problem - resolve or explain
0 **ALERT level B** = A potentially serious problem, consider carefully
1 **ALERT level C** = Check. Ensure it is not caused by an omission or oversight
8 **ALERT level G** = General information/check it is not something unexpected

0 ALERT type 1 CIF construction/syntax error, inconsistent or missing data
6 ALERT type 2 Indicator that the structure model may be wrong or deficient
1 ALERT type 3 Indicator that the structure quality may be low
1 ALERT type 4 Improvement, methodology, query or suggestion
1 ALERT type 5 Informative message, check

Datablock: jas_209m

Bond precision: C-C = 0.0017 A   Wavelength=0.71073

Cell:	a=30.6995(16)	b=4.7346(3)	c=11.1438(6)
	alpha=90	beta=105.443(2)	gamma=90
Temperature:	100 K		

	Calculated	Reported
Volume	1561.27(15)	1561.27(15)
Space group	C 2/c	C 2/c
Hall group	-C 2yc	-C 2yc
Moiety formula	C8 H2 F2 N2 Te	?
Sum formula	C8 H2 F2 N2 Te	C8 H2 F2 N2 Te
Mr	291.72	291.72
Dx,g cm-3	2.482	2.482
Z	8	8
Mu (mm-1)	3.789	3.789
F000	1072.0	1072.0
F000'	1068.27	
h,k,lmax	56,8,20	56,8,20
Nref	4986	4976
Tmin,Tmax	0.689,0.794	0.560,0.750
Tmin'	0.396	

Correction method= # Reported T Limits: Tmin=0.560 Tmax=0.750
AbsCorr = MULTI-SCAN

Data completeness= 0.998 Theta(max)= 40.476

R(reflections)= 0.0196(4296) wR2(reflections)= 0.0431(4976)

S = 1.035 Npar= 118

The following ALERTS were generated. Each ALERT has the format
test-name_ALERT_alert-type_alert-level.
Click on the hyperlinks for more details of the test.

Alert level G

```
PLAT128_ALERT_4_G Alternate Setting for Input Space Group     C2/c       I2/a Note
PLAT432_ALERT_2_G Short Inter X...Y Contact  Te1      ..N2       .       3.10 Ang.
                                               -x,-y,-z  =      5_555 Check
PLAT432_ALERT_2_G Short Inter X...Y Contact  Te1      ..N1       .       3.21 Ang.
                                            x,1-y,1/2+z  =      6_566 Check
PLAT434_ALERT_2_G Short Inter HL..HL Contact F1       ..F1       .       2.82 Ang.
                                         1/2-x,5/2-y,-z  =      7_575 Check
PLAT910_ALERT_3_G Missing FCF Reflection(s) Below Theta(Min)[Deg]=       2.75 Note
                2  0  0,
PLAT912_ALERT_4_G Missing # of FCF Reflections Above STh/L=  0.600          9 Note
PLAT933_ALERT_2_G Number of HKL-OMIT Records in Embedded .res File          1 Note
                2  0  0,
PLAT969_ALERT_5_G The 'Henn et al.' R-Factor-gap value ..........      1.867 Note
              Predicted wR2: Based on SigI**2  2.30 or SHELX Weight  4.16
PLAT978_ALERT_2_G Number C-C Bonds with Positive Residual Density.          7 Info
```

---

0 **ALERT level A** = Most likely a serious problem - resolve or explain
0 **ALERT level B** = A potentially serious problem, consider carefully
0 **ALERT level C** = Check. Ensure it is not caused by an omission or oversight
9 **ALERT level G** = General information/check it is not something unexpected

0 ALERT type 1 CIF construction/syntax error, inconsistent or missing data
5 ALERT type 2 Indicator that the structure model may be wrong or deficient
1 ALERT type 3 Indicator that the structure quality may be low
2 ALERT type 4 Improvement, methodology, query or suggestion
1 ALERT type 5 Informative message, check

Datablock: sm_151m

Bond precision:	C-C = 0.0071 A		Wavelength=0.71073
Cell:	a=5.8498(6)	b=11.2897(9)	c=23.372(3)
	alpha=90	beta=90	gamma=90
Temperature:	100 K		

	Calculated	Reported
Volume	1543.6(3)	1543.6(3)
Space group	P b c a	P b c a
Hall group	-P 2ac 2ab	-P 2ac 2ab
Moiety formula	C8 H7 N Te	?
Sum formula	C8 H7 N Te	C8 H7 N Te
Mr	244.75	244.75
Dx,g cm-3	2.106	2.106
Z	8	8
Mu (mm-1)	3.771	3.771
F000	912.0	912.0
F000’	908.01	
h,k,lmax	8,16,33	8,16,33
Nref	2357	2356
Tmin,Tmax	0.672,0.854	0.500,0.750
Tmin’	0.659	

Correction method= # Reported T Limits: Tmin=0.500 Tmax=0.750
AbsCorr = MULTI-SCAN

Data completeness= 1.000 Theta(max)= 30.501

R(reflections)= 0.0470(1647) wR2(reflections)= 0.0968(2356)

S = 1.062 Npar= 92

The following ALERTS were generated. Each ALERT has the format
test-name_ALERT_alert-type_alert-level.
Click on the hyperlinks for more details of the test.

Alert level C

```
PLAT767_ALERT_4_C INS Embedded LIST 6 Instruction Should be LIST 4     Please Check
PLAT906_ALERT_3_C Large K Value in the Analysis of Variance ......      7.742 Check
PLAT972_ALERT_2_C Check Calcd Resid. Dens.  0.99Ang From Te1            -1.91 eA-3
```

Alert level G

```
PLAT432_ALERT_2_G Short Inter X...Y Contact  Te1      ..N1       .       3.15 Ang.
                                             1/2-x,-1/2+y,z  =      8_655 Check
PLAT910_ALERT_3_G Missing FCF Reflection(s) Below Theta(Min)[Deg]=       3.49 Note
                0  0  2,
PLAT912_ALERT_4_G Missing # of FCF Reflections Above STh/L=  0.600          1 Note
PLAT933_ALERT_2_G Number of HKL-OMIT Records in Embedded .res File          1 Note
                0  0  2,
PLAT965_ALERT_2_G The SHELXL WEIGHT Optimisation has not Converged     Please Check
PLAT969_ALERT_5_G The 'Henn et al.' R-Factor-gap value ..........       1.940 Note
              Predicted wR2: Based on SigI**2  4.99 or SHELX Weight  9.12
PLAT978_ALERT_2_G Number C-C Bonds with Positive Residual Density.          2 Info
```

0 **ALERT level A** = Most likely a serious problem - resolve or explain
0 **ALERT level B** = A potentially serious problem, consider carefully
3 **ALERT level C** = Check. Ensure it is not caused by an omission or oversight
7 **ALERT level G** = General information/check it is not something unexpected

0 ALERT type 1 CIF construction/syntax error, inconsistent or missing data
5 ALERT type 2 Indicator that the structure model may be wrong or deficient
2 ALERT type 3 Indicator that the structure quality may be low
2 ALERT type 4 Improvement, methodology, query or suggestion
1 ALERT type 5 Informative message, check

Datablock: jas_117m

Bond precision: C-C = 0.0059 A Wavelength=1.54178

Cell:	a=22.3251(15)	b=5.3627(4)	c=11.2099(7)
	alpha=90	beta=93.9891(15)	gamma=90
Temperature:	100 K		

	Calculated	Reported
Volume	1338.83(16)	1338.83(16)
Space group	P 21/c	P 21/c
Hall group	-P 2ybc	-P 2ybc
Moiety formula	C14 H19 N Te	?
Sum formula	C14 H19 N Te	C14 H19 N Te
Mr	328.90	328.90
Dx,g cm-3	1.632	1.632
Z	4	4
Mu (mm-1)	17.312	17.312
F000	648.0	648.0
F000’	648.88	
h,k,lmax	28,6,14	28,6,14
Nref	2900	2895
Tmin,Tmax	0.022,0.518	0.010,0.140
Tmin’	0.003	

Correction method= # Reported T Limits: Tmin=0.010 Tmax=0.140
AbsCorr = NUMERICAL

Data completeness= 0.998 Theta(max)= 79.243

R(reflections)= 0.0313(2839) wR2(reflections)= 0.0852(2895)

S = 1.112 Npar= 146

The following ALERTS were generated. Each ALERT has the format
test-name_ALERT_alert-type_alert-level.
Click on the hyperlinks for more details of the test.

Alert level C

```
PLAT094_ALERT_2_C Ratio of Maximum / Minimum Residual Density ....       2.96 Report
PLAT934_ALERT_3_C Number of (Iobs-Icalc)/Sigma(W) > 10 Outliers ..          1 Check
                0   6   3,
PLAT971_ALERT_2_C Check Calcd Resid. Dens.  0.94Ang From Te1             2.22 eA-3
PLAT971_ALERT_2_C Check Calcd Resid. Dens.  0.94Ang From Te1             2.15 eA-3
```

Alert level G

```
PLAT432_ALERT_2_G Short Inter X...Y Contact  Te1      ..N1       .       3.11 Ang.
                                              x,3/2-y,1/2+z  =      4_576 Check
PLAT910_ALERT_3_G Missing FCF Reflection(s) Below Theta(Min)[Deg]=       3.97 Note
                1   0   0,
PLAT912_ALERT_4_G Missing # of FCF Reflections Above STh/L=  0.600          4 Note
PLAT969_ALERT_5_G The 'Henn et al.' R-Factor-gap value ..........       4.280 Note
              Predicted wR2: Based on SigI**2  1.99 or SHELX Weight  7.66
PLAT978_ALERT_2_G Number C-C Bonds with Positive Residual Density.          4 Info
```

0 **ALERT level A** = Most likely a serious problem - resolve or explain
0 **ALERT level B** = A potentially serious problem, consider carefully
4 **ALERT level C** = Check. Ensure it is not caused by an omission or oversight
5 **ALERT level G** = General information/check it is not something unexpected

0 ALERT type 1 CIF construction/syntax error, inconsistent or missing data
5 ALERT type 2 Indicator that the structure model may be wrong or deficient
2 ALERT type 3 Indicator that the structure quality may be low
1 ALERT type 4 Improvement, methodology, query or suggestion
1 ALERT type 5 Informative message, check

Datablock: jas_199_tw4

Bond precision: C-C = 0.0088 A Wavelength=1.54178

Cell:	a=9.6880(6)	b=7.3158(5)	c=15.3467(10)
	alpha=90	beta=108.287(3)	gamma=90
Temperature:	100 K		

	Calculated	Reported
Volume	1032.77(12)	1032.77(12)
Space group	P 21/n	P 21/n
Hall group	-P 2yn	-P 2yn
Moiety formula	C10 H8 F N O2 Te	?
Sum formula	C10 H8 F N O2 Te	C10 H8 F N O2 Te
Mr	320.77	320.77
Dx,g cm-3	2.063	2.063
Z	4	4
Mu (mm-1)	22.728	22.728
F000	608.0	608.0
F000'	609.33	
h,k,lmax	12,9,19	12,9,19
Nref	2257	2244
Tmin,Tmax	0.144,0.336	0.030,0.180
Tmin'	0.008	

Correction method= # Reported T Limits: Tmin=0.030 Tmax=0.180
AbsCorr = MULTI-SCAN

Data completeness= 0.994 Theta(max)= 79.599

R(reflections)= 0.0513(2078) wR2(reflections)= 0.1331(2244)

S = 1.097 Npar= 137

The following ALERTS were generated. Each ALERT has the format
test-name_ALERT_alert-type_alert-level.
Click on the hyperlinks for more details of the test.

Alert level A

PLAT971_ALERT_2_A Check Calcd Resid. Dens. 0.94Ang From Te1 3.59 eA-3

Author Response: Residual absorption, only the major two twin domains could be accounted for.

Alert level B

PLAT094_ALERT_2_B Ratio of Maximum / Minimum Residual Density .... 5.55 Report

Author Response: Residual absorption, only the major two twin domains could be accounted for.

```
Alert level C
DIFMX02_ALERT_1_C  The maximum difference density is > 0.1*ZMAX*0.75
            The relevant atom site should be identified.
PLAT097_ALERT_2_C Large Reported Max.  (Positive) Residual Density       4.19 eA-3
PLAT342_ALERT_3_C Low Bond Precision on  C-C Bonds ..............    0.00875 Ang.
PLAT971_ALERT_2_C Check Calcd Resid. Dens.  0.85Ang From Te1             1.96 eA-3
```

Author Response: Residual absorption, only the major two twin domains could be accounted for.

```
PLAT975_ALERT_2_C Check Calcd Resid. Dens.  0.93Ang From O1      .       1.20 eA-3
PLAT975_ALERT_2_C Check Calcd Resid. Dens.  0.98Ang From O1      .       0.43 eA-3
PLAT992_ALERT_5_C Repd & Actual _reflns_number_gt Values Differ by         17 Check
```

```
Alert level G
PLAT432_ALERT_2_G Short Inter X...Y Contact  Te1      ..O1       .       3.03 Ang.
                                                    x,1+y,z  =      1_565 Check
PLAT720_ALERT_4_G Number of Unusual/Non-Standard Labels ..........          1 Note
              H9AB
PLAT912_ALERT_4_G Missing # of FCF Reflections Above STh/L=  0.600         12 Note
PLAT941_ALERT_3_G Average HKL Measurement Multiplicity ...........        1.0 Low
PLAT961_ALERT_5_G Dataset Contains no Negative Intensities .......     Please Check
PLAT969_ALERT_5_G The 'Henn et al.' R-Factor-gap value ...........      6.382 Note
              Predicted wR2: Based on SigI**2  2.08 or SHELX Weight 12.13
PLAT978_ALERT_2_G Number C-C Bonds with Positive Residual Density.          5 Info
```

```
   1 ALERT level A = Most likely a serious problem - resolve or explain
   1 ALERT level B = A potentially serious problem, consider carefully
   7 ALERT level C = Check. Ensure it is not caused by an omission or oversight
   7 ALERT level G = General information/check it is not something unexpected

   1 ALERT type 1 CIF construction/syntax error, inconsistent or missing data
   8 ALERT type 2 Indicator that the structure model may be wrong or deficient
   2 ALERT type 3 Indicator that the structure quality may be low
   2 ALERT type 4 Improvement, methodology, query or suggestion
   3 ALERT type 5 Informative message, check
```

Datablock: jas_200m

```
Bond precision:    C-C = 0.0034 A            Wavelength=1.54178

Cell:              a=7.2043(7)      b=6.8196(6)         c=21.542(2)
                   alpha=90         beta=97.624(2)      gamma=90
Temperature:       100 K
```

	Calculated	Reported
Volume	1049.01(17)	1048.99(17)
Space group	P 21/c	P 21/c
Hall group	-P 2ybc	-P 2ybc
Moiety formula	C10 H7 F2 N O2 Te	?
Sum formula	C10 H7 F2 N O2 Te	C10 H7 F2 N O2 Te
Mr	338.77	338.77
Dx,g cm-3	2.145	2.145
Z	4	4
Mu (mm-1)	22.566	22.567
F000	640.0	640.0
F000'	641.60	
h,k,lmax	9,8,27	9,8,27
Nref	2294	2291
Tmin,Tmax	0.089,0.331	0.300,0.750
Tmin'	0.006	

Correction method= # Reported T Limits: Tmin=0.300 Tmax=0.750
AbsCorr = MULTI-SCAN

Data completeness= 0.999 Theta(max)= 79.801

R(reflections)= 0.0192(2285) wR2(reflections)= 0.0503(2291)

S = 1.093 Npar= 146

The following ALERTS were generated. Each ALERT has the format
test-name_ALERT_alert-type_alert-level.
Click on the hyperlinks for more details of the test.

Alert level G

```
PLAT432_ALERT_2_G Short Inter X...Y Contact  Te1      ..O1       .       2.92 Ang.
                                                  -1+x,y,z  =      1_455 Check
PLAT912_ALERT_4_G Missing # of FCF Reflections Above STh/L=  0.600          3 Note
PLAT961_ALERT_5_G Dataset Contains no Negative Intensities .......     Please Check
PLAT969_ALERT_5_G The 'Henn et al.' R-Factor-gap value ..........      2.871 Note
              Predicted wR2: Based on SigI**2  1.75 or SHELX Weight  4.60
PLAT978_ALERT_2_G Number C-C Bonds with Positive Residual Density.          6 Info
```

---

0 **ALERT level A** = Most likely a serious problem - resolve or explain
0 **ALERT level B** = A potentially serious problem, consider carefully
0 **ALERT level C** = Check. Ensure it is not caused by an omission or oversight
5 **ALERT level G** = General information/check it is not something unexpected

0 ALERT type 1 CIF construction/syntax error, inconsistent or missing data

2 ALERT type 2 Indicator that the structure model may be wrong or deficient
0 ALERT type 3 Indicator that the structure quality may be low
1 ALERT type 4 Improvement, methodology, query or suggestion
2 ALERT type 5 Informative message, check

Datablock: jas_207m

Bond precision: C-C = 0.0061 A Wavelength=1.54178

Cell:	a=5.0904(6)	b=11.1361(14)	c=15.0966(18)
	alpha=90	beta=94.154(3)	gamma=90
Temperature:	100 K		

	Calculated	Reported
Volume	853.54(18)	853.54(18)
Space group	P 21/c	P 21/c
Hall group	-P 2ybc	-P 2ybc
Moiety formula	C8 H5 F N2 O Te	?
Sum formula	C8 H5 F N2 O Te	C8 H5 F N2 O Te
Mr	291.74	291.74
Dx,g cm-3	2.270	2.270
Z	4	4
Mu (mm-1)	27.355	27.355
F000	544.0	544.0
F000’	545.16	
h,k,lmax	6,14,19	6,14,19
Nref	1854	1848
Tmin,Tmax	0.088,0.417	0.240,0.750
Tmin’	0.000	

Correction method= # Reported T Limits: Tmin=0.240 Tmax=0.750
AbsCorr = MULTI-SCAN

Data completeness= 0.997 Theta(max)= 79.200

R(reflections)= 0.0373(1609) wR2(reflections)= 0.1080(1848)

S = 1.150 Npar= 126

The following ALERTS were generated. Each ALERT has the format
test-name_ALERT_alert-type_alert-level.
Click on the hyperlinks for more details of the test.

Alert level C

```
PLAT971_ALERT_2_C Check Calcd Resid. Dens.  0.85Ang From Te1            1.62 eA-3
PLAT971_ALERT_2_C Check Calcd Resid. Dens.  0.83Ang From Te1            1.55 eA-3
```

Alert level G

```
PLAT912_ALERT_4_G Missing # of FCF Reflections Above STh/L=  0.600          4 Note
PLAT969_ALERT_5_G The 'Henn et al.' R-Factor-gap value ...........      4.021 Note
              Predicted wR2: Based on SigI**2  2.69 or SHELX Weight  9.39
PLAT978_ALERT_2_G Number C-C Bonds with Positive Residual Density.          3 Info
```

```
   0 ALERT level A = Most likely a serious problem - resolve or explain
   0 ALERT level B = A potentially serious problem, consider carefully
   2 ALERT level C = Check. Ensure it is not caused by an omission or oversight
   3 ALERT level G = General information/check it is not something unexpected

   0 ALERT type 1 CIF construction/syntax error, inconsistent or missing data
   3 ALERT type 2 Indicator that the structure model may be wrong or deficient
   0 ALERT type 3 Indicator that the structure quality may be low
   1 ALERT type 4 Improvement, methodology, query or suggestion
   1 ALERT type 5 Informative message, check
```

Datablock: jas_211m

Bond precision: C-C = 0.0012 A Wavelength=0.71073

Cell:	a=14.3667(5)	b=16.1520(6)	c=11.1475(4)
	alpha=90	beta=94.7333(15)	gamma=90
Temperature:	100 K		

	Calculated	Reported
Volume	2577.97(16)	2577.97(16)
Space group	P 21/c	P 21/c
Hall group	-P 2ybc	-P 2ybc
Moiety formula	C8 H4 F2 N2 O Te	?
Sum formula	C8 H4 F2 N2 O Te	C8 H4 F2 N2 O Te
Mr	309.73	309.73
Dx,g cm-3	2.394	2.394
Z	12	12
Mu (mm-1)	3.458	3.458
F000	1728.0	1728.0
F000'	1722.51	
h,k,lmax	28,32,22	28,32,22
Nref	22036	21949
Tmin,Tmax	0.491,0.579	0.100,0.190
Tmin'	0.350	

```
Correction method= # Reported T Limits: Tmin=0.100 Tmax=0.190
AbsCorr = NUMERICAL

Data completeness= 0.996            Theta(max)= 45.666

                                                        wR2(reflections)=
R(reflections)= 0.0207( 18662)                          0.0517( 21949)

S = 1.080                  Npar= 403
```

The following ALERTS were generated. Each ALERT has the format
test-name_ALERT_alert-type_alert-level.
Click on the hyperlinks for more details of the test.

Alert level B

```
PLAT934_ALERT_3_B Number of (Iobs-Icalc)/Sigma(W) > 10 Outliers ..          4 Check
                3  3  1,  -3  3  2,  -3  3  3,   5  3  3,
```

Author Response: ?? shelxl lst-file list only two at low theta. Likely diffuse scattering of the mounting material or ice.

Alert level C

```
PLAT230_ALERT_2_C Hirshfeld Test Diff for    Te1_1    --C1_1     .        5.5 s.u.
PLAT230_ALERT_2_C Hirshfeld Test Diff for    Te1_3    --C1_3     .        6.0 s.u.
PLAT972_ALERT_2_C Check Calcd Resid. Dens.  0.38Ang From Te1_2          -1.84 eA-3
PLAT972_ALERT_2_C Check Calcd Resid. Dens.  0.20Ang From Te1_1          -1.79 eA-3
PLAT972_ALERT_2_C Check Calcd Resid. Dens.  0.53Ang From Te1_1          -1.79 eA-3
PLAT972_ALERT_2_C Check Calcd Resid. Dens.  0.33Ang From Te1_3          -1.61 eA-3
PLAT972_ALERT_2_C Check Calcd Resid. Dens.  0.45Ang From Te1_3          -1.60 eA-3
PLAT977_ALERT_2_C Check Negative Difference Density on H2A_1     .      -0.32 eA-3
PLAT977_ALERT_2_C Check Negative Difference Density on H3_1      .      -0.85 eA-3
PLAT977_ALERT_2_C Check Negative Difference Density on H6_1      .      -0.32 eA-3
PLAT977_ALERT_2_C Check Negative Difference Density on H2A_2     .      -0.32 eA-3
PLAT977_ALERT_2_C Check Negative Difference Density on H3_2      .      -0.89 eA-3
PLAT977_ALERT_2_C Check Negative Difference Density on H3_3      .      -0.85 eA-3
```

Alert level G

```
PLAT432_ALERT_2_G Short Inter X...Y Contact  Te1_1    ..F1_3     .       3.31 Ang.
                                              x,3/2-y,1/2+z  =     4_576 Check
PLAT432_ALERT_2_G Short Inter X...Y Contact  Te1_1    ..N1_1     .       3.37 Ang.
                                              x,3/2-y,1/2+z  =     4_576 Check
PLAT432_ALERT_2_G Short Inter X...Y Contact  Te1_2    ..N1_3     .       3.24 Ang.
                                           1-x,1/2+y,-1/2-z  =     2_654 Check
PLAT432_ALERT_2_G Short Inter X...Y Contact  Te1_3    ..F1_1     .       3.31 Ang.
                                            1+x,3/2-y,1/2+z  =     4_676 Check
PLAT910_ALERT_3_G Missing FCF Reflection(s) Below Theta(Min)[Deg]=       2.22 Note
                1  0  0,   1  1  0,
PLAT912_ALERT_4_G Missing # of FCF Reflections Above STh/L=  0.600         85 Note
PLAT933_ALERT_2_G Number of HKL-OMIT Records in Embedded .res File          1 Note
```

```
                1  0  0,
PLAT969_ALERT_5_G The 'Henn et al.' R-Factor-gap value ..........      2.561 Note
              Predicted wR2: Based on SigI**2  2.03 or SHELX Weight  4.78
PLAT978_ALERT_2_G Number C-C Bonds with Positive Residual Density.        14 Info
```

```
   0 ALERT level A = Most likely a serious problem - resolve or explain
   1 ALERT level B = A potentially serious problem, consider carefully
  13 ALERT level C = Check. Ensure it is not caused by an omission or oversight
   9 ALERT level G = General information/check it is not something unexpected

   0 ALERT type 1 CIF construction/syntax error, inconsistent or missing data
  19 ALERT type 2 Indicator that the structure model may be wrong or deficient
   2 ALERT type 3 Indicator that the structure quality may be low
   1 ALERT type 4 Improvement, methodology, query or suggestion
   1 ALERT type 5 Informative message, check
```

It is advisable to attempt to resolve as many as possible of the alerts in all categories. Often the minor alerts point to easily fixed oversights, errors and omissions in your CIF or refinement strategy, so attention to these fine details can be worthwhile. It is up to the individual to critically assess their own results and, if necessary, seek expert advice.

PLATON version of 04/06/2025; check.def file version of 30/05/2025

---

duplicate check

A reduced cell check using CCDC's cellCheckCSD service has found that one or more structures in this CIF are similar to those previously published in the CSD.

DATABLOCK: sm_151m

- CSD Refcode: XOGVOH

  CCDC

  [Cell Parameters for XOGVOH: 5.8733,11.2764,23.2542(90,90,90)]

Datablock sm_410pym - ellipsoid plot

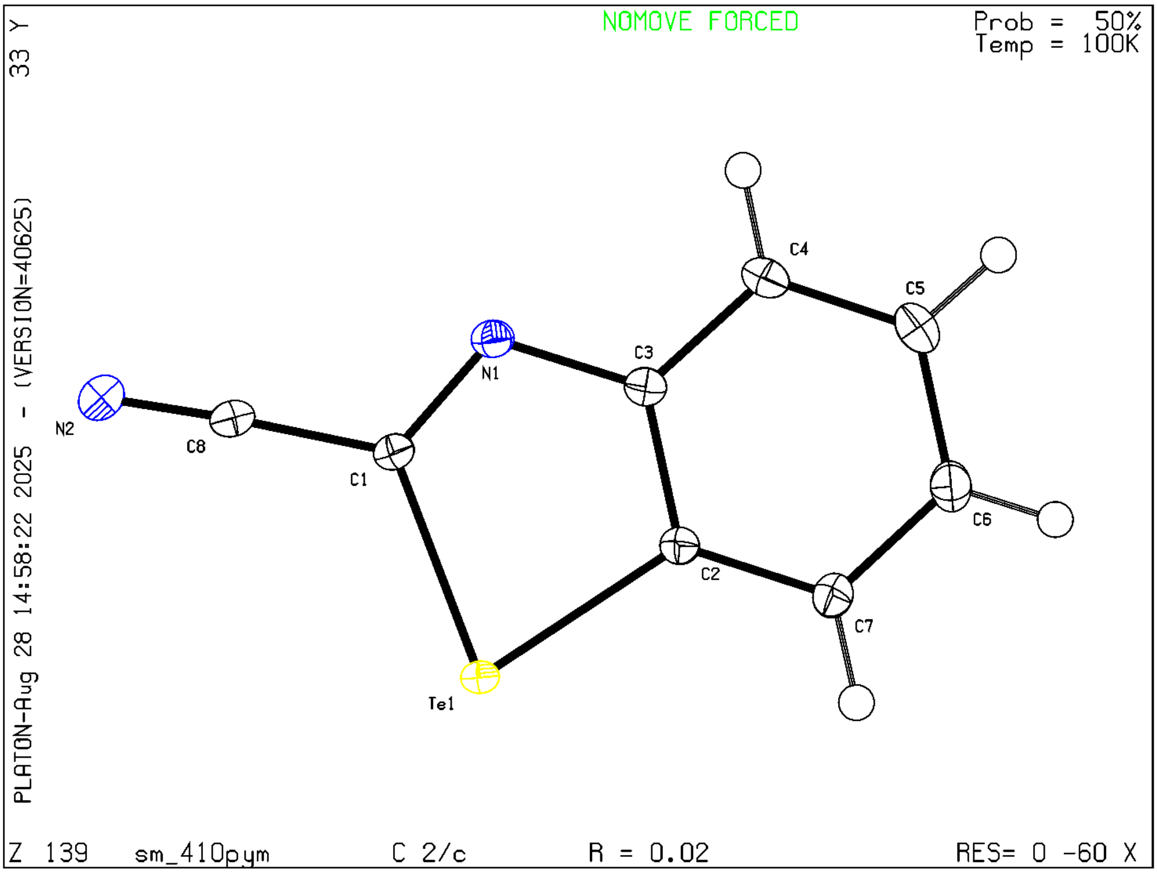

Datablock jas_214m - ellipsoid plot

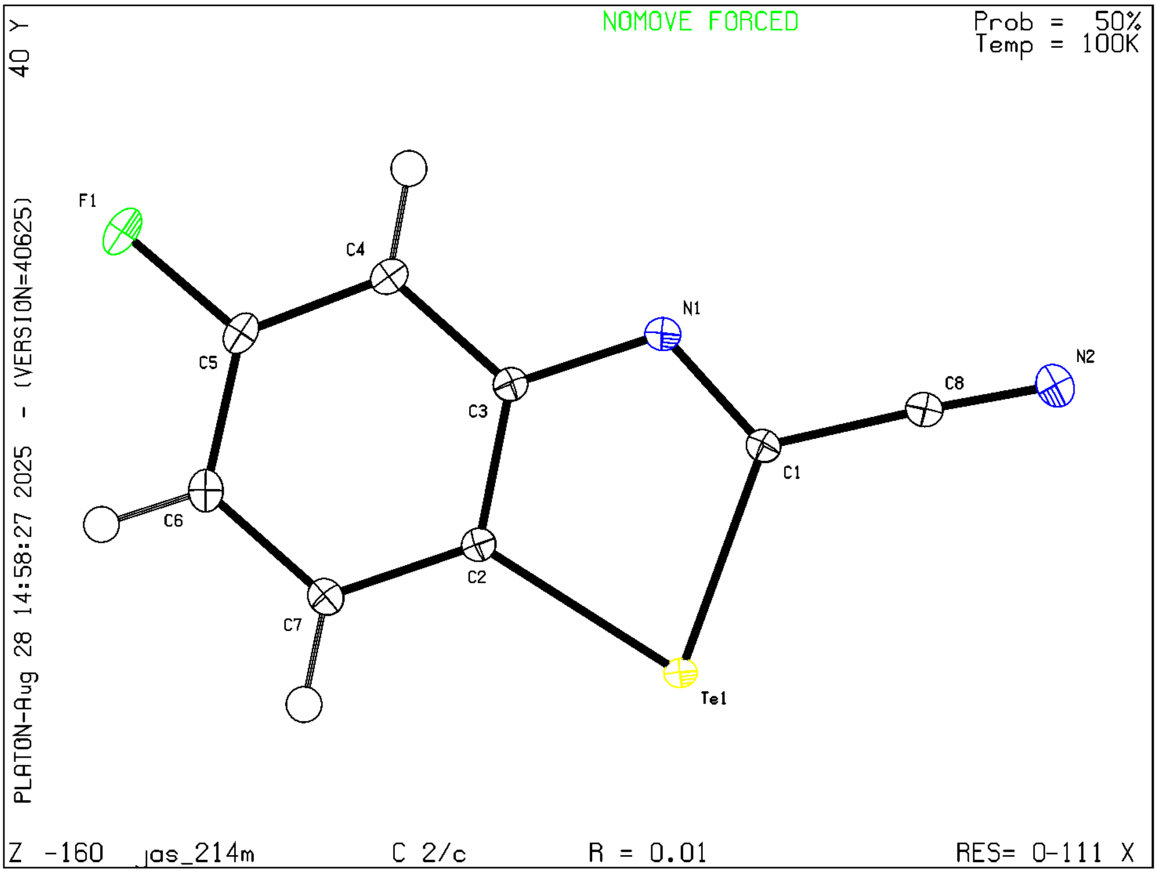

Datablock jas_209m - ellipsoid plot

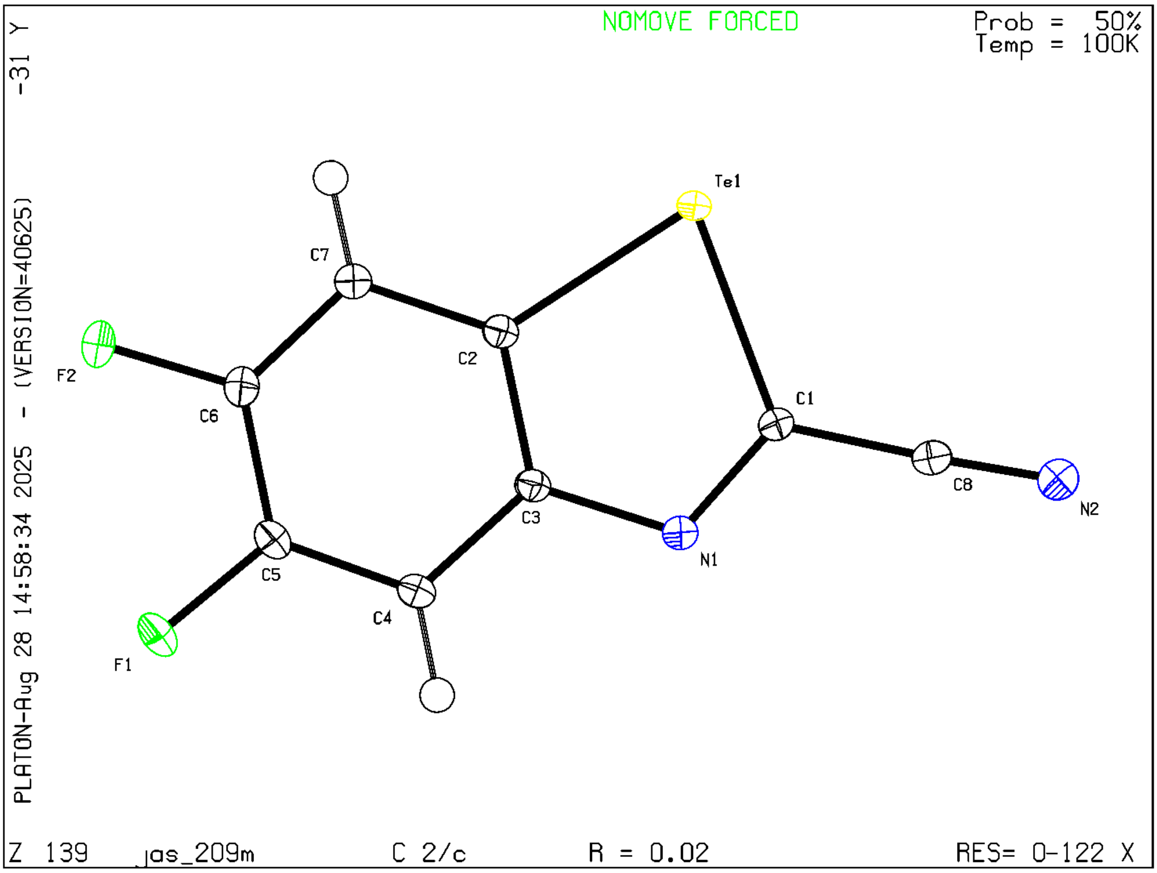

Datablock sm_151m - ellipsoid plot

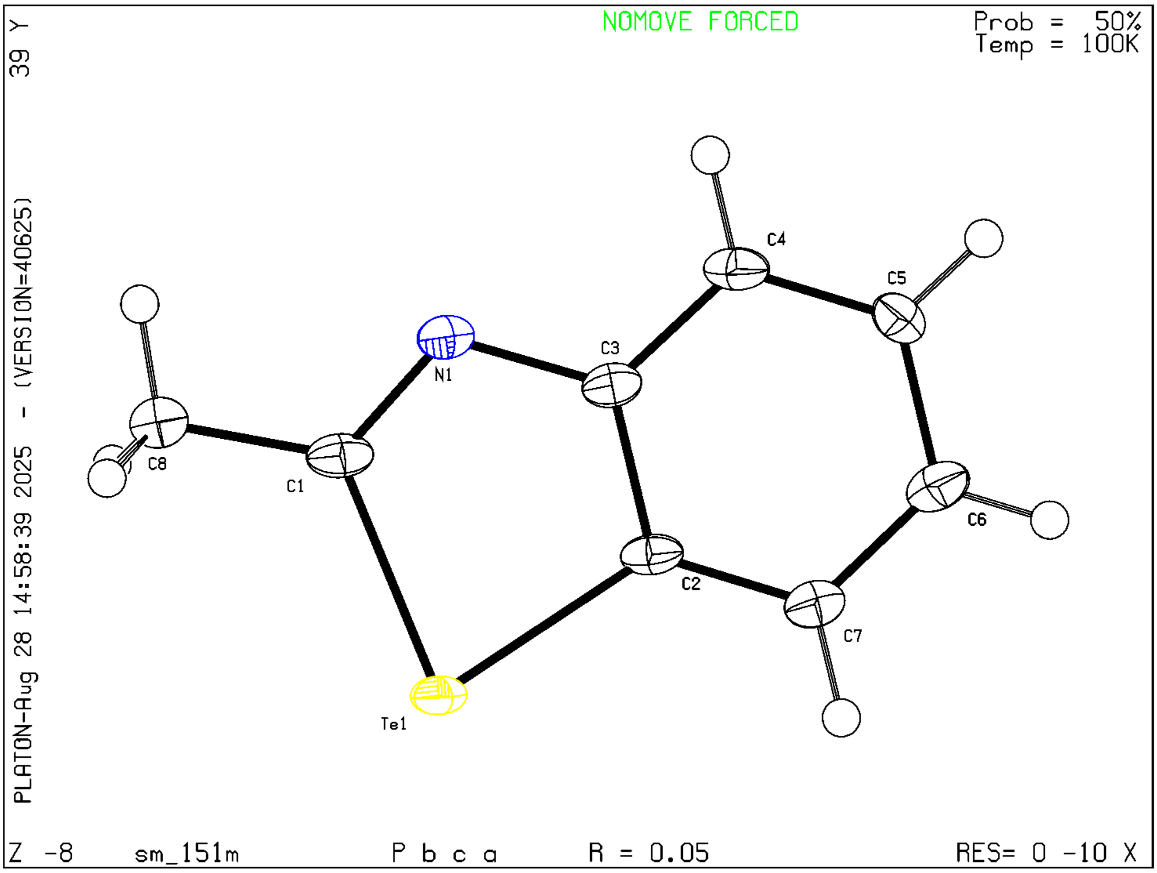

Datablock jas_117m - ellipsoid plot

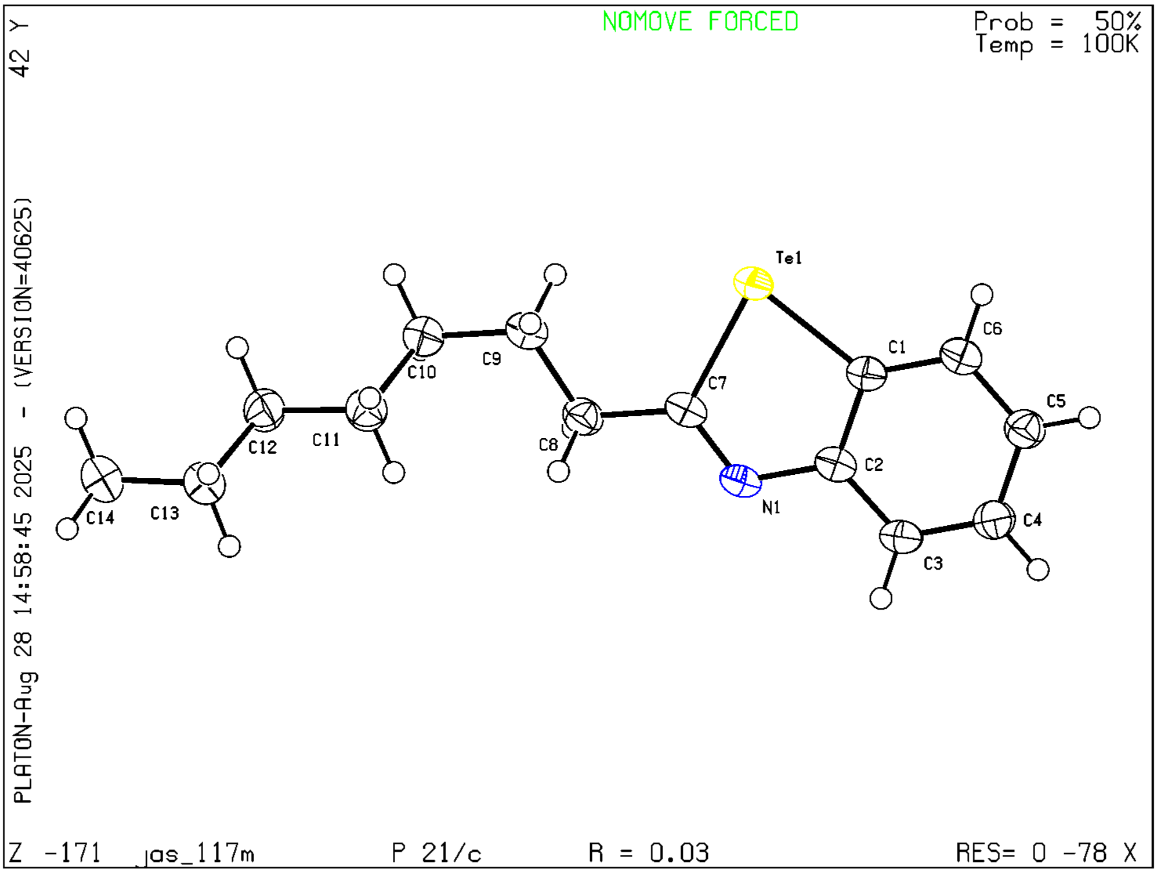

Datablock jas_199_tw4 - ellipsoid plot

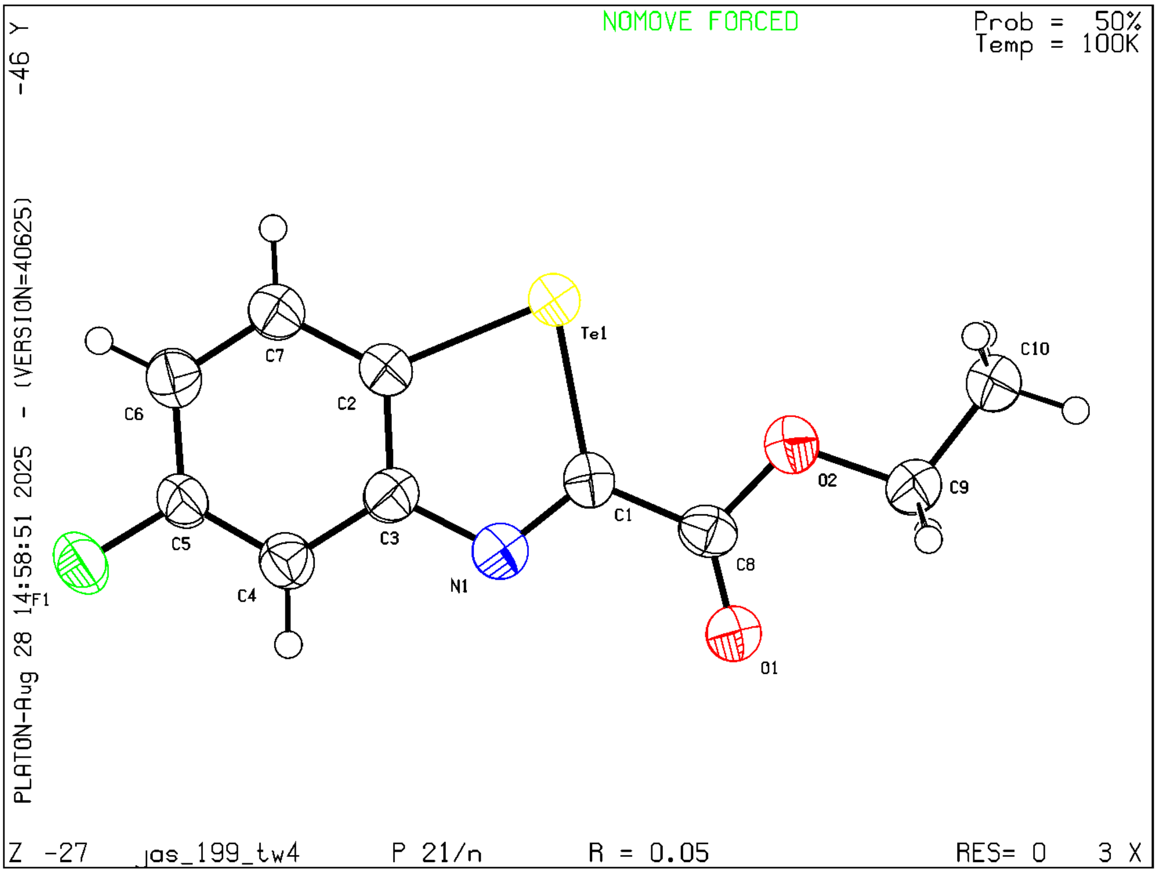

Datablock jas_200m - ellipsoid plot

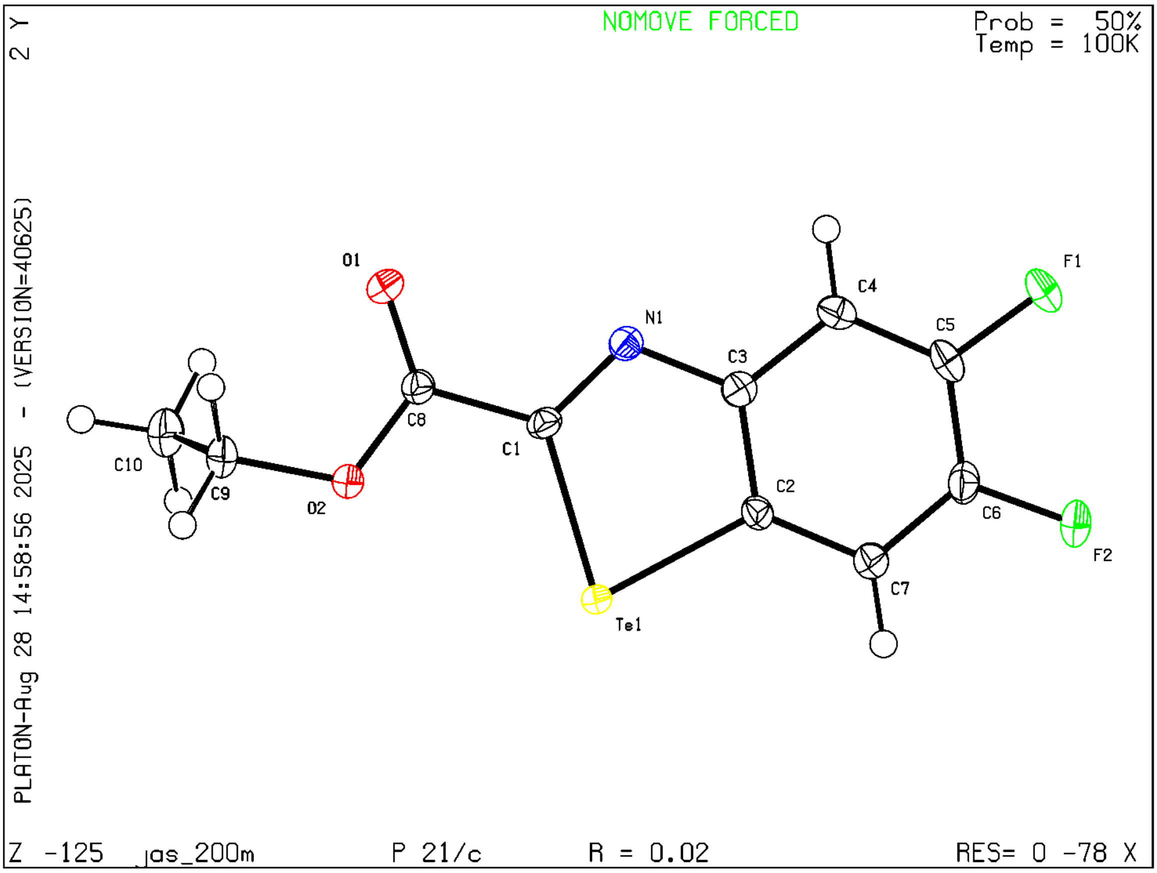

Datablock jas_207m - ellipsoid plot

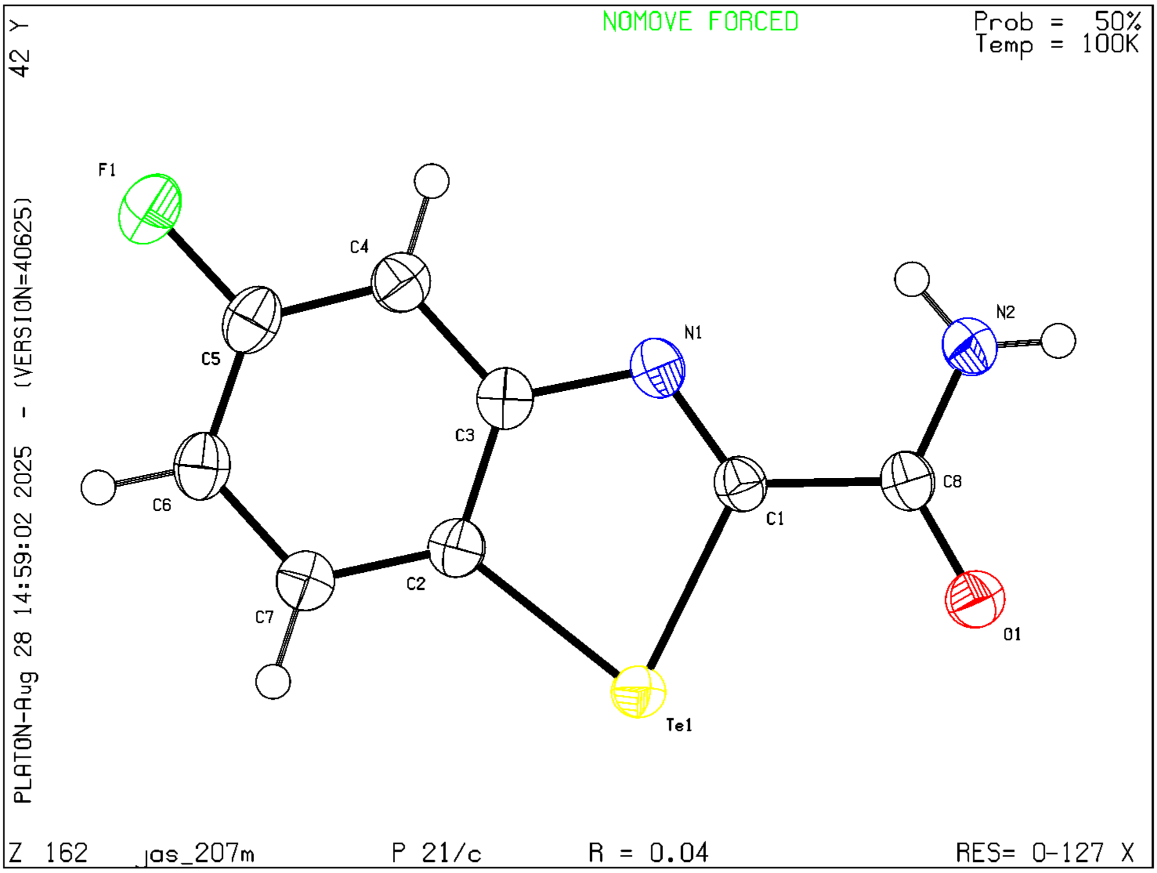

Datablock jas_211m - ellipsoid plot

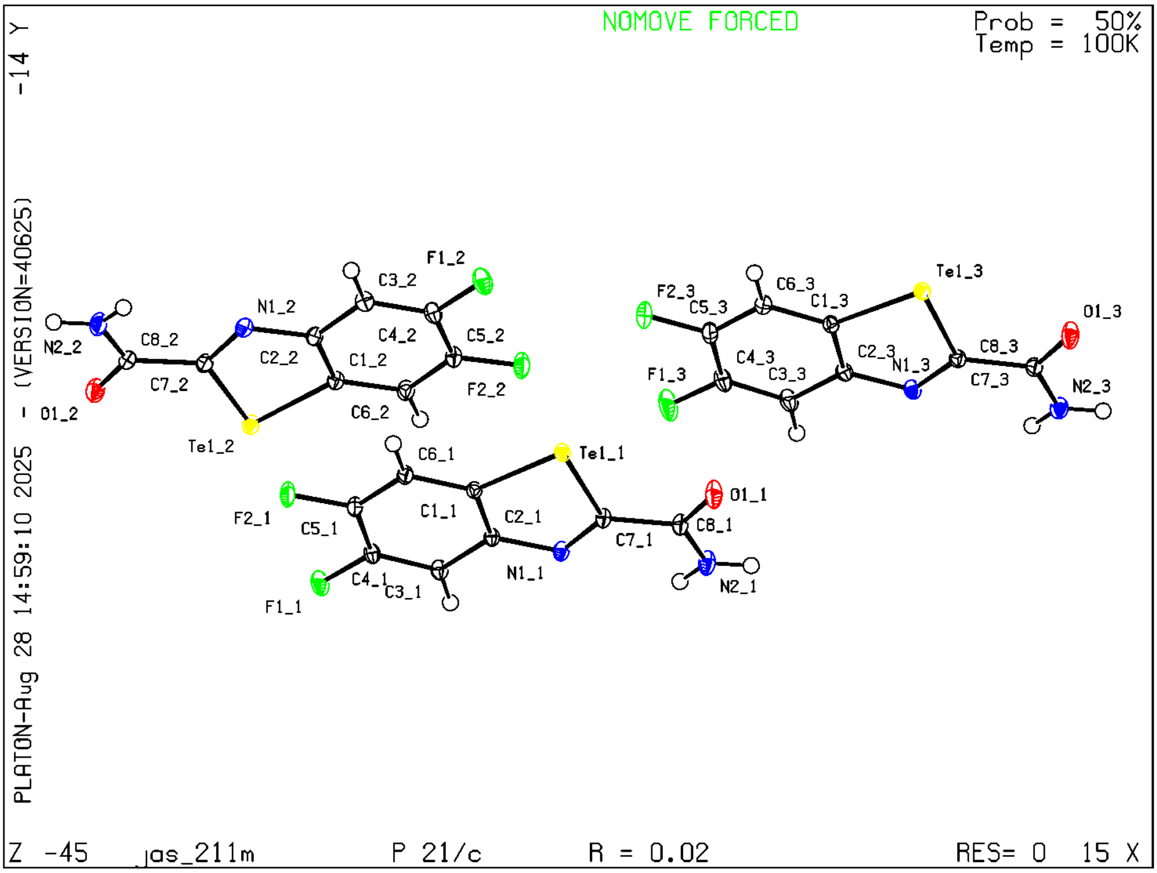

Supplement: Supplementary file 2 — Supporting Information [file CHEM-31-e02731-s002.zip › checkcif_alle.pdf]
